# Association of Atrial Fibrillation Episode Duration With Arrhythmia Recurrence Following Ablation: A Secondary Analysis of a Randomized Clinical Trial

**DOI:** 10.1001/jamanetworkopen.2020.8748

**Published:** 2020-07-02

**Authors:** Jason G. Andrade, Marc W. Deyell, Atul Verma, Laurent Macle, Jean Champagne, Peter Leong-Sit, Paul Novak, Mariano Badra-Verdu, John Sapp, Paul Khairy, Stanley Nattel

**Affiliations:** 1 Montreal Heart Institute, Department of Medicine, Université de Montréal, Montréal, Quebec, Canada; 2 Department of Medicine, University of British Columbia, Vancouver, British Columbia, Canada; 3 Center for Cardiovascular Innovation, Vancouver, British Columbia, Canada; 4 Southlake Regional Health Center, Newmarket, Ontario, Canada; 5 Université Laval, Quebec City, Quebec, Canada; 6 University of Western Ontario, London, Ontario, Canada; 7 Royal Jubilee Hospital, Victoria, British Columbia, Canada; 8 Centre Hospitalier Universitaire de Sherbrooke, Sherbrooke, Quebec, Canada; 9 Queen Elizabeth II Health Sciences Centre, Dalhousie University, Halifax, Nova Scotia, Canada

## Abstract

**Importance:**

Contemporary guidelines recommend that atrial fibrillation (AF) be classified based on episode duration, with these categories forming the basis of therapeutic recommendations. While pragmatic, these classifications are not based on pathophysiologic processes and may not reflect clinical outcomes.

**Objective:**

To evaluate the association of baseline AF episode duration with post-AF ablation arrhythmia outcomes.

**Design, Setting, and Participants:**

The current study is a secondary analysis of a prospective, parallel-group, multicenter, single-masked randomized clinical trial (the Cryoballoon vs Irrigated Radiofrequency Catheter Ablation: Double Short vs Standard Exposure Duration [CIRCA-DOSE] study), which took place at 8 Canadian centers. Between September 2014 and July 2017, 346 patients older than 18 years with symptomatic AF referred for first catheter ablation were enrolled. All patients received an implantable cardiac monitor at least 30 days before ablation. Data analysis was performed in September 2019.

**Exposure:**

Before ablation, patients were classified based on their longest AF episode. Ablation consisted of circumferential pulmonary vein isolation using standard techniques.

**Main Outcomes and Measures:**

Time to first recurrence of symptomatic or asymptomatic atrial tachyarrhythmia (AF, atrial flutter, or atrial tachycardia) following ablation and AF burden (percentage of time in AF) on preablation and postablation continuous rhythm monitoring.

**Results:**

The study included 346 patients (mean [SD] age, 59 [10] years; 231 [67.7%] men). Overall, 263 patients (76.0%) had AF episode duration of less than 24 hours; 25 (7.2%), 24 to 48 hours; 40 (11.7%), 2 to 7 days; and 18 (5.2%), more than 7 days. Documented recurrence of any atrial tachyarrhythmia following ablation was significantly lower in patients with baseline AF episode duration of less than 24 continuous hours compared with those with longer AF episodes (24 hours vs 24-48 hours: hazard ratio [HR], 0.41; 95% CI, 0.21-0.80; *P* = .009; 24 hours vs 2-7 days: HR, 0.25; 95% CI, 0.14-0.45; *P* < .001; 24 hours vs >7 days: HR, 0.23; 95% CI, 0.09-0.55; *P* < .001). Patients with preablation AF episodes limited to less than 24 continuous hours had a significantly lower median (interquartile range) postablation AF burden (0% [0%-0.1%]) compared with those with AF preablation episodes lasting 2-7 days (0.1% [0%-1.0%]; *P* = .003) and those with AF preablation episodes lasting more than 7 days (1.0% [0%-5.4%]; *P* = .008). There was no significant difference in arrhythmia recurrence or AF burden between the 3 groups with a baseline AF episode duration of longer than 24 hours.

**Conclusions and Relevance:**

In this study, patients with AF episodes limited to less than 24 continuous hours had a significantly lower incidence of arrhythmia recurrence following AF ablation. This suggests that current guidelines for classification of AF may not reflect clinical outcomes.

**Trial Registration:**

ClinicalTrials.gov Identifier: NCT01913522

## Key Points

**Question:**

What is the association between preablation atrial fibrillation (AF) episode duration and arrhythmia recurrence outcomes following AF ablation?

**Findings:**

In this prespecified subanalysis of a randomized clinical trial of 346 patients with symptomatic AF undergoing catheter ablation, patients with AF episodes limited to less than 24 continuous hours had a significantly lower rate of recurrence following an ablation procedure. Arrhythmia recurrence and AF burden after ablation did not differ between patients with persistent AF (episodes lasting >7 days) and those with paroxysmal AF (episodes lasting 24 to 48 hours or 2 to 7 days).

**Meaning:**

The findings of this study suggest that the contemporary definition of paroxysmal AF does not reflect post-AF ablation arrhythmia outcomes.

## Teaser

This secondary analysis of a randomized clinical trial evaluates the association of baseline atrial fibrillation episode duration with post–atrial fibrillation ablation arrhythmia outcomes.

## Introduction

Contemporary North American and European guidelines recommend that the clinical pattern of atrial fibrillation (AF) be classified based on episode duration and persistence, with AF defined as paroxysmal if episode duration is less than 7 days and persistent if the episode duration is 7 days or longer.^1^ These clinically determined patterns of AF have been used to characterize the severity of disease, define patient populations in clinical trials, and form the basis of therapeutic recommendations.^1^ Despite their central role in clinical practice, the historical derivation of these AF patterns was arbitrarily defined and thus may not reflect pathophysiologic processes or clinical outcomes.^2^ The aim of the present study was to evaluate the association between AF episode duration and outcomes following AF ablation.

## Methods

The Cryoballoon vs Irrigated Radiofrequency Catheter Ablation: Double Short vs Standard Exposure Duration (CIRCA-DOSE) study was a multicenter, prospective, parallel-group, single-masked randomized clinical trial, with masked end point ascertainment conducted at 8 clinical centers in Canada. Details of the protocol have been reported previously^3^ and appear in Supplement 1. The study enrolled 346 patients older than 18 years with symptomatic AF refractory to at least 1 class I or class III antiarrhythmic drug referred for a first catheter ablation procedure (eFigure in Supplement 2). Ablation consisted of circumferential pulmonary vein isolation using standard techniques.^3^ The study was performed according to the principles outlined in the Declaration of Helsinki^4^ and approved by the appropriate national authorities and the institutional review committee at each center. All patients provided written informed consent. The original study followed the Consolidated Standards of Reporting Trials (CONSORT) reporting guideline.

All patients underwent insertion of an implantable cardiac monitor (ICM) a minimum of 30 days before AF ablation. The ICM was used to determine arrhythmia recurrence as well as to accurately quantify AF episode duration and burden (defined as percentage of time in AF).

Patients were followed up for 1 year after the ablation procedure with clinical visits, a 12-lead electrocardiogram, and supplementary 24-hour ambulatory electrocardiogram monitoring at 3, 6, and 12 months. Automatic transmissions from the ICM were obtained daily. Arrhythmia events meeting standardized arrhythmia detection settings underwent independent adjudication by a masked committee.

The primary outcome was defined as time to first symptomatic or asymptomatic atrial tachyarrhythmia (AF, atrial flutter, or atrial tachycardia) documented by 12-lead electrocardiogram, 24-hour ambulatory Holter monitor, or ICM between 91 and 365 days after ablation or a repeated ablation procedure at any time. Postablation AF burden, ie, the proportion of the monitored interval that a patient was in AF, was a secondary outcome.

Patients were stratified based on their longest AF episode duration detected on preablation ICM monitoring. They were split into the following groups: less than 24 hours, 24 to 48 hours, 2 to 7 days, and more than 7 days.^1,2,5^

### Statistical Analysis

Survival curves for time to first arrhythmia recurrence were estimated by the Kaplan-Meier method and compared by the Mantel-Cox test, with unadjusted hazard ratios (HR) evaluated by Cochran-Mantel-Haenszel method. Multivariable logistic regression accounting for clinically important baseline characteristics, including study site, age, sex, weight, AF duration, and number of prior antiarrhythmic drugs. Differences in AF burden were evaluated using Kruskal-Wallis test. Data analysis was performed in September 2019. Analyses were performed using SAS software version 9.4 (SAS Institute). All statistical tests and confidence intervals were 2-sided, with a significance level of *P* < .05.

## Results

A total of 346 patients were enrolled between September 2014 and July 2017, with a mean (SD) age of 59 (10) years, 231 (67.7%) men, and 238 (68.8%) receiving antiarrhythmic drugs during the preablation period. Continuous rhythm monitoring via ICM was performed for a median (interquartile range) of 73.5 (50.0-98.3) days before AF ablation. Characteristics of the 4 groups, stratified by longest AF episode duration, are presented in the Table. Overall, 263 patients (76.0%) had AF episode duration of less than 24 hours; 25 (7.2%), 24 to 48 hours; 40 (11.7%), 2 to 7 days; and 18 (5.2%), more than 7 days.

**Table.** Study Sample Characteristics

Characteristic	No. (%)				*P *value
	<24 h (n = 263)	24-48 h (n = 25)	2-7 d (n = 40)	>7 d (n = 18)	
Age, mean (SD), y	58.2 (10.1)	62.9 (1.7)	60.5 (7.0)	57.4 (11.6)	.09
Women	87 (33.1)	10 (40.0)	17 (42.5)	1 (5.6)	.04
BMI, mean (SD)	28.7 (5.0)	28.8 (4.2)	31.6 (7.0)	29.3 (4.5)	.10
AFEQT score at enrollment, mean (SD)^a^	53.8 (22.1)	53.9 (15.9)	53.7 (20.4)	61.3 (20.1)	.54
AAD use during preablation monitoring period	179 (68.6)	17 (68.0)	33 (82.5)	9 (50.0)	.09
AADs failed before enrollment, median (IQR), No.	2.0 (1.0-2.0)	2.0 (1.0-3.0)	2.0 (1.0-2.8)	2.0 (1.0-3.0)	.17
Emergency department visits preablation, median (IQR), No.	1.0 (0.0-3.0)	1.5 (.0-4.0)	1.0 (0.0-2.0)	1.0 (0.0-2.3)	.73
Cardioversions preablation, median (IQR), No.	2.0 (1.0-3.0)	1.0 (1.0-4.8)	2.0 (1.0-3.5)	4.0 (3.0-6.5)	.34
CHA_2_DS_2_-VASc score, median (IQR)^b^	1.0 (0.0-2.0)	2.0 (1.0-2.0)	1.0 (0.0-2.0)	0.0 (0.0-1.3)	.06
Congestive heart failure	2 (0.8)	2 (8.0)	1 (2.5)	1 (5.6)	.03
Hypertension	91 (34.6)	9 (36.0)	14 (35.0)	6 (33.3)	.99
Diabetes	24 (9.1)	2 (8.0)	2 (5)	1 (5.6)	.81
Ischemic heart disease	20 (7.6)	1 (4.0)	3 (7.5)	1 (5.6)	.91
Chronic obstructive pulmonary disease	5 (1.9)	0	1 (2.5)	1 (5.6)	.63
Sleep apnea	33 (12.5)	1 (4.0)	8 (20.0)	3 (16.7)	.29
Previous stroke or transient ischemic attack	9 (3.4)	1 (4.0)	1 (2.5)	0	.86
Tobacco use	14 (5.3)	0	3 (7.5)	1 (5.6)	.61
Left atrial, mean (SD)					
Size, parasternal long axis, mm	37.5 (8.6)	35.1 (11.0)	37.2 (9.6)	43.5 (6.1)	.02
Volume, mL/m^2^	34.3 (14.3)	32.0 (16.4)	36.9 (12.8)	44.4 (26.4)	.20
Left ventricular ejection fraction, mean (SD), %	59.5 (5.7)	6.1 (6.5)	57.5 (7.5)	58.0 (7.5)	.18
Diastolic dysfunction	35 (13.8)	2 (8.0)	3 (7.5)	4 (22.2)	.39

Abbreviations: AAD, antiarrhythmic drug; AFEQT, Atrial Fibrillation Effect on Quality of Life; BMI, body mass index (calculated as weight in kilograms divided by height in meters squared); IQR, interquartile range.

^a^ AFEQT score is a disease-specific quality of life score, with 0 representing the worst and 100 representing the best possible quality of life (ie, no impairment due to AF).

^b^ The CHA_2_DS_2_-VASc score is a clinical estimation of the risk of stroke in patients with atrial fibrillation, with 2 points assigned for a history of stroke or transient ischemic attack (S_2_) or age (A_2_) older than 75 years and 1 point each for an age (A) of 65 to 74 years or a history of congestive heart failure (C), hypertension (H), diabetes (D), vascular disease (V) (myocardial infarction and peripheral artery disease), and female sex (sex category [Sc]). Scores range from 0 to 9, and higher scores indicate a greater risk.

**Figure 1.**
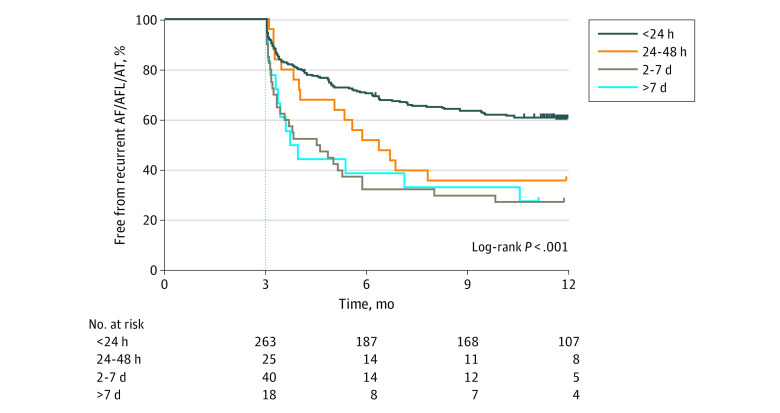
Freedom From Atrial Fibrillation (AF), Atrial Flutter (AFL), and Atrial Tachycardia (AT) After a Single Ablation Procedure, Stratified by the Longest AF Episode Recorded on Preablation Monitoring

Freedom from documented recurrence of any (symptomatic or asymptomatic) atrial tachyarrhythmia is presented in Figure 1. Patients with AF episodes limited to less than 24 continuous hours on preablation monitoring were significantly more likely to be free from recurrent AF, atrial flutter, and atrial tachycardia compared with those with longer AF episode durations (<24 hours: 159 [60.5%]; 24-48 hours: 9 [36.0%]; 2-7 days: 11 [27.5%]; >7 days: 5 [27.8%]; *P* < .001; <24 hours vs 24-48 hours: unadjusted HR, 0.41; 95% CI, 0.21-0.80; *P* = .009; 24 hours vs 2-7 days: unadjusted HR, 0.25; 95% CI, 0.14-0.45; *P* < .001; 24 hours vs >7 days: unadjusted HR, 0.23; 95% CI, 0.09-0.55; *P* < .001). In contrast, there was no significant difference between groups with a baseline AF episode duration of greater than 24 hours. Multivariable analysis identified left atrial enlargement and baseline AF episode durations of longer than 24 continuous hours as independent factors associated with arrhythmia recurrence after ablation (odds ratio, 1.92; 95% CI, 1.11-3.34 and odds ratio, 3.36; 95% CI, 1.79-6.53; respectively).

**Figure 2.**
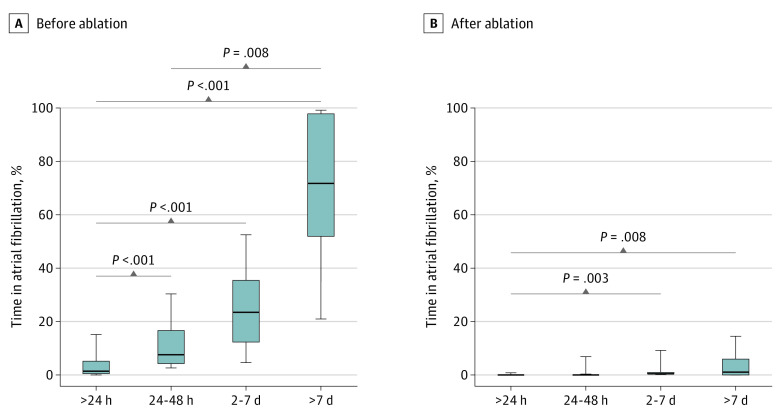
Atrial Fibrillation Burden Before and After Ablation, Stratified by Longest Atrial Fibrillation Episode Duration Recorded on Preablation Monitoring Atrial fibrillation burden defined as percentage of time in atrial fibrillation. The center line indicates the median; the bottom and the top of the box, the 25th and 75th percentiles, respectively, and the lower and upper whiskers, the 10th and 90th percentiles, respectively.

The association between preablation and postablation AF burden according to the longest preablation AF episode duration is depicted in Figure 2. Median (interquartile range) preablation AF burden increased significantly relative to the single longest recorded AF episode duration (<24 hours: 1.3% [0.2%-5.3%]; 24-48 hours: 7.4% [3.5%-16.7%]; 2-7 days: 23.3% [11.7%-35.5%]; >7 days: 71.8% [51.7%-98.0%]; *P* < .001). Following ablation, the AF burden decreased significantly in all groups, with a similar magnitude of improvement between groups (median within-patient reduction from baseline of among patients with episodes <24 hours: 100% [95% CI, 87.9%-100%]; 24-48 hours: 99.7% [95% CI, 92.4%-100%]; 2-7 days: 99.7% [95% CI, 90.1%-100%]; >7 days: 98.7% [95% CI, 90.0%-100%]; *P* < .001). Patients with preablation AF episodes shorter than 24 continuous hours had a significantly lower median (interquartile range) postablation AF burden (0% [0%-0.1%) compared with those with preablation AF episodes lasting 2 to 7 days (0.1% [0%-1.0%]; *P* = .003) and those with preablation AF episodes lasting more than 7 days (1.0% [0%-5.4%]; *P* = .008).

## Discussion

Historically, paroxysmal AF has been pragmatically but arbitrarily defined as “attacks of arrhythmia lasting from 2 minutes to 7 days.”^2,5^ However, more recent evidence suggests that this classification may not reflect the pathophysiologic process underlying AF or the complications associated with AF. Important changes in AF-related electrical and structural remodeling occur during periods as short as 24 hours, achieving a steady state as early as 48 hours after the onset of an AF episode.^6,7^ This parallels the clinical observation that the efficacy of acute pharmacologic conversion of AF decreases substantially after 24 to 48 continuous hours of AF.^8,9,10,11^ Likewise, the risk of ischemic stroke or systemic embolism has been observed to increase substantially only among those with subclinical AF episodes of longer than 24 hours, leading some groups to propose more than 24 hours of continuous AF as a highly relevant threshold for oral anticoagulation initiation.^12,13,14,15^

The current study adds to this body of evidence by demonstrating that patients with AF episode durations longer than 24 hours had a significantly greater recurrence of AF after ablation and a significantly higher postablation AF burden compared with patients with shorter-duration AF. These observations are consistent with the idea that intervention early in the natural history of AF, before the onset of the progressive pathophysiologic and anatomic changes associated with the arrhythmia, may improve clinical outcomes.^16,17,18^

Moreover, our findings reemphasize the arbitrary nature of the present classification of AF. While the current definitions of AF clearly have some meaning, they are not based on detailed analyses of pathophysiologic processes or on clinically relevant outcomes. In our study, we found no significant difference in arrhythmia outcomes in patients with AF episodes limited to 24 to 48 hours and 2 to 7 days compared with those lasting more than 7 days. Given the significantly better postablation outcomes among the subset of patients with AF episodes limited to less than 24 continuous hours, consideration should be given to 24 hours of continuous AF as a threshold for defining AF persistence.

### Limitations

This study has limitations. It is a subanalysis of a prospective randomized clinical trial. Groups were defined based on the longest AF episode duration observed on continuous monitoring before ablation. While attempts were made to account for baseline differences, it is possible that residual confounders may have influenced the results. In addition, most patients were actively receiving antiarrhythmic drugs during the preablation monitoring period, which may have influenced episode duration. Furthermore, while the relative differences in postablation AF burden were significant, the differences in absolute postablation AF burden were small. As such, the clinical effect is uncertain given that most cases had a low burden of AF after ablation.

## Conclusions

In this study, patients with AF episodes limited to less than 24 continuous hours before ablation had a significantly lower arrhythmia recurrence following ablation. These results suggest that the current definition of paroxysmal AF should be reevaluated.

## Notes

Supplement 1. **Trial Protocol and Statistical Analysis Plan**

Supplement 2. **eFigure.** Study Flow Diagram

Supplement 3. **Data Sharing Statement**

## References

[1] Andrade JG, Macle L, Nattel S, Verma A, Cairns J Contemporary atrial fibrillation management: a comparison of the current AHA/ACC/HRS, CCS, and ESC guidelines. Can J Cardiol. 2017;33(8):965-976. doi:10.1016/j.cjca.2017.06.002 28754397

[2] Lévy S, Novella P, Ricard P, Paganelli F Paroxysmal atrial fibrillation: a need for classification. J Cardiovasc Electrophysiol. 1995;6(1):69-74. doi:10.1111/j.1540-8167.1995.tb00758.x 7743011

[3] Andrade JG, Deyell MW, Badra M, Randomised clinical trial of cryoballoon versus irrigated radio frequency catheter ablation for atrial fibrillation—the effect of double short versus standard exposure cryoablation duration during pulmonary vein isolation (CIRCA-DOSE): methods and rationale. BMJ Open. 2017;7(10):e017970. doi:10.1136/bmjopen-2017-017970 28982836 PMC5639989

[4] World Medical Association World Medical Association Declaration of Helsinki: ethical principles for medical research involving human subjects. JAMA. 2013;310(20):2191-2194. doi:10.1001/jama.2013.281053 24141714

[5] Friedlander RD, Levine SA Auricular fibrillation and flutter without evidence of organic heart disease. N Engl J Med. 1934;211:624-629. doi:10.1056/NEJM193410042111405

[6] Wijffels MC, Kirchhof CJ, Dorland R, Allessie MA Atrial fibrillation begets atrial fibrillation: a study in awake chronically instrumented goats. Circulation. 1995;92(7):1954-1968. doi:10.1161/01.CIR.92.7.1954 7671380

[7] Fareh S, Villemaire C, Nattel S Importance of refractoriness heterogeneity in the enhanced vulnerability to atrial fibrillation induction caused by tachycardia-induced atrial electrical remodeling. Circulation. 1998;98(20):2202-2209. doi:10.1161/01.CIR.98.20.2202 9815876

[8] Crijns HJ, van Wijk LM, van Gilst WH, Kingma JH, van Gelder IC, Lie KI Acute conversion of atrial fibrillation to sinus rhythm: clinical efficacy of flecainide acetate: comparison of two regimens. Eur Heart J. 1988;9(6):634-638. doi:10.1093/oxfordjournals.eurheartj.a062553 3137061

[9] Roy D, Pratt CM, Torp-Pedersen C, ; Atrial Arrhythmia Conversion Trial Investigators Vernakalant hydrochloride for rapid conversion of atrial fibrillation: a phase 3, randomized, placebo-controlled trial. Circulation. 2008;117(12):1518-1525. doi:10.1161/CIRCULATIONAHA.107.723866 18332267

[10] Suttorp MJ, Kingma JH, Jessurun ER, Lie-A-Huen L, van Hemel NM, Lie KI The value of class IC antiarrhythmic drugs for acute conversion of paroxysmal atrial fibrillation or flutter to sinus rhythm. J Am Coll Cardiol. 1990;16(7):1722-1727. doi:10.1016/0735-1097(90)90326-K 2123909

[11] Crozier IG, Ikram H, Kenealy M, Levy L Flecainide acetate for conversion of acute supraventricular tachycardia to sinus rhythm. Am J Cardiol. 1987;59(6):607-609. doi:10.1016/0002-9149(87)91178-7 3103410

[12] Van Gelder IC, Healey JS, Crijns HJGM, Duration of device-detected subclinical atrial fibrillation and occurrence of stroke in ASSERT. Eur Heart J. 2017;38(17):1339-1344. doi:10.1093/eurheartj/ehx042 28329139

[13] Capucci A, Santini M, Padeletti L, ; Italian AT500 Registry Investigators Monitored atrial fibrillation duration predicts arterial embolic events in patients suffering from bradycardia and atrial fibrillation implanted with antitachycardia pacemakers. J Am Coll Cardiol. 2005;46(10):1913-1920. doi:10.1016/j.jacc.2005.07.044 16286180

[14] Boriani G, Botto GL, Padeletti L, ; Italian AT-500 Registry Investigators Improving stroke risk stratification using the CHADS_2_ and CHA_2_DS_2_-VASc risk scores in patients with paroxysmal atrial fibrillation by continuous arrhythmia burden monitoring. Stroke. 2011;42(6):1768-1770. doi:10.1161/STROKEAHA.110.609297 21493904

[15] Andrade JG, Mitchell LB Periprocedural anticoagulation for cardioversion of acute onset atrial fibrillation and flutter: evidence base for current guidelines. Can J Cardiol. 2019;35(10):1301-1310. doi:10.1016/j.cjca.2019.06.006 31601412

[16] Padfield GJ, Steinberg C, Swampillai J, Progression of paroxysmal to persistent atrial fibrillation: 10-year follow-up in the Canadian Registry of Atrial Fibrillation. Heart Rhythm. 2017;14(6):801-807. doi:10.1016/j.hrthm.2017.01.038 28232263

[17] Nattel S, Guasch E, Savelieva I, Early management of atrial fibrillation to prevent cardiovascular complications. Eur Heart J. 2014;35(22):1448-1456. doi:10.1093/eurheartj/ehu028 24536084

[18] Andrade JG, Champagne J, Deyell MW, ; EARLY-AF Study Investigators A randomized clinical trial of early invasive intervention for atrial fibrillation (EARLY-AF): methods and rationale. Am Heart J. 2018;206:94-104. doi:10.1016/j.ahj.2018.05.020 30342299

