# Supplementary material for: Association of Atrial Fibrillation Episode Duration With Arrhythmia Recurrence Following Ablation: A Secondary Analysis of a Randomized Clinical Trial
Source: JAMA Netw Open. 2020 Jul 2;3(7):e208748. doi: 10.1001/jamanetworkopen.2020.8748 (PMC7333024; doi:10.1001/jamanetworkopen.2020.8748)
Supplement: Supplement 1. — Trial Protocol and Statistical Analysis Plan [file jamanetwopen-3-e208748-s001.pdf]

Cryoballoon vs. Irrigated Radiofrequency Catheter Ablation for Atrial Fibrillation: A multicenter randomized clinical trial

This supplement contains the following items:

1. Original/Final protocol, and two addendums.

2. Original/Final statistical analysis plan (no changes were made).

Cryoballoon vs. Irrigated Radiofrequency Catheter Ablation: The effect of Double Short vs. Standard Exposure Cryoablation Duration During Pulmonary Vein Isolation

The CIRCA-DOSE Study

Protocol Number: **2055**

Version: **Final version, June 27th, 2014**

Table of Content

1. INTRODUCTION 6
1.1 Background 6
1.2 Study Objective 10
1.3 Hypothesis to be tested 10
2. STUDY PURPOSE 11
2.1 Study Population 11
2.1.1 Source and number of patients 11
2.1.2 Eligibility Criteria 11
2.1.2.1. Inclusion criteria 11
2.1.2.2. Exclusion criteria 11
2.2 Study Design 12
2.2.1. Trial design 12
2.2.2. Duration of treatment and follow-up period 12
2.2.3. Baseline Data Collection 12
2.2.4. Implantable loop recorder (ILR) 13
2.2.5. Randomization 14
2.2.6. The Ablation Procedure 14
2.2.6.1. Pre-Procedure Imaging 14
2.2.6.2. Para-procedure patient management 14
2.2.6.3. Radiofrequency catheter ablation (RFCA) (Group 1) 15
2.2.6.4. Cryoballoon Ablation (CBA) (Group 2 and Group 3) 15
2.2.6.5. Procedural Success 17
2.2.6.6. Post procedural Care 17
2.2.7. Follow-up period 18
2.2.7.1. Post ablation blanking period 18
2.2.7.2. Anticoagulation 18
2.2.7.3. Antiarrhythmic Drugs 18
2.2.7.4. Arrhythmia recurrence 18
2.2.7.5. Follow-up visits 18
3. STUDY METHODOLOGY 19
3.1. Primary and secondary outcomes measures 19
3.1.1. Primary endpoint 19
3.2. Safety Monitoring 20
3.2.1. Description of risks to the safety of participants involved in the trial 20

3.2.2. Adverse events 20
3.2.3. Serious adverse events 21
3.2.4. Major complications associated with AF ablation 21
3.3. Study termination 21
3.4. Informed consent 22
3.5. Data Collection 22
3.5.1. Implantable Loop Recorder 22
3.5.2. Case report form 22
4. STATISTICAL CONSIDERATIONS 22
4.1. Protection from bias 22
4.2. Statistical Power and Sample size considerations 23
4.3. Recruitment rate 23
4.4. Proposed type of analyses 23
4.5. Proposed frequency of analyses 24
4.6. Planned subgroup analyses 24
5. STUDY ORGANIZATION 24
5.1. Day-to-day trial management 24
5.2. Study Investigators 25
5.3. Role of each principal and co-applicant 25
5.4. Steering Committee 25
5.5. Clinical Events Committee (CEC) 26
6. POST TRIAL KNOWLEDGE DISSEMINATION 26
6.1. Anticipated results and their implications 26
6.2. Plan for Knowledge Dissemination 26
7. GLOSSARY 27
8. REFERENCES 28
APPENDIX A: Timetable of visits and procedures 34
APPENDIX B: Definitions of the Major complications associated with AF Ablation 35
APPENDIX C: Declaration of Helsinki 36

INVESTIGATOR STATEMENT AND SIGNATURE

Protocol # 2055

Cryoballoon vs. Irrigated Radiofrequency Catheter Ablation:
The effect of Double Short vs. Standard Exposure Cryoablation Duration During Pulmonary Vein Isolation

The CIRCA-DOSE Study

I have read the protocol described herein. I agree to comply with all applicable regulations and to conduct the study as described in the protocol.

Site Principal Investigator:

Signature: ________________________________ **Date:** ________________

Printed Name: ______________________________

Site Name: ________________________________

SPONSOR SIGNATURE PAGE

Protocol # 2055

Cryoballoon vs. Irrigated Radiofrequency Catheter Ablation:
The effect of Double Short vs. Standard Exposure Cryoablation Duration During Pulmonary Vein Isolation

The CIRCA-DOSE Study

Sponsor Signature

Jason Andrade, MD<br>Study Principal Investigator / Sponsor	(Signature)	27-JUN-2014<br>(Date)

MHICC Signature

Marie-Claude Guertin, Ph.D.<br>Head, Biostatistics Department, MHICC	(Signature)	01JUL2014<br>(Date)

1. INTRODUCTION

Atrial fibrillation (AF) is the most common sustained cardiac arrhythmia and is associated with reductions in quality of life, functional status, cardiac performance, and overall survival.[1] Catheter ablation, which is centered on electrical isolation of triggering foci within the pulmonary veins (PVI) through circumferential lesions around PV ostia, has been shown to result in sustained improvements in quality of life, decreased hospitalizations and, potentially, improved survival.[2-4] PVI can be accomplished by percutaneous catheter-based thermo-coagulation (burning) with radiofrequency (RF) energy delivery or alternatively by thermo-cooling (freezing) with a cryoballoon catheter.[5] Cryothermal ablation with a cryoballoon catheter offers an efficacious means to achieve PVI that is safer than the established technique. Although cryoballoon ablation has been used in clinical practice for some time, the optimal duration of cryoballoon ablation has not been determined. Moreover, the biophysics of cryo-lesion formation suggests that repeated short freezes ("freeze-thaw-freeze" cycles) may be more efficacious in achieving deep homogenous lesion when compared to prolonged freezing durations.

1.1 Background

Atrial Fibrillation is a major health issue
AF is the most common sustained arrhythmia seen in clinical practice. It is estimated that AF affects nearly 250,000 Canadians, 2.5 million Americans and 6 million Europeans.[1, 6] While AF affects 1-2% of the overall population, the prevalence of AF increases significantly with age, rising from 4% at 60 years to 9% at 80 years.[7, 8] In addition to reductions in quality of life, functional status, and cardiac performance patients with AF have an increased risk of stroke and overall mortality.[1, 9-15] Moreover, AF accounts for the majority of arrhythmia related emergency room visits and hospital admissions, a number that has risen 66% over the past 20 years.[16] Current estimates suggest AF accounts for over 350,000 annual US hospital admissions, and an overall hospitalization rate of 583 per 100,000 Canadians.[6, 17] As a result, AF imposes a significant economic burden on health care systems, with the direct costs of AF management accounting for approximately 1% of total healthcare expenditures.[18] Thus, when combined with the growing proportion of elderly individuals, as well as the increasing prevalence of chronic heart disease, it is clear that AF is poised to become a national public health crisis as it is anticipated that the number of patients with AF will increase 2.5-fold over the next 40 years.[8, 18, 19]

Goals of AF Management
AF is a chronic progressive disease, characterized by exacerbations and remissions. The contemporary management of AF is centered on symptomatic improvement, as well as reduction in the morbidity and mortality associated with AF (i.e., the prevention of tachycardia-induced cardiomyopathy, and stroke or systemic thromboembolism), with consequent reduction in AF-related emergency room visits or hospitalizations.[20] While strategies directed at ventricular rate control are effective in some individuals, a significant proportion of patients remain highly symptomatic despite achievement of adequate control of ventricular rate. For these individuals, restoration and maintenance of sinus rhythm can alleviate symptoms (palpitations, fatigue, exercise intolerance, or symptoms of heart failure) and improve exercise capacity and quality of life. As such, effective treatments that are able to abolish, or greatly reduce the arrhythmia burden are highly desirable.

Sinus Rhythm Maintenance
In the absence of preventative treatment, the recurrence rate of AF is estimated to be 75% over 1 year.[6] As such, the majority of patients desiring a preservation of sinus rhythm will require maintenance therapy. While antiarrhythmic drugs (AADs) remain the "first-line" therapy, these medications have only modest efficacy at maintaining sinus rhythm.[21, 22] Moreover, these agents are associated with significant non-cardiac side-effects, as well as the potential for proarrhythmia (i.e.,

propensity towards malignant ventricular arrhythmia).[23, 24] Owing to the inadequacies of AAD therapy, catheter ablation has emerged as an important non-pharmacological treatment option for maintaining sinus rhythm.[25-32] Over the past 10-15 years, large-scale observational studies, as well as randomized controlled trials have demonstrated that catheter ablation, which is centered on electrical isolation of triggering foci within the pulmonary veins (PVI) through circumferential lesions around PV ostia, is superior to AAD therapy in maintaining sinus rhythm (66-89% vs. 9-58%) and is associated with improved quality of life.[33-42] In addition, catheter ablation has been shown to be superior to AADs for the improvement of symptoms, exercise capacity, and quality of life in patients with paroxysmal AF.[2-4, 36] One small study found catheter ablation to improve mortality (HR 0.46; 95% CI 0.31-0.68; $p < 0.001$), heart failure and stroke related morbidity (HR 0.45; 95% CI 0.31-0.64; $p < 0.001$), as well as quality of life when compared to AAD therapy.[26] In addition to the morbidity benefits associated with catheter ablation, the health care associated cost of catheter ablation is significantly lower than that observed with AAD therapy. An analysis of the Canadian Registry of AF (CARAF) estimated an annual cost associated with AF ablation of $1,597-2,132, which was lower than that observed with medical therapy ($4,176-5,060/year).[43] Thus, for many highly symptomatic patients who cannot be pharmacologically controlled, catheter ablation offers a promising alternative. Current guidelines from the CCS and the HRS/EHRA/ECAS recommend catheter ablation for patients with symptomatic AF refractory to one or more AADs.[44, 45] These guidelines recommend that ablation strategies targeting PVs are the cornerstone of this intervention.[44]

Catheter Ablation

The basis of successful catheter ablation is the production of permanent transmural myocardial lesions that block the propagation of AF wavefronts from a rapidly firing triggering source (usually the PVs) and/or modification of the arrhythmogenic substrate responsible for re-entry.[46, 47] These lesions can be produced by percutaneous catheter-based thermo-coagulation using radiofrequency (RF) energy, or alternatively by thermo-cooling (freezing) with a cryoballoon catheter. While RF catheter ablation for AF has been the method used for many years, it was designed for creating single focal lesion. Despite its prominence in the field of electrophysiology, RF energy has several inherent disadvantages. In the last decade, the development of cryothermal ablation using a cryoballoon catheter offers the ability to achieve circumferential lesion creation around the outside of the PV, and is designed specifically for PVI.[5] To date over 35,000 cryoballoon ablation procedures have been performed worldwide. In a recent meta-analysis of the 23 studies that have reported the outcomes of cryoballoon ablation, we reported that cryoballoon ablation resulted in a high acute procedural success rate (>98% of patients achieving complete PVI) and 1-year freedom from recurrent AF (single cryoballoon ablation procedure off anti-arrhythmic drugs (AAD) 1-year success of 73% [5] This is superior to RF catheter ablation (50% to 64% reported in the meta-analysis by Calkins et al. and 40% at 1 year in the prospective cohort study of Weerasooriya et al.)[48,49]

Cryothermal ablation

Cryothermy offers several advantages when compared to RF energy: 1) Freeze-mediated catheter adhesion results in increased catheter stability, which is particularly advantageous when ablating technically challenging regions such as the ridge between left-sided PVs and the left atrial appendage. Moreover, the increased stability could be expected to result in less collateral damage to nearby structures, such as the PVs or esophagus. 2) Cryoablation results in the creation of well-demarcated homogeneous lesions that are less arrhythmogenic than the ragged indistinct lesions associated with RF ablation. As such, it would be expected that cryoablation decrease the incidence of macro re-entrant left atrial tachycardia, which is observed in 20% of cases post-RFCA. 3) Mature lesions resulting from cryoablation demonstrate preservation of tissue ultrastructural integrity. In the immediate term, preservation of the connective tissue matrix should result in a lower risk of myocardial perforation and esophageal injury. In the longer term, given the minimal tissue contraction observed with lesion healing, it can be expected that lesions produced with cryoablation should result in a lower incidence of PV stenosis. 4) Lesions produced with the application of cryoenergy results in minimal

endocardial surface disruption and are thus less thrombogenic than those produced with RF energy. Moreover, cryoablation appears to activate platelets and the coagulation cascade to a lesser degree than RF ablation. As such, cryoablation is associated with a lower risk of thromboembolism, and post-procedural ischemic cerebral lesions. 5) In addition to the potential safety advances cryoballoon ablation results in a wide proximal lesion, thus not only isolating the muscular PV sleeves but also extending to an antral circumferential lesion (mean extent of LA ablation 40.2±3.9%).

Lesion Formation with Cryothermal Ablation – The Freeze Thaw Cycle

The mechanisms underlying cryothermal lesion formation can be divided into sequential stages: freeze, thaw, haemorrhage and inflammation, and replacement fibrosis.[50] Most important in determining the extent and permanence of lesion formation are the first two phases, both of which are proportional to the temperature achieved and the time of cooling. During the first phase, progressive hypothermia results in a slowing of cellular metabolism, and the formation of extracellular and intracellular ice crystals.[51] The combination of mechanical and biochemical stress results in cellular protein damage, enzyme system impairment, and cellular shrinkage. Upon completion of the freezing phase, the tissue passively returns to body temperature ("thawing effect"). This second phase results in cellular damage through a combination of: 1) recrystallization and coalescence of intra- and extracellular ice crystals, which further disrupt tissue architecture, [52, 53] and 2), a hyperaemic vascular response characterized by haemorrhage and inflammation.[51, 54, 55] Since the early days of cryosurgery there has been a recognition that repeated freeze–thaw cycles produce faster and more extensive tissue cooling, which extend the effect of the ablation lesion to the outer limit of the frozen volume (i.e. the warmer freezing temperature zone at the periphery of the target tissue). To this end, experimental evidence (largely derived from the cancer literature) confirms the increased destructive effect of the second cycle is substantial (see Table). **Thus, while the freezing phase is associated with the induction of cellular damage, it is the thawing phase that results in the extension and consolidation of tissue destruction resulting in an even more durable, and homogeneous lesion.** [56]

Reference			Cell/tissue	Effect
First author	Refs.	Year		
Gill	[74]	1968	Rat liver	Greater volume frozen
Myers	[140]	1969	Tumors, mice	No recurrence
Neel	[147]	1971	Sarcoma, mice	−60 °C required
Whittaker	[213]	1975	Oral mucosa, hamster	Larger intracellular ice crystals
Gage	[57]	1978	Palate, dog	Increased destruction
Burge	[19]	1984	Cartilage, pig ear	Greater destruction
Rand	[167]	1985	Mammary cancer, mice	−50 °C required
Ravikumar	[168]	1991	Tumor, liver, rat	−35 °C required
Dilley	[43]	1993	Liver, sheep	Moved necrosis close to border
Pogrel	[160]	1996	Skin, rat	Wider destruction; −20 °C lethal
Staren	[194]	1997	Breast cancer, rat	Enhanced destruction
Wooley	[214]	2002	Kidney, dog	Enhanced destruction
Seifert	[185]	2003	Liver, dog	Larger lesion
Kollmar	[108]	2004	Liver, pig	Enhanced destruction

Manipulating the Freeze-Thaw Cycle – Ablation Time

The degree of permanent cellular damage induced with cryothermal ablation is directly related to the relationship between tissue temperature (-10 to -25°C) and the duration of freezing.[50, 53] The optimal duration of freezing, that is, how long the tissue should be kept in the frozen state, is not well established. The current recommendations are for cryoablation dosing at 240 seconds for each application, which is based on studies of an early focal cryocatheter. In these studies it was observed that the effect of a cryoablation lesion reached a plateau of three-minutes after the onset of ablation. Thereafter a "prolongation of exposure time beyond 3 minutes did not result in any further increase in lesion dimension or volume".[57, 58] Since then the cryoablation system has undergone a significant redesign. Specifically, the cryocatheter has evolved from a rigid focal catheter to a semi-compliant balloon, which necessitated a redesign of the cryorefrigerant delivery mechanisms, and the

cryorefrigerant employed has changed from slow-cooling to more efficacious gases (i.e., nitrous oxide). Thus, within a relatively brief time frame, the initial 9-French focal cryoablation catheter with slow cooling and a temperature limit of −50◦C was transformed into the modern 7-French version with rapid cooling and achievable temperatures below −80◦C. However, despite these modifications the optimal duration of cryoablation has not been re-challenged. ***It is possible that the current minimum recommended cryoablation "dose" of four minutes is too long. We propose a modification to the current cryoablation process to allow for the inclusion of a slow thawing phase, as well as a repetition of the freeze–thaw cycle, without significant prolongation in the procedure duration.***

Animal Data Supporting Shorter Freeze Durations

Information regarding the safety and efficacy of shorter cryoablation durations are limited. We recently completed a randomized study examining the immediate and delayed effects of shorter ablation time on PVI efficacy.[59] In our study, thirty-two mongrel dogs underwent cryoballoon ablation with a 23mm cryoballoon catheter. PVI procedures were randomized to a single 2-minute vs. 4-minute cryoballoon application. Animals were survived for 30 days after which histopathologic analysis was performed. Acute PVI was attained in 93.3% of PVs after a single cryoballoon application, p=0.023. Mean time to PV isolation was 22.7±17.1 seconds. Although 4-minute lesions were associated with a thicker neointima than 2-minute lesions (223.8µm vs. 135.6µm; p=0.007), no differences were observed in the rates of procedural PVI, or the achievement of complete circumferentially transmural lesions at 30 days (78% overall; 86.2% for 2-min vs. 70% for 4-min; P=0.285). All of the 6 pulmonary vein strictures were observed in the group randomized to 4-minute lesions (6/30 vs. 0/29 left PVs; p=0.024), Thus, this animal model provides a ***preliminary justification that shorter ablation durations with contemporary cryoballoon ablation systems may be safer, and equally efficacious when compared to longer ablation durations***.

Clinical Data Supporting Shorter Freeze Durations

Clinical Information regarding the safety and efficacy of shorter cryoablation durations are limited to focal (non-balloon) cryocatheters. A recent case series focal cryocatheters based PVI demonstrated that the point-by-point application of 90-120 second cycles was efficacious at achieving PVI.[60] In this series, 212 patients with symptomatic AF underwent point-by-point circumferential pulmonary venous cryoablation using the 8mm Freezor MAX focal cryocatheters. Acute procedural success rates for PVI were 100%, confirmed by Lasso catheter and pacing manoeuvres. No patient experienced procedure related chest pain, complications with pulmonary vein isolation or esophageal injury. Sinus rhythm was maintained in 167 of 212 patients (79%) at 3 months, 66% at 6 months and 62% at 1-year follow-up. Antiarrhythmic medication use decreased from 0.8 to 0.4 per patient at 1 year (P<0.05). Recurrent AF occurred in 46 of 212 patients at 3 months. Repeat AF cryoablation was done on 36 of the 46 patients (78%). Twenty-one of the 36 patients (58%) were found to have confirmed pulmonary vein isolation. Fifteen of the 36 patients (42%) were found to have reconnection of the pulmonary veins and required 32±4 minutes of repeat cryoablation for repeat isolation. Based on the results of this study the authors suggested that the dosing of cryoablation for ***90-120 seconds produces adequate acute and chronic block of the pulmonary veins***, with acceptable procedure time and reduced fluoroscopy compared to that of conventional RF ablation. ***It is important to note that the limitations of focal cryocatheters to achieve PVI are extensive. As such, the extension of these results to the more efficacious cryoballoon catheter is impossible, thus highlighting the need for further clinical study.***

Contact Force RF Catheters

Ablation electrode-tissue contact is important determinant of lesion size, and ultimately durability. Conventionally, this has been assessed by the operator using a combination of fluoroscopic imaging of the catheter tip motion, tactile feedback and local electrogram attenuation, as well as impedance reductions during energy delivery. While widely used the accuracy of these surrogate measures is

poor. Contact force sensing is a newly developed technology that allows for the real-time estimation of the contact force between the tip of the catheter and the target myocardium, thus providing the operator with an accurate quantitative assessment of tissue contact. The ThermoCool SmartTouch catheter (Biosense Webster, Diamond Bar, CA) uses the electromagnetic location technology of the CARTO mapping system to detect the movement between the transmitter coil and tip of the electrode. Specifically, a precision spring is mounted within the tip of a 3.5mm externally irrigated RF ablation catheter. This spring allows a small amount of electrode deflection and is connected to a transmitter coil, which emits a location reference signal. Three location sensor coils are also mounted within the shaft of the catheter and detect this signal thereby monitoring small movements of the spring and allowing precise tracking of the catheter tip. These movements are sampled every 50 msec and calibrated to produce a contact force reading (in grams) that is averaged over 1 second.

It is possible that contact force will improve efficacy by minimizing the delivery of ineffective lesions with inadequate contact force, as well as decrease the risk of complications. Recent data suggests that incorporating contact force data into the ablation strategy results in a reduction in procedure time, ablation time and total energy delivery, with a comparable safety profile to that observed with standard irrigated RF. Recent data using the SmartTouch system demonstrated an improved outcome after AF ablation when the procedure was performed within the operators' selected pressures at least 80% of the time (84% one year freedom from AF vs. 62% when performed out of the selected range; P=0.03).

1.2 Study Objective

The primary objective of this study is to verify if repeated freezing cycles (4 minutes or 2 minutes) are more efficacious (i.e., fewer recurrence of AF), and safer, than the established standard radiofrequency catheter ablation.

1.3 Hypothesis to be tested

- In patients with paroxysmal or early persistent atrial fibrillation undergoing pulmonary vein isolation:
  - A PV-ablation strategy utilizing the cryoballoon will result in a superior single-procedure efficacy with comparable or lesser risk of complication when compared to PVI using irrigated radiofrequency energy.
  - A PV-ablation strategy utilizing a "double short" cryoablation lesion duration (2 minute lesions with 2 minute bonus) will result in a superior procedural efficacy with comparable or lesser risk of complication when compared to PVI using a standard cryoablation lesion duration (i.e. 4 minutes), and irrigated radiofrequency energy.

- We hypothesize that cryoballoon ablation, and in particular shorter cryoablation durations (2 + 2 minutes) will result in:
  - A superior acute procedural efficacy with lower risk of spontaneous or adenosine provoked acute procedural pulmonary vein reconnection.
  - A lower incidence of early atrial arrhythmias post ablation (i.e., within 3 months)
  - A superior long-term freedom from any AF/AFL/AT, after the last ablation procedure.
  - A superior long-term freedom from symptomatic AF/AFL/AT, after the last ablation procedure.
  - A lower incidence of peri-procedural complication (including but not limited to tamponade, and thromboembolism)
  - A lower incidence of delayed complications (including but not limited to pulmonary vein stenosis).

2. STUDY PURPOSE

2.1 Study Population

2.1.1 Source and number of patients

A total of 348 patients with symptomatic non-permanent AF refractory to at least one antiarrhythmic drug (AAD) referred for percutaneous catheter ablation will be randomized at approximately 6-10 participating sites in Canada. Enrolment in the trial will be done for patients that have already made the decision to undergo an AF ablation procedure.

2.1.2 Eligibility Criteria

2.1.2.1. Inclusion criteria

- Non-permanent atrial fibrillation documented on a 12 lead ECG, Trans Telephonic Monitoring (TTM) or Holter monitor within the last 24 months:
  - Low Burden Paroxysmal - ≥2 episodes of AF over the past 12 months; Episodes terminate spontaneously within 7 days or via cardioversion within 48 hours of onset.
  - High Burden Paroxysmal – ≥4 episodes of AF over the past 6 months, with ≥ 2 episodes >6 hours in duration; Episodes terminate spontaneously within 7 days or via cardioversion within 48 hours of onset.
  - Early Persistent - ≥2 episodes of AF over the past 12 months; Episodes are successfully terminated via cardioversion within 7 days of onset.
- Age of 18 years or older on the date of consent
- Candidate for ablation based on AF that is symptomatic and refractory (ineffective or intolerant) to at least one class 1 or 3 antiarrhythmic.
- Continuous anticoagulation with warfarin (INR 2-3), low molecular weight heparin, or a direct oral antithrombotic (dabigatran, apixaban, rivaroxaban) for ≥4 weeks prior to the ablation; or a TEE that excludes LA thrombus ≤48 hours before ablation
- Informed Consent Form

2.1.2.2. Exclusion criteria

- Previous left atrial (LA) ablation or LA surgery
- AF due to reversible cause (e.g. hyperthyroidism, cardiothoracic surgery)
- Intracardiac Thrombus
- Pre-existing pulmonary vein stenosis or PV stent
- Pre-existing hemidiaphragmatic paralysis
- Contraindication to anticoagulation or radiocontrast materials
- Anteroposterior LA diameter greater than 5.5 cm by TTE
- Cardiac valve prosthesis
- Clinically significant (moderately-severe, or severe) mitral valve regurgitation or stenosis
- Myocardial infarction, PCI / PTCA, or coronary artery stenting during the 3-month period preceding the consent date
- Cardiac surgery during the three-month interval preceding the consent date

- Significant congenital heart defect (including atrial septal defects or PV abnormalities but not including PFO)
- NYHA class III or IV congestive heart failure
- Left ventricular ejection fraction (LVEF) less than 35%
- Hypertrophic cardiomyopathy (Wall thickness >1.5 cm)
- Significant Chronic Kidney Disease (CKD - eGFR <30 µMol/L)
- Uncontrolled hyperthyroidism
- Cerebral ischemic event (strokes or TIAs) during the six-month interval preceding the consent date
- Pregnancy
- Life expectancy less than one (1) year
- Currently participating or anticipated to participate in any other clinical trial of a drug, device or biologic during the duration of this study
- Unwilling or unable to comply fully with study procedures and follow-up

2.2 Study Design

2.2.1. Trial design

This will be a prospective randomized trial. Patients with symptomatic non-permanent AF refractory to at least one AAD referred for percutaneous catheter ablation and meeting inclusion criteria will be enrolled. Patients will be randomized (blinded to their allocation assignment and stratified by center) in a 1:1:1 allocation ratio to pulmonary vein isolation using:

- **Group 1 / RFCA:** Standard radiofrequency ablation guided by tissue-contact force

- **Group 2 / CRYO 4:** Standard cryoballoon ablation duration (4 minute applications with a 4 minute insurance freeze after isolation is achieved)

- **Group 3 / CRYO 2:** Short cryoballoon ablation (2 minute applications with a 2 minute insurance freeze after isolation is achieved)

2.2.2. Duration of treatment and follow-up period

An implantable loop recorder will be implanted 30 to 90 days prior the index ablation to all patients that have signed the consent form. All patients will be followed for a minimum of 12 months (maximum 36 months) after the index ablation procedure. This duration was based on HRS recommendations for AF ablation clinical trials and the fact that most recurrences transpire during the first year after ablation.[27, 44, 88-90]

2.2.3. Baseline Data Collection

The following baseline data will be collected in all patients before ablation (all of which are a component of standard clinical care):

- Demographic variables (age, sex, race, height, and weight)
- NYHA functional class
- CCS-SAF, EQ-5D and AFEQT (AF symptomatology and quality of life scores)
- Non-cardiac co-morbidities (e.g., diabetes, smoking, hypertension)

- History of other cardiovascular disorders (including stroke)
- Current medications (including anticoagulants)
- Antiarrhythmic treatment history (current and past AADs; including reasons for discontinuation)
- AF history (time since diagnosis, episode durations, emergency visits, hospitalizations, cardioversions)
- Physical examination (heart rate, blood pressure, weight, height)
- Echocardiographic data (including left ventricular ejection fraction, LA diameter, mitral regurgitation/stenosis)
- 12 lead ECG and 24-h Holter monitor
- Cardiac CT or MRI

2.2.4. Implantable loop recorder (ILR)

The patients will undergo a loop recorder implantation (REVEAL LINQ, Medtronic), also called an insertable cardiac monitor (ICM), at a minimum of 30 days (but ideally 90 days) prior to the planned ablation procedure. The use of an implantable loop recorder with an automated AF detection algorithm for the ascertainment of arrhythmia recurrences will enhance participant compliance and provide the optimal the detection of the primary endpoint of asymptomatic or symptomatic arrhythmia recurrence.

The ILR analyses the beat-to-beat variability of cardiac cycles on a 2-minute ECG strip and stores (1) the tracing for visual confirmation, (2) the amount of AF per day (Daily AF Burden, hours in AF in 1 day), and (3) the overall AF Burden (percentage of time in AF). Moreover, the patient has the ability to activate the device manually thus facilitating analysis of heart rhythm during symptomatic events**. In addition, the use of home monitoring (device interrogations) permits intense yet minimally intrusive follow-up.

While from a patient perspective the freedom from symptoms related to AF may be the most important clinical endpoint, the bulk of evidence suggests that presence or absence of symptoms does not affect the prognosis and complications of the AF.[64] Moreover, it is well documented that there is a poor correlation between symptoms and AF burden, thus making the objective evaluation of the effect of a given rhythm control strategy unreliable.[65] In addition, emerging data show that asymptomatic AF occurs frequently after catheter ablation, even in the patients with highly symptomatic AF before the ablation procedure. Lastly, studies have shown that the detection of AF recurrence is proportional to the duration of monitoring, of which implantable loop recorders are the gold standard **(See Figure).**[66] Thus, for a trial designed to evaluate the comparative efficacy of differing ablation strategies the "purest" objective endpoint is that of asymptomatic and symptomatic arrhythmia recurrence as detected by implantable loop monitor.

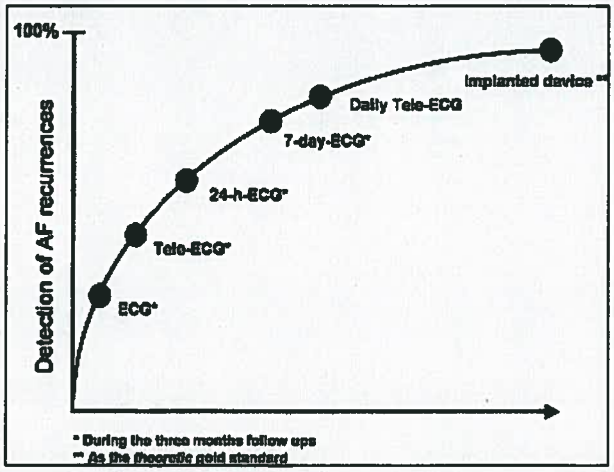

** The loop recorder device should be set to “AF ablation” patient type. This automatically programs the device to AF detection threshold - balanced, Ectopy rejection - Nominal, episode storage threshold – all (Record ECG of 2 minutes). **

2.2.5. Randomization

Patients who meet all the inclusion and exclusion criteria and have had a successful ILR implantation will be randomized and be scheduled for an AF ablation. One hundred and sixteen (116) patients will be randomized to standard radiofrequency, 116 patients will be randomized to standard cryoballoon ablation duration (4 minutes applications) and 116 patients will be randomized to short cryoballoon ablation (2 minutes applications). (For more details, see Statistical considerations)

2.2.6. The Ablation Procedure

2.2.6.1. Pre-Procedure Imaging

The use of adjunctive pre-procedural cardiac magnetic resonance** or computed tomographic imaging, and intracardiac echocardiography or pulmonary venography to define pulmonary venous anatomy and caliber will be based upon physician preference, and regional patterns of practice.[67, 68]

** Reveal LINQ is considered conditionally safe for use in the MRI environment when used under the specified conditions. Patients with a Reveal LINQ that has been implanted for less than 6 weeks are contraindicated for an MRI procedure. The 6-week post-implant waiting period allows sufficient time for implant pocket and wound healing and minimizes the effects of “tugging” on the device caused by the magnetic fields. The MRI environment may interfere with the device’s capability to detect irregular heart rhythms, and therefore, diagnostic information collected during the MRI procedure may be corrupted. Therefore the data recorded and stocked by the device should be downloaded prior the imaging procedure to avoid the loss of the clinical trial data.

2.2.6.2. Para-procedure patient management

- Effective anticoagulation with oral vitamin K antagonists (target INR between 2-3), low molecular weight heparin or dabigatran / apixaban / rivaroxaban for at least one month and/or the exclusion of a LA thrombus by a recent transesophageal echocardiogram (<48 hours; TEE) is mandated prior to ablation.[69]
- As per standard practice, AADs will be discontinued five half-lives before the procedure, except for amiodarone, which will be discontinued 8 weeks prior to ablation.
- AV blocking agents are permitted in highly symptomatic patients.
- Patients will be required to fast the night before the procedure.
- Laboratory analysis: Potassium, Hemoglobin and Creatinine will be measured the day of the ablation procedure.
- Interventions may be performed under conscious sedation or general anesthesia (GA) per local practice.

2.2.6.3. Radiofrequency catheter ablation (RFCA) (Group 1)

Patients randomized to RFCA will have the procedure performed according to standard clinical practice.[28, 69-71]

- A multipolar catheter will be placed in the coronary sinus (CS) via central venous access.
- The LA will be accessed via trans-septal (TS) puncture or patent foramen ovale.
- After TS access is obtained, IV heparin will be administered as a sequential boluses and/or a continuous infusion to maintain an ACT >300 sec.
- A three-dimensional, non-fluoroscopic mapping system (**CARTO3 / Biosense Webster**) will be used for anatomic reconstruction.
- Through one TS access, a circular mapping catheter (decapolar or duo-decapolar) will be advanced into the LA.
- The circular catheter will be sequentially placed within each PV to record baseline electrical activity (PV potentials; PVPs).
- Via a second TS access, an irrigated-tip contact-force ablation catheter **(Thermocool SmartTouch / Biosense Webster)** will be placed in the LA.
- Circumferential ablation lesions will be placed via the ablation catheter 1-2 cm from the PV ostia to electrically isolate the PV, as per standard practice.[44]
  - RF energy will be delivered with an irrigated-tip catheter at 20-35 Watts to a maximum temperature of 43°C.
  - Target contact force prior to lesion delivery – 20g (10-40g)
  - Target lesion duration – minimum 400 gs FTI per individual ablation lesion
- Circumferential lesions around the veins will be considered complete when bidirectional conduction block has been demonstrated.
- No prophylactic left atrial linear ablation lesions, or ablation of complex fractionated atrial electrograms (CFAE) will be permitted in addition to PV isolation.
- Cavotricuspid isthmus ablation in the event of documented right atrial flutter is permitted (irrigated RF or cryoablation is permitted).

2.2.6.4. Cryoballoon Ablation (CBA) (Group 2 and Group 3)

Patients randomized to cryoballoon (CB) ablation will have the procedure performed according to standard clinical practice.

- A multipolar catheter will be placed in the coronary sinus (CS) via central venous access.
- The LA will be accessed via TS puncture or patent foramen ovale.
- After TS access is obtained, IV heparin will be administered as a sequential boluses and/or a continuous infusion to maintain an ACT >300 sec.
- Thereafter the TS sheath will be exchanged with a steerable 15 Fr sheath (**Flexcath / Medtronic**).
- Before introducing the balloon catheter (**Arctic Front / Medtronic)** in the sheath a 15 or 20 mm diameter small-diameter circular mapping catheter (CMC) will be inserted in the central lumen of the CB.
- The 23 or 28 mm CB will be advanced through the steerable sheath into the LA with the CMC used as a guidewire.
  - While the use of the larger (28-mm) CB is preferred, the 23-mm cryoballoon may be used based on physician judgment and assessment of LA and PV anatomy on the pre-procedural imaging (CT scan, MRI) or intra-procedural pulmonary venography.
  - The 23-mm CB will be limited to PV diameters <20 mm.[72-74]
- Before ablation, the CMC will be positioned in the venous ostium to record baseline electrical activity.
- The CMC will then be advanced more distally for support.

- The CB will be positioned in the venous ostium and the degree of occlusion will be tested through the injection of 1:1 diluted contrast material.
- Vessel occlusion will be evaluated according to a semi-quantitative grading:
  - Grade 1 – negligible occlusion with immediate rapid outflow from the PV
  - Grade 2 – mild backflow into the atrium
  - Grade 3 – minimal backflow into the atrium
  - Grade 4 – total contrast retention with no backflow into the atrium
- While every effort should be made to achieve optimal (grade 4) occlusion prior to the initiation of ablation, it should be noted that a small degree of localized leak or delayed emptying of contrast (grade 3 occlusion) may be acceptable, since the onset of cryoablation is associated with balloon expansion, which may improve the seal.
- Prior to ablation of right-sided PVs, a 5-Fr deflectable or non-deflectable catheter will be placed in the superior vena cava cranial to the right superior PV in order to pace the right phrenic nerve (10-20 mA at 1.0-2.0 msec pulse width at a cycle length of 1000 msec).
  - Ablation will be immediately terminated upon any perceived reduction in the strength of diaphragmatic contraction or a 30% reduction in the diaphragmatic compound motor action potential (CMAP) amplitude as measured via diaphragmatic electromyography.
  - Of note, if the procedure is performed under general anesthesia, paralytic agents will be discontinued at least 30 minutes prior to plan phrenic nerve pacing.

a) **Patients randomized to Standard Cryoballoon Ablation (GROUP 2 / CRYO 4):** Patients randomized to the standard cryoballoon ablation group will undergo cryoablation with target duration of 4 minutes. Once PVI is achieved a single "bonus" application of 4 minutes will be delivered after the rewarming phase (to +20°C).

b) **Patients randomized to Short Cryoballoon Ablation (GROUP 3 / CRYO 2):** Patients randomized to the short cryoballoon ablation group will undergo cryoablation with target duration of 2 minutes. Once PVI is achieved a single "bonus" application of 2 minutes will be delivered after the rewarming phase (to +20°C).

Ineffectual lesions

Lesions that fail to isolate the vein (if real-time PV potential monitoring is feasible) or achieve a temperature colder than 35°C after 60 seconds of ablation should be considered ineffectual and be terminated (excluding common ostia). Thereafter the balloon and/or guidewire should be repositioned and a new lesion delivered.

Inability to Isolate

Should the operator fail to isolate the PV (excluding common ostia) after a minimum of 3 attempted cryoballoon applications then focal ablation with the 8mm cryocatheter (Freezor Max) targeted to sites of LA-PV breakthrough would be permitted at operator discretion.

- No prophylactic left atrial linear ablation lesions, or ablation of complex fractionated atrial electrograms (CFAE) will be permitted in addition to PV isolation.

- Cavotricuspid isthmus ablation in the event of documented right atrial flutter is permitted with either irrigated RF or cryoablation.

2.2.6.5. Procedural Success

1. In accordance with the 2012 HRS/EHRA/ECAS consensus document the ablation procedure will be considered successful when PV isolation, as confirmed by bidirectional conduction block between PV and LA, has been achieved.[44]
   i. Entrance Block – the stable absence of conduction into the PV from the LA
   ii. Exit block – the stable absence of conduction from the PV into the LA (either spontaneous or during pacing from the circular mapping catheter positioned at the ostium of the PV).

2. As per standard practice, <u>following isolation of all PVs</u> (i.e., at the end of the last ablation lesion) an observation period of 20 minutes will be used to assess spontaneous recovery of conduction, which if present will undergo further targeted ablation.[44]
   i. In contrast to the relatively high rate of procedural reconnection observed in studies of RFCA (up to 50% in some series), the rate of acute reconnection post CBA appears to be low.[79-85] In studies employing waiting periods of up to 60 minutes, the pooled rate of acute recurrence among the 749 treated PVs was <1%.[81-85]

3. Following the waiting period dormant conduction will be assessed in each PV by intravenous injection of 12 mg or more of adenosine to obtain at least one blocked P wave or a pause ≥3 seconds. Dormant conduction will be defined by reappearance of PV conduction demonstrated by PV activity recorded on an appropriately positioned circular catheter for ≥1 beat.
   i. If there is no dormant conduction in any PV then the procedure will be considered complete
   ii. If dormant conduction is elicited, the patient will undergo additional targeted ablation until dormant conduction is abolished as assessed by injections of 12 mg or more of adenosine to obtain at least one blocked P wave or a pause ≥3 seconds.

4. No induction testing (using burst pacing or isoproterenol infusion) will be permitted in addition to PV isolation.

2.2.6.6. Post procedural Care

- Barring complications, patients will be discharged within 24 hours of the index ablation procedure.

- Post-procedure evaluation at the end of hospital stay will permit the assessment of the nature and severity of all adverse events occurring during the immediate post-procedural phase.

- Patients will be contacted by phone one week after the index ablation. This phone contact will permit to review the general condition of the patient and will do the follow up the adverse events.

2.2.7. Follow-up period

2.2.7.1. Post ablation blanking period

In accordance with HRS/EHRA/ECAS recommendations for reporting outcomes in AF ablation trials a blanking period of 3 months is incorporated.[44] The rationale for the incorporation of a post-procedure blanking period is based on the observation that early recurrences of arrhythmias are common during the initial post AF ablation period, and is predicated on the assumption that not all early recurrences of atrial tachyarrhythmias (AF/AFL/AT) will lead to later recurrences and, as such, does not necessarily represent treatment failure.[91-94] Despite this relatively high incidence of early recurrence, a substantial proportion of patients with early recurrence appear to experience a favorable arrhythmia-free longer-term clinical course, suggesting that early recurrence may be a transient phenomenon in some.[95]

2.2.7.2. Anticoagulation

All patients will remain anticoagulated with an oral anticoagulant (dose-adjusted warfarin to a target INR of 2-3 or direct oral antithrombotic) for ≥3 months post-ablation.

- Discontinuation of oral anticoagulation during the study period is strongly discouraged (except for patients with a CHADSVaSC score of <2, in whom aspirin alone may be considered at treating physician discretion).

2.2.7.3. Antiarrhythmic Drugs

Antiarrhythmic drugs (which were discontinued five (5) half-lives prior to ablation) may be restarted post ablation at operator discretion (except amiodarone). However, if utilized in the post-ablation period the AADs must be discontinued <u>within five (5) half-lives of the end of the 3-month blanking period</u> in accordance with HRS guidelines.[44]

2.2.7.4. Arrhythmia recurrence

Arrhythmia recurrence during the first 3 months post ablation may be treated with cardioversion and/or AADs (except amiodarone).

- Where possible, repeat ablation procedures will be deferred until after the 3-month blanking period due to the potential for delayed cure (as per standard practice and in accordance with HRS/ECAS/EHRA recommendations).[44]

2.2.7.5. Follow-up visits

Scheduled visits will occur at 3, 6, and 12 months from the first ablation procedure (within 2 weeks) for evaluation of the primary and secondary outcomes.

- A 24-hour Holter and 12-lead ECG will be performed at 3, 6, and 12 months.
- For patients unable to participate in remote monitoring the follow-up schedule will be modified to include a 9 month visit. The implanted loop recorder will be interrogated at each follow-up visit.

Table in Appendix A details all visits and procedures.

3. STUDY METHODOLOGY

3.1. Primary and secondary outcomes measures

3.1.1. Primary endpoint

- Time to first recurrence of symptomatic or asymptomatic AF, atrial flutter, or atrial tachycardia (AF/AFL/AT) documented by 12-lead ECG, surface ECG rhythm strips, ambulatory ECG monitor, or on implantable loop recorder and lasting 120 seconds or longer as adjudicated by a blinded group of investigators between days 91 and 365 post ablation, or a repeat ablation procedure between days 0 and 365 post ablation.

3.1.2 Secondary Endpoints

- Time to first recurrence of symptomatic** documented AF/AFL/AT between days 91 and 365 after ablation or a repeat ablation procedure between days 0 and 365 post ablation.
- Total arrhythmia burden (daily AF burden - hours/day; overall AF burden - % time in AF)
- Proportion of patients experiencing an acute or adenosine provoking PV reconnection during the index ablation procedure.
- Proportion of patients who needed repeat ablation procedure because of documented recurrence of symptomatic AF/AFL/AT
- Proportion of patients who needed antiarrhythmic drug because of documented recurrence of symptomatic AF/AFL/AT.
- Proportion of patients with AF/AFL/AT during the first 90 days post ablation
- Emergency visit or hospitalization >24h in a health-care facility
- Major complications including death, stroke, TIA, Myocardial Infarction or systemic thromboembolism, PV stenosis, phrenic nerve palsy, pericarditis, pericardial effusion, cardiac perforation or tamponade, hematoma, AV fistula, pseudoaneurysm, esophageal injury and atrio-esophageal fistulae (both individually and as a composite endpoint). Standard definitions as per the 2012 HRS/EHRA/ECAS recommendations are employed. Acute peri-procedural complications will be defined as occurring within 30 days of ablation, with delayed complications occurring 31-365 days after ablation.
- Overall and disease specific quality of life.
- Single and multiple procedure success (freedom from **symptomatic or asymptomatic** electrocardiographically documented AF/AFL/AT) after the first and last ablation procedure respectively
- Single and multiple procedure success (freedom from **symptomatic** electrocardiographically documented AF/AFL/AT) after the first and last ablation procedure respectively

** To date ablation has not been shown to improve mortality endpoints, and is largely performed for quality of life reasons. From a patient perspective, a reduction in symptomatic AF episodes is considered a procedural success, even though asymptomatic AF may persist. To document the effect of the ablation strategies on symptomatic AF, patients will be asked to activate the device manually to facilitate analysis of heart rhythm during symptomatic events and record the exact onset and offset of symptoms. Symptoms may include: palpitations, irregular pulse (rapid, racing, pounding, fluttering), dizziness, weakness, chest discomfort, and breathlessness.

3.2. Safety Monitoring

3.2.1. Description of risks to the safety of participants involved in the trial

Enrolment in the proposed trial is predicated on assumption that patients have already made the decision to undergo an AF ablation procedure. As such, independent of trial enrolment they would have accepted the risks of AF ablation. The most recent estimates of the overall procedural risks of catheter ablation are outlined in the table (RFCA = radiofrequency catheter ablation; CBA = cryoballoon ablation). Of note, cryoballoon ablation is associated with lower rates of thromboembolism (stroke/TIA), cardiac tamponade, pulmonary vein stenosis, and atrio-esophageal fistula compared to standard RF techniques.

Table – Risk of AF Ablation Procedures	RFCA	CBA
Stroke or TIA	0.3-0.9%	0.32%
Ischemic cerebral lesions post ablation	7.4-8.3%	4.3-5.6%
Cardiac Tamponade	0.8-1.3%	0.6%
PV stenosis		
- Radiographic (routine screening)	1.6%	0.9%
- Symptomatic or requiring intervention	0.1-0.29%	0.17%
Atrio-esophageal fistula	0.04-0.2%	<0.0001
Persistent phrenic nerve palsy	0.17%	0.37%

While the proposed trial should not offer additional risk over and above that currently expected with standard AF ablation, **the proposed study has the potential to reduce the rates of complication.** Specifically, the majority of complications of AF ablation are related to the prolonged left atrial time, the larger surface area of tissue ablated, the cumulative energy delivery, and the close location of structures susceptible to collateral injury. By reducing the lesion time to two minutes instead of four there exists the potential to reduce left atrial time as well as the cumulative energy delivered. Moreover, based on our preclinical animal study we observed lower degrees of neointimal proliferation (a marker of endothelial damage) in animals randomized to 2-minute cryoapplications. Therefore, there may be the potential for reduced complications with the double-short group when compared to the 4 minutes applications.

3.2.2. Adverse events

An adverse event is defined as any untoward medical occurrence in a subject participating in a clinical study, whether or not there is a causal relationship with the ablation procedure occurring or detected from the participant's signature of information and consent form. Adverse events occurring during the study will be documented in dedicated "Adverse event Forms". Those events will be classified by the investigators according to their seriousness, and whether they are study / procedure related.

Information regarding AEs will be collected from the ILR implantation through and including the last visit. Any AEs prior to the implantation will be recorded on the medical history form. Each patient will be observed and queried in a non-specific fashion at each visit during the study for any new or continuing symptoms since the previous visit.

3.2.3. Serious adverse events

Serious adverse events will be defined as:

- Death,
- Life-threatening event, NOTE: the term "Life-Threatening" refers to an event in which the patient was at immediate risk of death at the time of the event; it does not refer to an event which could hypothetically have caused a death had it been more severe.
- Events causing functional disability,
- Event requiring or prolonging hospitalization,
- or any other event considered serious by the investigator.

Any serious adverse event should be documented and reported to the coordinating center as soon as possible, but within 48 hours after being aware.

3.2.4. Major complications associated with AF ablation

Major complications associated with AF ablation procedure are one of the secondary endpoints. These events will be reviewed and confirmed by an independent events committee. Major complications associated with AF ablation are, but not limited to:

- Death
- Stroke, TIA, Myocardial infarction (MI)
- Systemic thromboembolism,
- Pulmonary vein stenosis
- Phrenic nerve palsy,
- Cardiac perforation or Cardiac Tamponade
- Pericarditis
- Pericardial effusion
- Atrio-esophageal fistulae
- Esophageal injury
- Hematoma
- AV fistula
- Pseudoaneurysm

* These events will be attributed to the index ablation if:
  - Embolic events occurring within 48 hours of the ablation procedure
  - Pericardial effusion occurring within one-month post procedure
  - Occurrence of pulmonary vein stenosis at any time after ablation

These major complications are defined as per the 2012 HRS/EHRA/ECAS recommendations and are listed in Appendix B.

3.3. Study termination

If a patient wishes to withdraw from the study at any time, s/he would be able to do so without having to justify it and without affecting her/his relationship with the investigator. Also, an investigator may withdraw a patient from the study at any time if s/he thinks it is in the patient's best interest. In any case, the investigator will be requested to complete a "Termination" form for any patient that completes the study or leaves the study, whatever the reasons.

Any patient who refuses to undergo follow-up as scheduled will be considered to have withdrawn from the study. The patient's future management will not be changed by a decision, voluntary or otherwise, to withdraw from the study. The study will be terminated according to locally applicable regulations.

3.4. Informed consent

The investigating physician will inform each patient of the objectives, constraints and anticipated risks during the study. Each patient will be provided with a pamphlet containing all relevant information and a consent form and will have had satisfactory responses to all questions at any time before, during or after inclusion in the study. If the patient agrees to participate in the study, he will sign the consent form. One copy of this consent form will remain with the patient; a second copy will be retained at the investigation site.

3.5. Data Collection

3.5.1. Implantable Loop Recorder

The ILR (REVEAL LINQ, Medtronic) will be implanted at least 30 days prior the index ablation. The REVEAL LINQ will be programmed for a specific AF detection algorithm. It analyses the beat-to-beat variability of cardiac cycles on a 2-minute ECG strip and stores the tracing for visual confirmation, the amount of AF per day (Daily AF Burden, hours in AF in 1 day), and the overall AF Burden (percentage of time in AF). The patient is able to activate the device manually thus facilitating analysis of heart rhythm during symptomatic events. In addition, home monitoring (device interrogations and data downloading into the Medtronic CareLink system) is possible thus permitting fairly intense, yet minimally intrusive follow-up.

3.5.2. Case report form

Electronic case report forms (eCRF) are to be completed as instructed for all patients.

4. STATISTICAL CONSIDERATIONS

4.1. Protection from bias

Random allocation to the three ablation strategies will minimize known and unknown systematic differences between study groups, with standardization of inclusion criteria, definitions, and outcomes assessment. Concealed allocation will minimize selection bias and patient blinding will minimize performance bias. Since the treating physician is not blinded, in particular, performance bias is a concern. One option considered was to design a double-blind study by having the treating physician being different then the ablation physician. However, the ethics and logistics of having dual physicians in this instance mitigates against this approach, since the ablation physician is in the best position for making treatment decisions that in the best interest of the patient. The alternative approach is to ensure that the primary outcome is beyond the influence of the physician that would arise through performance bias (i.e. systematic differences between therapy groups in the care provided or exposure to factors other than the therapies of interest). In this regard, AF recurrence will be determined predominantly based on the implantable loop recorder (as well as other non-invasive electrocardiographic techniques) interpreted by independent blinded assessors and adjudicated by a

blinded committee, which is not affected by any potential bias on the part of an unblinded treating physician. An independent adjudicating committee blinded to treatment allocation will classify all outcomes. The use of implantable loop recorders will optimize the objectification of asymptomatic AF episodes, which commonly occur during the follow-up of patients after AF ablation procedures.[61-63]

4.2. Statistical Power and Sample size considerations

The sample size is based on the primary endpoint and on the two main comparisons of interest: CRYO 4 vs. RFCA and CRYO 2 vs. RFCA. The overall event-free proportion at one year is expected to be around 65%. With a sample size of 111 per group and a two-sided 0.025 significance level (to account for the two main comparisons), the study will have 80% power (using a log rank test) to detect a difference of 20% between RFCA and any one of the two cryoballoon groups. In terms of events, the trial will have 80% power if it continues until 71 events occurred in the two groups that are compared. Factoring in a 4% loss to follow-up rate, 116 patients per group should be randomized, for a total of 348 in the study.

Number of patients required in each group according to various hypothesized effects (alpha=0.025)

Study Power	Magnitude of reduction in recurrences				
	10%	15%	20% (55% - 75%)	23%	25%
85%	487	219	126	96	82
80%	431	194	***111***	85	73

Review of the cryoballoon ablation literature indicates this would be one of the largest cryoballoon ablation trials to date. Similarly designed ablation studies have consistently enrolled between 50 and 140 patients.

4.3. Recruitment rate

The study will be conducted at 6-10 clinical centers in Canada. All investigators are from high-volume AF-ablation referral centers. Projecting a conservative enrolment of 2-3 patients/center/month (which represents <30% of the 2009 AF ablation volumes of the participating centers), and considering the regulatory phase delay for site activation, approximately 18 months would be required to enroll 348 patients.

4.4. Proposed type of analyses

The analysis of the primary and secondary endpoints will be based on the intention-to-treat principle according to the initial allocated strategy. An exploratory analysis of the primary endpoint will exclude patients with major deviations from the protocol.

The primary endpoint is the time to first recurrence of symptomatic or asymptomatic documented AF/AFL/AT between days 91 and 365 post ablation, or repeat ablation procedure between days 0 and 365 post ablation. Survival curves will be estimated by the Kaplan-Meier method and compared by the log rank test. The two main comparisons will be CRYO 4 vs RFCA and CRYO 2 vs RFCA and will be conducted at 0.025 significance level. These will be considered as the primary analysis of the study. The other comparison, CRYO 4 vs CRYO 2 will be considered as a secondary.

A Cox proportional hazards model will also be used to test the consistency of the group effect while accounting for clinically important baseline characteristics. The adjusted hazard ratios will be

presented for the group effect with confidence intervals. Important baseline characteristics will include but are not limited to: site, age, gender, race, weight, LA size, structural heart disease, AF duration, and number of AADs used in the past. The proportional hazard assumption will be assessed by visual inspection of the log-negative-log plot and through a formal test of the interaction term "group x time" at $\alpha=0.05$. Should this assumption fail, a stratified Cox model will be fitted in order to correct for non-proportional hazards if possible or, if ineffective, time-dependent variables will be introduced. Should these corrective techniques fail, logistic regression will be used instead. Secondary endpoints expressed as time to event will be analyzed similarly using Kaplan-Meier survival curves and a log rank test. For all dichotomous qualitative variables, Chi-Square tests will be performed to assess group differences. Continuous variables, such as arrhythmia burden, will be analyzed using an analysis of variance (ANOVA). If the data are not normally distributed, then the non-parametric Wilcoxon Signed Rank test will be used. Health-related QoL scores will be compared by analysis of covariance, adjusting for baseline values to reduce the error mean squares. In the event of missing data, a multiple imputation approach using SAS procedures PROC MI and PROC MIANALYZE will be considered, if necessary. All tests will be conducted at an alpha level of 0.05 with the exception of the two main comparisons that will be conducted at an alpha level of 0.025. Similarly, hazard ratios for these two comparisons will be presented with 97.5% confidence intervals.

4.5. Proposed frequency of analyses

One analysis will be performed at the end of the study. An interim analysis is not deemed necessary because of the relatively short study duration (1-year).

4.6. Planned subgroup analyses

Adequate sample sizes permitting, subgroup analyses to investigate heterogeneity in overall effects for the primary endpoint will be performed using Cox proportional hazards models including terms for the factor defining the subgroups, the group and the factor-by-group interaction. The factor-by-group interaction will be tested and used to determine the consistency of the group effect across subgroups. Subgroups based on the following variables will be considered: ablation experience (high volume vs. low volume centers as defined by procedure volume above/below the median), LA size (above/below median), AF duration (above/below median), AF subtype (paroxysmal vs. early persistent), PV anatomy (common ostia vs. standard PV pattern).

5. STUDY ORGANIZATION

5.1. Day-to-day trial management

The Montreal Health Innovations Coordinating Center (MHICC) will coordinate the study. The support staff at the MHICC will be composed of a full-time study coordinator, data manager, and biostatistician. The MHICC will run all aspects of clinical data management. Management operations include: coordination of data collection and data integration; data entry; data quality edit checks; management and resolution of data discrepancies; tracking of adverse event information; generating reports for principal and co-applicants, study sites and for committee meetings; database quality control; locking of the clinical data and performing the final analysis of the trial results.

The handling of data including data quality control, will comply with all applicable regulatory guidelines, and MHICC SOPs.

The MHICC will also generate quarterly recruitment progress reports and transmit these reports to all sites. The MHICC will work with the study investigators to generate and transmit a final progress report to every local center to give to their respective governing Ethics Committees.

5.2. Study Investigators

Learning Curve: All operators are from high-volume Canadian centers (those that have performed >100 procedures) and meet current competency requirements outlined in CHRS and HRS competency documents.[75, 76] Specifically, with RFCA a minimum of 50 procedures is required.[77] For CBA, there are no recommended competency documents. However, evidence suggests that the performance of 12–23 cryoablation cases results in improved single-procedure success reduced the total procedure time, and reduced fluoroscopy time.[38, 78] Therefore in order to participate in the study operators will be required to have performed at least 20 CBA procedures and 50 RFCA procedures.

5.3. Role of each principal and co-applicant

Dr. Jason Andrade (principal applicant): Dr. Andrade is a cardiac electrophysiologist specialized in complex catheter ablation. He will oversee the development of the trial start-up phase, chair the management team that will monitor and supervise trial progress on a daily basis (recruitment, site issues, data and coordinating center issues), chair the steering committee and be the local PI at VGH. Following analysis of the trial results, he will draft the principal manuscript that will be submitted to the steering committee for review.

Dr. Anthony Tang (co-principal applicant): Dr. Tang is a clinician-scientist and a cardiac electrophysiologist with specific interest in cardiac rhythm device management and complex catheter ablation. He is conducting and has conducted international, multicenter randomized controlled trials.

Dr. George Wells (co-applicant): Dr. Wells is a Professor of the Department of Epidemiology and Community Medicine at the University of Ottawa, Senior Scientist at the Ottawa Health Research Institute and Director, Cardiovascular Research Methods Centre at the University of Ottawa Heart Institute. Dr. Wells' interests are in the design and analysis of multicenter clinical trials, statistical methodology related to disease processes and health care delivery, systematic reviews and meta-analysis and the development and assessment of decision support technologies for patients and clinicians. He has been on the executive and steering committees of national and international research programs, external safety and efficacy monitoring committees, scientific grant review committees, editorial committees and scientific advisory committees.

Dr. Marc Dubuc (co-applicant): Dr. Dubuc is a cardiac electrophysiologist with specific interest in complex catheter ablation, and in particular cardiac cryoablation.

5.4. Steering Committee

The Steering Committee will be responsible for the design, execution, analysis, and reporting of the study. The Steering Committee will be responsible for maintaining the scientific integrity of the study, supervising study progress, taking decisions following notifications by the DSMB, reviewing all trial results and, planning and implementing the publications, abstracts and presentations. The Committee will meet once a year, with teleconferencing as necessary to supervise the overall conduct of the study. The Steering Committee will be composed of 9 individuals.

5.5. Clinical Events Committee (CEC)

The clinical events committee will review and adjudicate all primary AF outcomes and serious adverse events (SAE). This committee will be blinded to the treatment arm (AF ablation method) the patient was randomized when reviewing the study outcome events.

6. POST TRIAL KNOWLEDGE DISSEMINATION

6.1. Anticipated results and their implications

The outcome of the proposed has the potential to fundamentally alter the way that cardiac cryoablation is performed. Should a “double-short” freeze-thaw-freeze cycle be superior to the standard 4-minute long cycle then a systematic modification of the cardiac cryoablation technique would be expected to translate into more effective health services and improved health for Canadians suffering from cardiac arrhythmias (including but not limited to AF).

6.2. Plan for Knowledge Dissemination

The information derived herein (whether positive, or negative) will be disseminated through traditional channels such as conference presentations, and publications in peer-reviewed journals. Should the findings be positive then the information will be presented directly to stakeholders (i.e. Medtronic CryoCath), who are able to more easily disseminate the uptake of knowledge through formal educational session, and modifications to the programming of the cryoconsole itself.

7. GLOSSARY

AAD	-	Antiarrhythmic drug
ACT	-	Activate clotting time
AF	-	Atrial fibrillation
AFL	-	Atrial Flutter
AT	-	Left Atrial Tachycardia
CB	-	Cryoballoon
CBA	-	Cryoballoon Ablation
CFAE	-	Complex fractionated atrial electrograms
CKD	-	Chronic kidney disease
CMAP	-	Compound motor action potential
CMC	-	Circular mapping catheter
CS	-	Coronary sinus
GA	-	General anesthesia
ILR	-	Implantable loop recorder
LA	-	Left atria
LEVF	-	left ventricular ejection fraction
PCI	-	Percutaneous coronary intervention
PFO	-	Patient Foramen ovale
PTCA	-	Percutaneous transluminal coronary angioplasty
PV	-	Pulmonary vein
PVP	-	Pulmonary vein potential
RF	-	Radiofrequency
RFCA	-	Radiofrequency catheter ablation
TIA	-	Transient ischemic attack
TEE	-	Transesophageal echocardiogram
TS	-	Trans-septal
TTM	-	Trans Telephonic Monitoring

8. REFERENCES

[1] Wolf PA, Mitchell JB, Baker CS, Kannel WB, D'Agostino RB. Impact of atrial fibrillation on mortality, stroke, and medical costs. *Arch Intern Med* 1998; **158**: 229-34.

[2] Bunch TJ, Crandall BG, Weiss JP, May HT, Bair TL, Osborn JS, et al. Patients treated with catheter ablation for atrial fibrillation have long-term rates of death, stroke, and dementia similar to patients without atrial fibrillation. *J Cardiovasc Electrophysiol* 2011; **22**: 839-45.

[3] Piccini JP, Lopes RD, Kong MH, Hasselblad V, Jackson K, Al-Khatib SM. Pulmonary vein isolation for the maintenance of sinus rhythm in patients with atrial fibrillation: a meta-analysis of randomized, controlled trials. *Circ Arrhythm Electrophysiol* 2009; **2**: 626-33.

[4] Wokhlu A, Monahan KH, Hodge DO, Asirvatham SJ, Friedman PA, Munger TM, et al. Long-term quality of life after ablation of atrial fibrillation the impact of recurrence, symptom relief, and placebo effect. *J Am Coll Cardiol* 2010; **55**: 2308-16.

[5] Andrade JG, Khairy P, Guerra PG, Deyell MW, Rivard L, Macle L, et al. Efficacy and safety of cryoballoon ablation for atrial fibrillation: A systematic review of published studies. *Heart Rhythm* 2011; **8**: 1444-51.

[6] Fuster V, Ryden LE, Cannom DS, Crijns HJ, Curtis AB, Ellenbogen KA, et al. ACC/AHA/ESC 2006 Guidelines for the Management of Patients with Atrial Fibrillation: a report of the American College of Cardiology/American Heart Association Task Force on Practice Guidelines and the European Society of Cardiology Committee for Practice Guidelines (Writing Committee to Revise the 2001 Guidelines for the Management of Patients With Atrial Fibrillation): developed in collaboration with the European Heart Rhythm Association and the Heart Rhythm Society. *Circulation* 2006; **114**: e257-354.

[7] Feinberg WM, Blackshear JL, Laupacis A, Kronmal R, Hart RG. Prevalence, age distribution, and gender of patients with atrial fibrillation. Analysis and implications. *Arch Intern Med* 1995; **155**: 469-73.

[8] Go AS, Hylek EM, Phillips KA, Chang Y, Henault LE, Selby JV, et al. Prevalence of diagnosed atrial fibrillation in adults: national implications for rhythm management and stroke prevention: the AnTicoagulation and Risk Factors in Atrial Fibrillation (ATRIA) Study. *JAMA* 2001; **285**: 2370-5.

[9] Benjamin EJ, Wolf PA, D'Agostino RB, Silbershatz H, Kannel WB, Levy D. Impact of atrial fibrillation on the risk of death: the Framingham Heart Study. *Circulation* 1998; **98**: 946-52.

[10] Nieuwlaat R, Prins MH, Le Heuzey JY, Vardas PE, Aliot E, Santini M, et al. Prognosis, disease progression, and treatment of atrial fibrillation patients during 1 year: follow-up of the Euro Heart Survey on atrial fibrillation. *Eur Heart J* 2008; **29**: 1181-9.

[11] Miyasaka Y, Barnes ME, Bailey KR, Cha SS, Gersh BJ, Seward JB, et al. Mortality trends in patients diagnosed with first atrial fibrillation: a 21-year community-based study. *J Am Coll Cardiol* 2007; **49**: 986-92.

[12] Krahn AD, Manfreda J, Tate RB, Mathewson FA, Cuddy TE. The natural history of atrial fibrillation: incidence, risk factors, and prognosis in the Manitoba Follow-Up Study. *Am J Med* 1995; **98**: 476-84.

[13] Flegel KM, Shipley MJ, Rose G. Risk of stroke in non-rheumatic atrial fibrillation. *Lancet* 1987; **1**: 526-9.

[14] Wolf PA, Abbott RD, Kannel WB. Atrial fibrillation as an independent risk factor for stroke: the Framingham Study. *Stroke* 1991; **22**: 983-8.

[15] Risk factors for stroke and efficacy of antithrombotic therapy in atrial fibrillation. Analysis of pooled data from five randomized controlled trials. *Arch Intern Med* 1994; **154**: 1449-57.

[16] Waktare JE, Camm AJ. Acute treatment of atrial fibrillation: why and when to maintain sinus rhythm. *Am J Cardiol* 1998; **81**: 3C-15C.

[17] Humphries KH, Jackevicius C, Gong Y, Svensen L, Cox J, Tu JV, et al. Population rates of hospitalization for atrial fibrillation/flutter in Canada. *Can J Cardiol* 2004; **20**: 869-76.

[18] Stewart S, Murphy NF, Walker A, McGuire A, McMurray JJ. Cost of an emerging epidemic: an economic analysis of atrial fibrillation in the UK. *Heart* 2004; **90**: 286-92.
[19] Ringborg A, Nieuwlaat R, Lindgren P, Jonsson B, Fidan D, Maggioni AP, et al. Costs of atrial fibrillation in five European countries: results from the Euro Heart Survey on atrial fibrillation. *Europace* 2008; **10**: 403-11.
[20] Gillis AM, Verma A, Talajic M, Nattel S, Dorian P. Canadian Cardiovascular Society atrial fibrillation guidelines 2010: rate and rhythm management. *Can J Cardiol* 2011; **27**: 47-59.
[21] Roy D, Talajic M, Dorian P, Connolly S, Eisenberg MJ, Green M, et al. Amiodarone to prevent recurrence of atrial fibrillation. Canadian Trial of Atrial Fibrillation Investigators. *N Engl J Med* 2000; **342**: 913-20.
[22] Singh SN, Singh BN, Reda DJ, Fye CL, Ezekowitz MD, Fletcher RD, et al. Comparison of sotalol versus amiodarone in maintaining stability of sinus rhythm in patients with atrial fibrillation (Sotalol-Amiodarone Fibrillation Efficacy Trial [Safe-T]). *Am J Cardiol* 2003; **92**: 468-72.
[23] Roden DM. Mechanisms and management of proarrhythmia. *Am J Cardiol* 1998; **82**: 49I-57I.
[24] Doyle JF, Ho KM. Benefits and risks of long-term amiodarone therapy for persistent atrial fibrillation: a meta-analysis. *Mayo Clin Proc* 2009; **84**: 234-42.
[25] de Waroux JB, Talajic M, Khairy P, Guerra PG, Roy D, Thibault B, et al. Pulmonary vein isolation for the treatment of atrial fibrillation: past, present and future. *Future Cardiol* 2010; **6**: 51-66.
[26] Pappone C, Rosanio S, Augello G, Gallus G, Vicedomini G, Mazzone P, et al. Mortality, morbidity, and quality of life after circumferential pulmonary vein ablation for atrial fibrillation: outcomes from a controlled nonrandomized long-term study. *J Am Coll Cardiol* 2003; **42**: 185-97.
[27] Cheema A, Dong J, Dalal D, Vasamreddy CR, Marine JE, Henrikson CA, et al. Long-term safety and efficacy of circumferential ablation with pulmonary vein isolation. *J Cardiovasc Electrophysiol* 2006; **17**: 1080-5.
[28] Macle L, Jais P, Weerasooriya R, Hocini M, Shah DC, Choi KJ, et al. Irrigated-tip catheter ablation of pulmonary veins for treatment of atrial fibrillation. *J Cardiovasc Electrophysiol* 2002; **13**: 1067-73.
[29] Karch MR, Zrenner B, Deisenhofer I, Schreieck J, Ndrepepa G, Dong J, et al. Freedom from atrial tachyarrhythmias after catheter ablation of atrial fibrillation: a randomized comparison between 2 current ablation strategies. *Circulation* 2005; **111**: 2875-80.
[30] Jais P, Hocini M, Hsu LF, Sanders P, Scavee C, Weerasooriya R, et al. Technique and results of linear ablation at the mitral isthmus. *Circulation* 2004; **110**: 2996-3002.
[31] Kottkamp H, Tanner H, Kobza R, Schirdewahn P, Dorszewski A, Gerds-Li JH, et al. Time courses and quantitative analysis of atrial fibrillation episode number and duration after circular plus linear left atrial lesions: trigger elimination or substrate modification: early or delayed cure? *J Am Coll Cardiol* 2004; **44**: 869-77.
[32] Oral H, Knight BP, Tada H, Ozaydin M, Chugh A, Hassan S, et al. Pulmonary vein isolation for paroxysmal and persistent atrial fibrillation. *Circulation* 2002; **105**: 1077-81.
[33] Pappone C, Vicedomini G, Augello G, Manguso F, Saviano M, Baldi M, et al. Radiofrequency catheter ablation and antiarrhythmic drug therapy: a prospective, randomized, 4-year follow-up trial: the APAF study. *Circ Arrhythm Electrophysiol* 2011; **4**: 808-14.
[34] Pappone C, Augello G, Sala S, Gugliotta F, Vicedomini G, Gulletta S, et al. A randomized trial of circumferential pulmonary vein ablation versus antiarrhythmic drug therapy in paroxysmal atrial fibrillation: the APAF Study. *J Am Coll Cardiol* 2006; **48**: 2340-7.
[35] Wazni OM, Marrouche NF, Martin DO, Verma A, Bhargava M, Saliba W, et al. Radiofrequency ablation vs antiarrhythmic drugs as first-line treatment of symptomatic atrial fibrillation: a randomized trial. *JAMA* 2005; **293**: 2634-40.
[36] Jais P, Cauchemez B, Macle L, Daoud E, Khairy P, Subbiah R, et al. Catheter ablation versus antiarrhythmic drugs for atrial fibrillation: the A4 study. *Circulation* 2008; **118**: 2498-505.

[37] Oral H, Pappone C, Chugh A, Good E, Bogun F, Pelosi F, Jr., et al. Circumferential pulmonary-vein ablation for chronic atrial fibrillation. *N Engl J Med* 2006; **354**: 934-41.

[38] Packer DL, Irwin JM, Champagne J, Guerra PG, Dubuc M, Wheelan KR, et al. Cryoballoon ablation of pulmonary veins for paroxysmal atrial fibrillation: first results of the North American Arctic Front STOP-AF pivotal trial. *J Am Coll Cardiol* 2010; **55**: E3015-6.

[39] Krittayaphong R, Raungrattanaamporn O, Bhuripanyo K, Sriratanasathavorn C, Pooranawattanakul S, Punlee K, et al. A randomized clinical trial of the efficacy of radiofrequency catheter ablation and amiodarone in the treatment of symptomatic atrial fibrillation. *Journal of the Medical Association of Thailand = Chotmaihet thangphaet* 2003; **86 Suppl 1**: S8-16.

[40] Noheria A, Kumar A, Wylie JV, Jr., Josephson ME. Catheter ablation vs antiarrhythmic drug therapy for atrial fibrillation: a systematic review. *Arch Intern Med* 2008; **168**: 581-6.

[41] Stabile G, Bertaglia E, Senatore G, De Simone A, Zoppo F, Donnici G, et al. Catheter ablation treatment in patients with drug-refractory atrial fibrillation: a prospective, multi-centre, randomized, controlled study (Catheter Ablation For The Cure Of Atrial Fibrillation Study). *Eur Heart J* 2006; **27**: 216-21.

[42] Wilber DJ, Pappone C, Neuzil P, De Paola A, Marchlinski F, Natale A, et al. Comparison of antiarrhythmic drug therapy and radiofrequency catheter ablation in patients with paroxysmal atrial fibrillation: a randomized controlled trial. *JAMA* 2010; **303**: 333-40.

[43] Khaykin Y, Morillo CA, Skanes AC, McCracken A, Humphries K, Kerr CR. Cost comparison of catheter ablation and medical therapy in atrial fibrillation. *J Cardiovasc Electrophysiol* 2007; **18**: 907-13.

[44] Calkins H, Kuck KH, Cappato R, Brugada J, Camm AJ, Chen SA, et al. 2012 HRS/EHRA/ECAS expert consensus statement on catheter and surgical ablation of atrial fibrillation: recommendations for patient selection, procedural techniques, patient management and follow-up, definitions, endpoints, and research trial design: a report of the Heart Rhythm Society (HRS) Task Force on Catheter and Surgical Ablation of Atrial Fibrillation. Developed in partnership with the European Heart Rhythm Association (EHRA), a registered branch of the European Society of Cardiology (ESC) and the European Cardiac Arrhythmia Society (ECAS); and in collaboration with the American College of Cardiology (ACC), American Heart Association (AHA), the Asia Pacific Heart Rhythm Society (APHRS), and the Society of Thoracic Surgeons (STS). Endorsed by the governing bodies of the American College of Cardiology Foundation, the American Heart Association, the European Cardiac Arrhythmia Society, the European Heart Rhythm Association, the Society of Thoracic Surgeons, the Asia Pacific Heart Rhythm Society, and the Heart Rhythm Society. *Heart Rhythm* 2012; **9**: 632-96 e21.

[45] Verma A, Macle L, Cox J, Skanes AC. Canadian Cardiovascular Society atrial fibrillation guidelines 2010: catheter ablation for atrial fibrillation/atrial flutter. *Can J Cardiol* 2011; **27**: 60-6.

[46] Kobza R, Hindricks G, Tanner H, Schirdewahn P, Dorszewski A, Piorkowski C, et al. Late recurrent arrhythmias after ablation of atrial fibrillation: incidence, mechanisms, and treatment. *Heart Rhythm* 2004; **1**: 676-83.

[47] Ouyang F, Ernst S, Chun J, Bansch D, Li Y, Schaumann A, et al. Electrophysiological findings during ablation of persistent atrial fibrillation with electroanatomic mapping and double Lasso catheter technique. *Circulation* 2005; **112**: 3038-48.

[48] Calkins H, Reynolds MR, Spector P, Sondhi M, Xu Y, Martin A, et al. Treatment of atrial fibrillation with antiarrhythmic drugs or radiofrequency ablation: two systematic literature reviews and meta-analyses. *Circ Arrhythm Electrophysiol* 2009; **2**: 349-61.

[49] Weerasooriya R, Khairy P, Litalien J, Macle L, Hocini M, Sacher F, et al. Catheter ablation for atrial fibrillation: are results maintained at 5 years of follow-up? *J Am Coll Cardiol* 2011; **57**: 160-6.

[50] Khairy P, Dubuc M. Transcatheter cryoablation part I: preclinical experience. *Pacing Clin Electrophysiol* 2008; **31**: 112-20.
[51] Baust J, Gage AA, Ma H, Zhang CM. Minimally invasive cryosurgery--technological advances. *Cryobiology* 1997; **34**: 373-84.
[52] Meryman HT. Mechanics of freezing in living cells and tissues. *Science* 1956; **124**: 515-21.
[53] Gage AA, Guest K, Montes M, Caruana JA, Whalen DA, Jr. Effect of varying freezing and thawing rates in experimental cryosurgery. *Cryobiology* 1985; **22**: 175-82.
[54] Budman H, Shitzer A, Dayan J. Analysis of the inverse problem of freezing and thawing of a binary solution during cryosurgical processes. *Journal of biomechanical engineering* 1995; **117**: 193-202.
[55] Mazur P. Cryobiology: the freezing of biological systems. *Science* 1970; **168**: 939-49.
[56] Gage AA, Baust JM, Baust JG. Experimental cryosurgery investigations in vivo. *Cryobiology* 2009; **59**: 229-43.
[57] Dubuc M, Roy D, Thibault B, Ducharme A, Tardif JC, Villemaire C, et al. Transvenous catheter ice mapping and cryoablation of the atrioventricular node in dogs. *Pacing Clin Electrophysiol* 1999; **22**: 1488-98.
[58] Dubuc M, Talajic M, Roy D, Thibault B, Leung TK, Friedman PL. Feasibility of cardiac cryoablation using a transvenous steerable electrode catheter. *J Interv Card Electrophysiol* 1998; **2**: 285-92.
[59] Andrade JG, Dubuc M, Guerra PG, Coulombe N, Landry E, Rivard L, et al. Efficacy of Pulmonary Vein Isolation with a Single Application using a Novel Cryoballoon Catheter. *Heart Rhythm* 2012; **[PO04-131]**.
[60] McDonnell K, Yarkoni A, Desser K, Su W. Clinical Outcomes of Novel Cryoablation of Atrial Fibrillation. *Heart Rhythm* 2010; **[PO2-45]**
[61] Hindricks G, Piorkowski C, Tanner H, Kobza R, Gerds-Li JH, Carbucicchio C, et al. Perception of atrial fibrillation before and after radiofrequency catheter ablation: relevance of asymptomatic arrhythmia recurrence. *Circulation* 2005; **112**: 307-13.
[62] Verma A, Minor S, Kilicaslan F, Patel D, Hao S, Beheiry S, et al. Incidence of atrial arrhythmias detected by permanent pacemakers (PPM) post-pulmonary vein antrum isolation (PVAI) for atrial fibrillation (AF): correlation with symptomatic recurrence. *J Cardiovasc Electrophysiol* 2007; **18**: 601-6.
[63] Oral H, Veerareddy S, Good E, Hall B, Cheung P, Tamirisa K, et al. Prevalence of asymptomatic recurrences of atrial fibrillation after successful radiofrequency catheter ablation. *J Cardiovasc Electrophysiol* 2004; **15**: 920-4.
[64] Flaker GC, Belew K, Beckman K, Vidaillet H, Kron J, Safford R, et al. Asymptomatic atrial fibrillation: demographic features and prognostic information from the Atrial Fibrillation Follow-up Investigation of Rhythm Management (AFFIRM) study. *Am Heart J* 2005; **149**: 657-63.
[65] Nergardh A, Frick M. Perceived heart rhythm in relation to ECG findings after direct current cardioversion of atrial fibrillation. *Heart* 2006; **92**: 1244-7.
[66] Arya A, Piorkowski C, Sommer P, Kottkamp H, Hindricks G. Clinical implications of various follow up strategies after catheter ablation of atrial fibrillation. *Pacing Clin Electrophysiol* 2007; **30**: 458-62.
[67] Neumann T, Vogt J, Schumacher B, Dorszewski A, Kuniss M, Neuser H, et al. Circumferential pulmonary vein isolation with the cryoballoon technique results from a prospective 3-center study. *J Am Coll Cardiol* 2008; **52**: 273-8.
[68] Nadji G, Hermida JS, Kubala M, Quenum S, Mouquet V, Traulle S, et al. Dual balloon size strategy for cryoisolation of the pulmonary veins in patients with atrial fibrillation: comparison of 23 and 28mm diameter cryoballoons. *Arch Cardiovasc Dis* 2011; **104**: 70-6.
[69] Calkins H, Brugada J, Packer DL, Cappato R, Chen SA, Crijns HJ, et al. HRS/EHRA/ECAS expert Consensus Statement on catheter and surgical ablation of atrial fibrillation: recommendations for personnel, policy, procedures and follow-up. A report of the Heart

Rhythm Society (HRS) Task Force on catheter and surgical ablation of atrial fibrillation. *Heart Rhythm* 2007; **4**: 816-61.

[70] Jais P, Sanders P, Hsu LF, Hocini M, Haissaguerre M. Catheter ablation for atrial fibrillation. *Heart* 2005; **91**: 7-9.

[71] Haissaguerre M, Jais P, Shah DC, Garrigue S, Takahashi A, Lavergne T, et al. Electrophysiological end point for catheter ablation of atrial fibrillation initiated from multiple pulmonary venous foci. *Circulation* 2000; **101**: 1409-17.

[72] Nolker G, Heintze J, Gutleben KJ, Muntean B, Putz V, Yalda A, et al. Cryoballoon pulmonary vein isolation supported by intracardiac echocardiography: integration of a nonfluoroscopic imaging technique in atrial fibrillation ablation. *J Cardiovasc Electrophysiol* 2010; **21**: 1325-30.

[73] Schmidt M, Daccarett M, Marschang H, Ritscher G, Turschner O, Brachmann J, et al. Intracardiac echocardiography improves procedural efficiency during cryoballoon ablation for atrial fibrillation: a pilot study. *J Cardiovasc Electrophysiol* 2010; **21**: 1202-7.

[74] Siklody CH, Minners J, Allgeier M, Allgeier HJ, Jander N, Weber R, et al. Cryoballoon pulmonary vein isolation guided by transesophageal echocardiography: novel aspects on an emerging ablation technique. *J Cardiovasc Electrophysiol* 2009; **20**: 1197-202.

[75] Green MS, Guerra PG, Krahn AD. 2010 Canadian Cardiovascular Society/Canadian Heart Rhythm Society Training Standards and Maintenance of Competency in Adult Clinical Cardiac Electrophysiology. *Can J Cardiol* 2011; **27**: 859-61.

[76] Naccarelli GV, Conti JB, DiMarco JP, Tracy CM. Task force 6: training in specialized electrophysiology, cardiac pacing, and arrhythmia management endorsed by the Heart Rhythm Society. *J Am Coll Cardiol* 2008; **51**: 374-80.

[77] Hoyt H, Bhonsale A, Chilukuri K, Alhumaid F, Needleman M, Edwards D, et al. Complications arising from catheter ablation of atrial fibrillation: temporal trends and predictors. *Heart Rhythm* 2011; **8**: 1869-74.

[78] Van Belle Y, Janse P, Rivero-Ayerza MJ, Thornton AS, Jessurun ER, Theuns D, et al. Pulmonary vein isolation using an occluding cryoballoon for circumferential ablation: feasibility, complications, and short-term outcome. *Eur Heart J* 2007; **28**: 2231-7.

[79] Sauer WH, McKernan ML, Lin D, Gerstenfeld EP, Callans DJ, Marchlinski FE. Clinical predictors and outcomes associated with acute return of pulmonary vein conduction during pulmonary vein isolation for treatment of atrial fibrillation. *Heart Rhythm* 2006; **3**: 1024-8.

[80] Rajappan K, Kistler PM, Earley MJ, Thomas G, Izquierdo M, Sporton SC, et al. Acute and chronic pulmonary vein reconnection after atrial fibrillation ablation: a prospective characterization of anatomical sites. *Pacing Clin Electrophysiol* 2008; **31**: 1598-605.

[81] Dorwarth U, Schmidt M, Wankerl M, Krieg J, Straube F, Hoffmann E. Pulmonary vein electrophysiology during cryoballoon ablation as a predictor for procedural success. *J Interv Card Electrophysiol* 2011.

[82] Ahmed H, Neuzil P, Skoda J, D'Avila A, Donaldson DM, Laragy MC, et al. The permanency of pulmonary vein isolation using a balloon cryoablation catheter. *J Cardiovasc Electrophysiol* 2010; **21**: 731-7.

[83] Chierchia GB, de Asmundis C, Muller-Burri SA, Sarkozy A, Capulzini L, Paparella G, et al. Early recovery of pulmonary vein conduction after cryoballoon ablation for paroxysmal atrial fibrillation: a prospective study. *Europace* 2009; **11**: 445-9.

[84] Chun KR, Furnkranz A, Metzner A, Schmidt B, Tilz R, Zerm T, et al. Cryoballoon pulmonary vein isolation with real-time recordings from the pulmonary veins. *J Cardiovasc Electrophysiol* 2009; **20**: 1203-10.

[85] Furnkranz A, Chun KR, Nuyens D, Metzner A, Koster I, Schmidt B, et al. Characterization of conduction recovery after pulmonary vein isolation using the "single big cryoballoon" technique. *Heart Rhythm* 2010; **7**: 184-90.

[86] Chierchia GB, Yazaki Y, Sorgente A, Capulzini L, de Asmundis C, Sarkozy A, et al. Transient atriovenous reconnection induced by adenosine after successful pulmonary vein isolation with the cryothermal energy balloon. *Europace* 2009; **11**: 1606-11.

[87] Jiang CY, Jiang RH, Matsuo S, Liu Q, Fan YQ, Zhang ZW, et al. Early detection of pulmonary vein reconnection after isolation in patients with paroxysmal atrial fibrillation: a comparison of ATP-induction and reassessment at 30 minutes postisolation. *J Cardiovasc Electrophysiol* 2009; **20**: 1382-7.

[88] Hsieh MH, Tai CT, Lee SH, Lin YK, Tsao HM, Chang SL, et al. The different mechanisms between late and very late recurrences of atrial fibrillation in patients undergoing a repeated catheter ablation. *J Cardiovasc Electrophysiol* 2006; **17**: 231-5.

[89] Mainigi SK, Sauer WH, Cooper JM, Dixit S, Gerstenfeld EP, Callans DJ, et al. Incidence and predictors of very late recurrence of atrial fibrillation after ablation. *J Cardiovasc Electrophysiol* 2007; **18**: 69-74.

[90] Weerasooriya R, Khairy P, Litalien J, Macle L, Hocini M, Sacher F, et al. Catheter ablation for atrial fibrillation: are results maintained at 5 years of follow-up? *J Am Coll Cardiol (In press)*.

[91] Jiang H, Lu Z, Lei H, Zhao D, Yang B, Huang C. Predictors of early recurrence and delayed cure after segmental pulmonary vein isolation for paroxysmal atrial fibrillation without structural heart disease. *J Interv Card Electrophysiol* 2006; **15**: 157-63.

[92] Oral H, Knight BP, Ozaydin M, Tada H, Chugh A, Hassan S, et al. Clinical significance of early recurrences of atrial fibrillation after pulmonary vein isolation. *J Am Coll Cardiol* 2002; **40**: 100-4.

[93] Bertaglia E, Stabile G, Senatore G, Zoppo F, Turco P, Amellone C, et al. Predictive value of early atrial tachyarrhythmias recurrence after circumferential anatomical pulmonary vein ablation. *Pacing Clin Electrophysiol* 2005; **28**: 366-71.

[94] O'Donnell D, Furniss SS, Dunuwille A, Bourke JP. Delayed cure despite early recurrence after pulmonary vein isolation for atrial fibrillation. *Am J Cardiol* 2003; **91**: 83-5.

[95] Andrade JG, Khairy P, Verma A, Guerra PG, Dubuc M, Rivard L, et al. Early Recurrence of Atrial Tachyarrhythmias Following Radiofrequency Catheter Ablation of Atrial Fibrillation. *Pacing Clin Electrophysiol* 2011.

APPENDIX A: Timetable of visits and procedures

	SCREENING	ABLATION		FOLLOW-UP			
	Day -30 to -90	Day 0	Discharge	1 week	3 months	6 months	12 months
**Consent**	X						
**Telephone Interview**				X			
**Clinical examination**	X		X		X	X	X
**Laboratory test (K+, hemoglobin, creatinine)**		X					
**Echocardiography**	X						
**ECG**	X	X	X		X	X	X
**24 Hour Holter**	X				X	X	X
**QOL questionnaire**	X					X	X
**ILR Implantation**	X						
**ILR Interrogation**		X			X	X	X
**Cardiac CT or MRI**	X				X	X	X

APPENDIX B: Definitions of the Major complications associated with AF Ablation

Major complications:	Complication that results in permanent injury or death, requires intervention for treatment, or prolongs or requires hospitalization. AF/AFL/AT recurrence within 3 months post ablation that requires or prolongs a patient hospitalization should not be considered to be a major complication.
Atrio-Esophageal Fistulae:	Connection between the atrium and the lumen of the esophagus.
Cardiac perforation / Tamponade:	Development of a significant pericardial effusion during or within 30 days post ablation. It should be classified as early or late depending on whether it is diagnosis during or following initial discharge from the hospital.
Phrenic nerve palsy:	Absence of phrenic nerve function resulting in temporary or permanent impairment of diaphragmatic function.
Pericarditis:	An inflammation of the pericardium typically resulting in chest pain. It should be considered as major complication if it results in an effusion that leads to hemodynamic compromise or requires pericardiocentesis, prolongs hospitalization by more than 48hrs, requires hospitalization or persists for more than 30 days.
Pulmonary vein stenosis:	Is defined as a reduction of the diameter of PV or PV branch as mild <50%, moderate 50-70% or severe > 70%. A severe PV stenosis should be considered a major complication
Stroke:	Stroke is defined as an acute episode of focal or global neurological dysfunction as a result of haemorrhage or infarction. ≥24 h; OR 24 h if available neuroimaging documents a new haemorrhage or infarct; OR the neurological deficit results in death
TIA (trans-ischemic attack):	Transient episode of focal or global neurological dysfunction, without acute infarction. 24 h, any variable neuroimaging does not demonstrate a new haemorrhage or infarct.

APPENDIX C: Declaration of Helsinki

Adopted by the 18th WMA General Assembly, Helsinki, Finland, June 1964
and amended by the:
29th WMA General Assembly, Tokyo, Japan, October 1975
35th WMA General Assembly, Venice, Italy, October 1983
41st WMA General Assembly, Hong Kong, September 1989
48th WMA General Assembly, Somerset West, South Africa, October 1996
52nd WMA General Assembly, Edinburgh, Scotland, October 2000
53rd WMA General Assembly, Washington, DC, USA, October 2002
(Note of Clarification on paragraph 29 added)
55th WMA General Assembly, Tokyo, Japan, October 2004
(Note of Clarification on Paragraph 30 added)
59th WMA General Assembly, Seoul, Korea, October 2008

A. INTRODUCTION

1. The World Medical Association (WMA) has developed the Declaration of Helsinki as a statement of ethical principles for medical research involving human subjects, including research on identifiable human material and data.
   The Declaration is intended to be read as a whole and each of its constituent paragraphs should not be applied without consideration of all other relevant paragraphs.
2. Although the Declaration is addressed primarily to physicians, the WMA encourages other participants in medical research involving human subjects to adopt these principles.
3. It is the duty of the physician to promote and safeguard the health of patients, including those who are involved in medical research. The physician's knowledge and conscience are dedicated to the fulfillment of this duty.
4. The Declaration of Geneva of the WMA binds the physician with the words, "The health of my patient will be my first consideration," and the International Code of Medical Ethics declares that, "A physician shall act in the patient's best interest when providing medical care."
5. Medical progress is based on research that ultimately must include studies involving human subjects. Populations that are underrepresented in medical research should be provided appropriate access to participation in research.
6. In medical research involving human subjects, the well-being of the individual research subject must take precedence over all other interests.
7. The primary purpose of medical research involving human subjects is to understand the causes, development and effects of diseases and improve preventive, diagnostic and therapeutic interventions (methods, procedures and treatments). Even the best current interventions must be evaluated continually through research for their safety, effectiveness, efficiency, accessibility and quality.
8. In medical practice and in medical research, most interventions involve risks and burdens.
9. Medical research is subject to ethical standards that promote respect for all human subjects and protect their health and rights. Some research populations are particularly vulnerable and need special protection. These include those who cannot give or refuse

consent for themselves and those who may be vulnerable to coercion or undue influence.

10. Physicians should consider the ethical, legal and regulatory norms and standards for research involving human subjects in their own countries as well as applicable international norms and standards. No national or international ethical, legal or regulatory requirement should reduce or eliminate any of the protections for research subjects set forth in this Declaration.

B. BASIC PRINCIPLES FOR ALL MEDICAL RESEARCH

11. It is the duty of physicians who participate in medical research to protect the life, health, dignity, integrity, right to self-determination, privacy, and confidentiality of personal information of research subjects.

12. Medical research involving human subjects must conform to generally accepted scientific principles, be based on a thorough knowledge of the scientific literature, other relevant sources of information, and adequate laboratory and, as appropriate, animal experimentation. The welfare of animals used for research must be respected.

13. Appropriate caution must be exercised in the conduct of medical research that may harm the environment.

14. The design and performance of each research study involving human subjects must be clearly described in a research protocol. The protocol should contain a statement of the ethical considerations involved and should indicate how the principles in this Declaration have been addressed. The protocol should include information regarding funding, sponsors, institutional affiliations, other potential conflicts of interest, incentives for subjects and provisions for treating and/or compensating subjects who are harmed as a consequence of participation in the research study. The protocol should describe arrangements for post-study access by study subjects to interventions identified as beneficial in the study or access to other appropriate care or benefits.

15. The research protocol must be submitted for consideration, comment, guidance and approval to a research EC before the study begins. This committee must be independent of the researcher, the sponsor and any other undue influence. It must take into consideration the laws and regulations of the country or countries in which the research is to be performed as well as applicable international norms and standards but these must not be allowed to reduce or eliminate any of the protections for research subjects set forth in this Declaration. The committee must have the right to monitor ongoing studies. The researcher must provide monitoring information to the committee, especially information about any SAEs. No change to the protocol may be made without consideration and approval by the committee.

16. Medical research involving human subjects must be conducted only by individuals with the appropriate scientific training and qualifications. Research on patients or healthy volunteers requires the supervision of a competent and appropriately qualified physician or other health care professional. The responsibility for the protection of research subjects must always rest with the physician or other health care professional and never the research subjects, even though they have given consent.

17. Medical research involving a disadvantaged or vulnerable population or community is only justified if the research is responsive to the health needs and priorities of this population or community and if there is a reasonable likelihood that this population or community stands to benefit from the results of the research.

18. Every medical research study involving human subjects must be preceded by careful assessment of predictable risks and burdens to the individuals and communities involved in the research in comparison with foreseeable benefits to them and to other individuals or communities affected by the condition under investigation.
19. Every clinical trial must be registered in a publicly accessible database before recruitment of the first subject.
20. Physicians may not participate in a research study involving human subjects unless they are confident that the risks involved have been adequately assessed and can be satisfactorily managed. Physicians must immediately stop a study when the risks are found to outweigh the potential benefits or when there is conclusive proof of positive and beneficial results.
21. Medical research involving human subjects may only be conducted if the importance of the objective outweighs the inherent risks and burdens to the research subjects.
22. Participation by competent individuals as subjects in medical research must be voluntary. Although it may be appropriate to consult family members or community leaders, no competent individual may be enrolled in a research study unless he or she freely agrees.
23. Every precaution must be taken to protect the privacy of research subjects and the confidentiality of their personal information and to minimize the impact of the study on their physical, mental and social integrity.
24. In medical research involving competent human subjects, each potential subject must be adequately informed of the aims, methods, sources of funding, any possible conflicts of interest, institutional affiliations of the researcher, the anticipated benefits and potential risks of the study and the discomfort it may entail, and any other relevant aspects of the study. The potential subject must be informed of the right to refuse to participate in the study or to withdraw consent to participate at any time without reprisal. Special attention should be given to the specific information needs of individual potential subjects as well as to the methods used to deliver the information. After ensuring that the potential subject has understood the information, the physician or another appropriately qualified individual must then seek the potential subject's freely-given informed consent, preferably in writing. If the consent cannot be expressed in writing, the non-written consent must be formally documented and witnessed.
25. For medical research using identifiable human material or data, physicians must normally seek consent for the collection, analysis, storage and/or reuse. There may be situations where consent would be impossible or impractical to obtain for such research or would pose a threat to the validity of the research. In such situations the research may be done only after consideration and approval of a research EC.
26. When seeking informed consent for participation in a research study the physician should be particularly cautious if the potential subject is in a dependent relationship with the physician or may consent under duress. In such situations the informed consent should be sought by an appropriately qualified individual who is completely independent of this relationship.
27. For a potential research subject who is incompetent, the physician must seek informed consent from the legally authorized representative. These individuals must not be included in a research study that has no likelihood of benefit for them unless it is intended to promote the health of the population represented by the potential subject, the research cannot instead be performed with competent persons, and the research entails only minimal risk and minimal burden.

28. When a potential research subject who is deemed incompetent is able to give assent to decisions about participation in research, the physician must seek that assent in addition to the consent of the legally authorized representative. The potential subject's dissent should be respected.

29. Research involving subjects who are physically or mentally incapable of giving consent, for example, unconscious patients, may be done only if the physical or mental condition that prevents giving informed consent is a necessary characteristic of the research population. In such circumstances the physician should seek informed consent from the legally authorized representative. If no such representative is available and if the research cannot be delayed, the study may proceed without informed consent provided that the specific reasons for involving subjects with a condition that renders them unable to give informed consent have been stated in the research protocol and the study has been approved by a research EC. Consent to remain in the research should be obtained as soon as possible from the subject or a legally authorized representative.

30. Authors, editors and publishers all have ethical obligations with regard to the publication of the results of research. Authors have a duty to make publicly available the results of their research on human subjects and are accountable for the completeness and accuracy of their reports. They should adhere to accepted guidelines for ethical reporting. Negative and inconclusive as well as positive results should be published or otherwise made publicly available. Sources of funding, institutional affiliations and conflicts of interest should be declared in the publication. Reports of research not in accordance with the principles of this Declaration should not be accepted for publication.

C. ADDITIONAL PRINCIPLES FOR MEDICAL RESEARCH COMBINED WITH MEDICAL CARE

31. The physician may combine medical research with medical care only to the extent that the research is justified by its potential preventive, diagnostic or therapeutic value and if the physician has good reason to believe that participation in the research study will not adversely affect the health of the patients who serve as research subjects.

32. The benefits, risks, burdens and effectiveness of a new intervention must be tested against those of the best current proven intervention, except in the following circumstances:

- The use of placebo, or no treatment, is acceptable in studies where no current proven intervention exists; or
- Where for compelling and scientifically sound methodological reasons the use of placebo is necessary to determine the efficacy or safety of an intervention and the patients who receive placebo or no treatment will not be subject to any risk of serious or irreversible harm. Extreme care must be taken to avoid abuse of this option.

33. At the conclusion of the study, patients entered into the study are entitled to be informed about the outcome of the study and to share any benefits that result from it, for example, access to interventions identified as beneficial in the study or to other appropriate care or benefits.

34. The physician must fully inform the patient which aspects of the care are related to the research. The refusal of a patient to participate in a study or the patient's decision to withdraw from the study must never interfere with the patient-physician relationship.

35. In the treatment of a patient, where proven interventions do not exist or have been ineffective, the physician, after seeking expert advice, with informed consent from the patient or a legally authorized representative, may use an unproven intervention if in the physician's judgment it offers hope of saving life, re-establishing health or alleviating

suffering. Where possible, this intervention should be made the object of research, designed to evaluate its safety and efficacy. In all cases, new information should be recorded and, where appropriate, made publicly available.

CIRCA DOSE

Montreal Health Innovations
Coordinating Center | MHICC
A Division of the Montreal Heart Institute

PROTOCOL ADDENDUM 1

Study Title: CIRCA-DOSE (Cryoballon vs Irrigated Radiofrequency Catheter Abaltion: The effect of double-short vs standard exposure cryoablation duration during pulmonary vein isolation)

Protocol number: 2055

Version date: 27 June 2014

Sponsor: Dr Jason Andrade
University of British Columbia (UBC)

Addendum Type: Change in protocol requirement: Implantable loop recorder

Addendum Purpose: Reflect current site clinical standard practices

Addendum Summary:
In sub-section 2.2.2 (Duration of treatment and follow-up period) of Section 2.2 (Study Design) of the protocol, it is stated that *"An implantable loop recorder will be implanted 30 to 90 days prior to the index ablation to all patients that have signed the consent form"*.

In sub-section 2.2.4 (Implantable Loop Recorder), of the same section, it is indicated that *"The patients will undergo a loop recorder implantation (REVEAL LINQ, Medtronic), also called an insertable cardiac monitor (ICM), at a minimum of 30 days (but ideally 90 days) prior to the planned ablation procedure"*.

The sites' standard practices for patients undergoing ablations demonstrate that a scheduled procedure normally exceeds 90 days of waiting time.

Hence, to reflect the current standard practices implemented within sites, the maximum timeline of 90 days prior to ablation is to be removed.

This addendum aims to specify in sections 2.2.2 and 2.2.4 of the protocol, that *"the loop recorder implantation (Reveal LINQ, Medtronic), will be performed at a minimum of 30 days prior to the planned ablation procedures"*.

[illegible]

Dr Jason Andrade, MD
Study Principal Investigator, Sponsor

Feb 1/16

Date

CIRCA DOSE

Montreal Health Innovations
Coordinating Center
A Division of the Montreal Heart Institute
MHICC

PROTOCOL ADDENDUM 2

Study Title: CIRCA-DOSE (Cryoballon vs Irrigated Radiofrequency Catheter Ablation: The effect of double-short vs standard exposure cryoablation duration during pulmonary vein isolation)

Protocol number: 2055

Version date: 27 June 2014

Sponsor: Dr Jason Andrade
University of British Columbia (UBC)

Addendum type: Change in Inclusion Criteria

Addendum Purpose: Reflect current CCS recommendations on AF patient's management and ablation.

Addendum Summary:

In sub-section 2.1.2.1 (Inclusion criteria), of section 2.1.2 (Eligibility Criteria) the third inclusion criteria states "*Candidate for ablation based on AF that is symptomatic and refractory (ineffective or intolerant) to at least one class 1 or 3 antiarrhythmic*".

The 2014 Focused Update of the CCS Guidelines for the Management of AF patients, a tool that features essential diagnostic and treatment recommendations suggests using catheter ablation to maintain sinus rhythm as first-line therapy for relief of symptoms in highly selected patients with symptomatic paroxysmal AF. Thus, sites participating in the study are applying these current recommendations into their standard clinical practices hence, referring patients for ablation as a first line therapy.

This addendum aims to add a specification to this particular inclusion criterion of sub-sections 2.1.2.1 of the protocol, to allow investigators in the inclusion of patients into the study, who would be referred for a catheter ablation procedure as a first line therapy.

[signature]

Dr Jason Andrade, MD
Study Principal Investigator, Sponsor

feb 1 16

Date

STATISTICAL ANALYSIS PLAN

Protocol number: 2055

Cryoballoon vs. Irrigated Radiofrequency Catheter Ablation: The effect of Double Short vs. Standard Exposure Cryoablation Duration During Pulmonary Vein Isolation

The CIRCA-DOSE Trial

Date Statistical Analysis Plan (Final): 23-NOV-2015

Prepared by:
Sylvie Levesque MSc
Biostatistician
Montreal Health Innovations Coordinating Center (MHICC)
4100 Molson, Suite 400
Montréal, QC H1Y 3N1
Phone: 514-461-1300 ext. 2890 / Fax: 514-461-1301

Signature Approval Page

By signing below, I indicate that I have reviewed the Statistical Analysis Plan in its entirety and approve its contents.

Signature: ______________________ Date: Nov 24 2015

Jason Andrade, MD
Principal investigator / Sponsor
Vancouver General Hospital

Signature: ______________________ Date: 24 NOV 2015

Marie-Claude Guertin, Ph.D.
Head, Biostatistics
MHICC

Revision History

Version	Date (DD-MMM-YYYY)	Author	Summary of Changes
Final			

TABLE OF CONTENTS

List of Abbreviations 6
1 Introduction 7
2 Study Description 7
2.1 Study Design 7
2.2 Study Objectives 8
3 Datasets Analyzed 8
3.1 Intent-To-Treat (ITT) Population 8
3.2 Evaluable Population 8
3.3 Implanted Population 9
4 Efficacy Endpoints 9
4.1 Primary Efficacy Endpoint 9
4.2 Secondary Efficacy Endpoints 10
5 Other Endpoints and Procedure Parameters 13
5.1 Atrial Arrhythmia (as Per Patient's Assessment) 14
5.2 Implantable Loop Recorder (ILR) 14
5.3 Ablation Procedure Parameters 14
6 Safety endpoints 14
6.1 Concomitant Medications 14
6.2 Adverse Events and Serious Adverse Events 14
6.3 Vital Signs, 12-Lead ECG and Physical Exam 14
6.4 Other Exams 15
6.5 Laboratory Parameters 15
6.6 Childbearing Potential 15
7 Statistical Methodology 15
7.1 Statistical Considerations 15
7.2 Study Patients 15
7.2.1 Patient Disposition 15
7.2.2 Datasets Analyzed 16
7.2.3 Demographic and Baseline Characteristics 16
7.2.4 Compliance 16
7.3 Efficacy Analysis 16
7.3.1 Primary Analysis 16
7.3.2 Secondary Analysis 17
7.3.3 Other Pre Specified Analyses 19
7.4 Other Endpoints and Procedure Parameters Analysis 20
7.4.1 Atrial Arrhythmia Assessment 20
7.4.2 Implantable Loop Recorder (ILR) 20
7.4.3 Ablation Procedure Parameters 20
7.5 Safety Parameters Analysis 21
7.5.1 Concomitant Medications 21
7.5.2 Adverse Events and Serious Adverse Events 21
7.5.3 Vital Signs, 12-Lead ECG and Physical Exam Analysis 21
7.5.4 Other Exam Analysis 22

7.5.5 Laboratory Parameters ... 22
7.5.6 Childbearing Potential ... 22
7 Summary Tables, Figures and Listings ... 23
8 References ... 35

LIST OF ABBREVIATIONS

**AAD**	Antiarrhythmic drug
**AE**	Adverse Event
**AF**	Atrial Fibrillation
**AFEQT**	Atrial Fibrillation Effect on Quality of life
**AFL**	Atrial Flutter
**ANOVA**	Analysis of Variance
**AT**	Atrial Tachycardia
**CRF**	Case Report Form
**CT**	Computerized Tomography
**ECG**	Electrocardiogram
**EQ-5D-3L**	EuroQoL-5D-3L
**HRQoL**	Health-related quality of life
**ILR**	Implantable Loop Recorder
**ITT**	Intent-To-Treat
**MHICC**	Montreal Health Innovations Coordinating Center
**MRI**	Magnetic Resonance Imaging
**PV**	Pulmonary Vein
**PVI**	Pulmonary Vein Isolation
**QoL**	Quality of Life
**RFCA**	Radiofrequency catheter ablation
**SAE**	Serious Adverse Event
**SAP**	Statistical Analysis Plan
**TTE**	Transthoracic Echocardiogram
**VAS**	Visual analogue scale

1 Introduction

The purpose of this statistical analysis plan (SAP) is to present the statistical methodology that will be used for the analysis of the CIRCA-DOSE trial. This plan also provides a description of the tables, figures and listings that will be included in the final statistical report. In case of differences between the SAP and the protocol, the SAP will supersede the protocol.

2 Study Description

2.1 Study Design

This will be a prospective randomized trial. Patients with symptomatic non-permanent atrial fibrillation (AF) refractory to at least one antiarrhythmic drug (AAD) referred for percutaneous catheter ablation and meeting inclusion criteria will be enrolled. Patients will be randomized (blinded to their allocation assignment and stratified by center) in a 1:1:1 allocation ratio to pulmonary vein isolation (PVI) using:

- **Group 1 / RFCA:** Standard radiofrequency ablation guided by tissue-contact force;
- **Group 2 / CRYO 4:** Standard cryoballoon ablation duration (4 minute applications with a 4 minute insurance freeze after isolation is achieved);
- **Group 3 / CRYO 2:** Short cryoballoon ablation (2 minute applications with a 2 minute insurance freeze after isolation is achieved).

Randomization will use blocks and will be stratified by site. The randomization schema is presented below.

Figure 1: Randomization schema

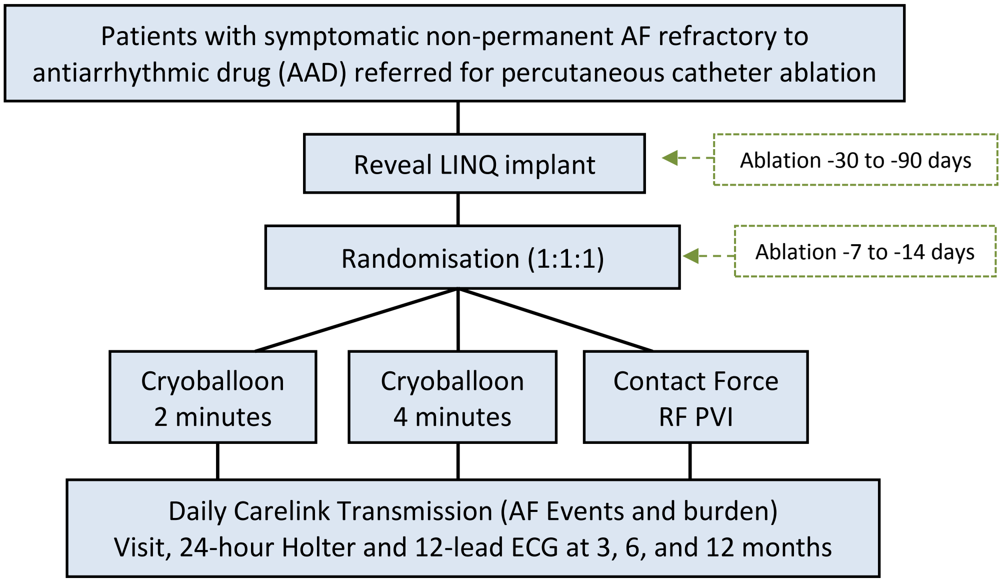

All patients randomized will be evaluated according to the schedule below.

	SCREENING	ABLATION		FOLLOW-UP			
	Day -30 to -90	Day 1	Discharge	1 week	3 months	6 months	12 months
Consent	X						
Telephone Interview				X			
Clinical examination	X		X		X	X	X
Laboratory test (K+, hemoglobin, creatinine)		X					
Echocardiography	X						
ECG	X	X	X		X	X	X
24 Hour Holter	X				X	X	X
QOL questionnaire	X					X	X
ILR Implantation	X						
ILR Interrogation*		X			X	X	X
Cardiac CT or MRI	X				X	X	X

Note: Day 1 = index ablation date (CRF p.18)
* if not capable of performing daily transmissions

A total of 348 patients are to be randomized into the study.

2.2 Study Objectives

The primary objective of this study is to verify if repeated freezing cycles (4 minutes or 2 minutes) are more efficacious (i.e., fewer recurrence of AF), and safer, than the established standard radiofrequency catheter ablation (RFCA).

3 DATASETS ANALYZED

3.1 Intent-To-Treat (ITT) Population

The ITT population will consist of all randomized and ablated patients as recorded in CRF p.15 and p.18 respectively. In the ITT population, patients allocated to a treatment strategy group by randomization will be followed up, assessed and analyzed as members of that group irrespective of their compliance to the planned course of treatment strategy. Patients randomized by error (ex. not meeting inclusion/exclusion criteria) will be reviewed and possibly removed from the ITT population.

3.2 Evaluable Population

The evaluable population will consist of patients of the ITT population, excluding patients with major deviations from the protocol. The list of patients to be excluded will be provided by the principal investigator.

3.3 Implanted Population

The implanted population will consist of all patients who had a successfully implantable loop recorder (ILR) implantation (CRF p.14). All randomized patients will be included (CRF p.15), along with patients who had a successfully ILR implantation, but were not randomized.

4 EFFICACY ENDPOINTS

4.1 Primary Efficacy Endpoint

The primary endpoint will be the time to first recurrence of symptomatic or asymptomatic (any) AF, atrial flutter, or atrial tachycardia (AF/AFL/AT) documented by 12-lead ECG, surface ECG rhythm strips, ambulatory ECG monitor, or on implantable loop recorder and lasting 120 seconds or longer as adjudicated by a blinded group of investigators between Day 91 and study completion, or a repeat ablation procedure between ablation (Day 1) and study completion. In other words, either an AF/AFL/AT between Day 91 and study completion or a repeat ablation procedure (at least one reported CRF Re-Ablation Form) between ablation and study completion will qualify as a primary endpoint. Any AF/AFL/AT episode occurring before Day 91 or after study completion will not be considered in the calculation of the primary endpoint. For a given patient, study completion will be taken as the date of study completion reported on CRF p.50.

All relevant information will be obtained from the ILR database.

Time zero will be Day 91. The first episode occurring at or after Day 91, but not after study completion, will be identified and time to event will be calculated as the number of days between Day 91 and the date of this first episode. In patients with a repeat ablation procedure between ablation and Day 90, time to event will be set to 1. Patients with no episode between Day 91 and study completion will be censored at their date of study completion. This is summarized in the table below.

Definition of primary efficacy endpoint (symptomatic or asymptomatic AF/AFL/AT + repeat ablation)

Event (failure)	=	Any AF/AFL/AT between Day 91 and Day SC (inclusive)<br>OR<br>Any repeat ablation procedure between index ablation and Day SC (inclusive)

Time to event	=	Date of first event[(a)] – Day 91 + 1	If any AF/AFL/AT or repeat ablation between Day 91 and Day SC (inclusive)
	=	1	If repeat ablation procedure between index ablation and Day 90 (inclusive)
	=	Day SC – Day 91 + 1	If no event

[(a)] Date of first event is the AF/AFL/AT date or the date of repeat ablation procedure, whichever comes first.

Note: Day X = index ablation date (CRF p.18) + (X-1) days
Day SC = study completion date (CRF p.50).

4.2 Secondary Efficacy Endpoints

The secondary endpoints will include:

1) Time to first recurrence of symptomatic documented AF/AFL/AT between Day 91 and study completion or a repeat ablation procedure between ablation procedure and study completion.

   This endpoint will be derived in a fashion similar to the primary endpoint, but with the requirement related to being symptomatic.

Definition of secondary efficacy endpoint (symptomatic AF/AFL/AT + repeat ablation)

Event (failure)	=	Symptomatic AF/AFL/AT between Day 91 and Day SC (inclusive)<br>OR<br>Any repeat ablation procedure between index ablation and Day SC (inclusive)

Time to event	=	Date of first event[(a)] – Day 91 + 1	If symptomatic AF/AFL/AT or repeat ablation between Day 91 and Day SC (inclusive)
	=	1	If repeat ablation procedure between index ablation and Day 90 (inclusive)
	=	Day SC – Day 91 + 1	If no event

[(a)] Date of first event is the symptomatic AF/AFL/AT date or the date of repeat ablation procedure, whichever comes first.

Note: Day X = index ablation date (CRF p.18) + (X-1) days
Day SC = study completion date (CRF p.50).

2) Total arrhythmia burden expressed as daily AF burden (hours/day) and as overall AF burden (% time in AF).

   AF burden will be defined for the period from Day 91 to study completion (inclusive) and will be computed as:

$$\text{Daily AF burden (hours/day)} = \frac{\text{\# of hours with AF/AFL/AT}}{(\text{Day SC} - \text{Day 91} + 1)}$$

$$\text{AF burden (\% time in AF)} = \left[\frac{\text{\# of hours with AF/AFL/AT}}{(\text{Day SC} - \text{Day 91} + 1) \times 24}\right] \times 100$$

   These endpoints will be derived using an external log that will be provided by the data management team.

3) Proportion of patients experiencing an acute or adenosine provoking PV reconnection during the index ablation procedure.

A patient will be considered has experiencing an acute or adenosine provoking PV reconnection if at least one vein with a spontaneous and/or an adenosine induced PV reconnection is reported for that patient.

This information will be collected in the CRF p.24.

4) Proportion of patients who needed repeat ablation procedure because of documented recurrence of symptomatic AF/AFL/AT between index ablation and study completion (inclusive).

   A patient will be considered has having a repeat ablation procedure if at least one Re-Ablation Form is reported for that patient.

5) Proportion of patients who needed antiarrhythmic drug because of documented recurrence of symptomatic AF/AFL/AT.

   This information will be collected using the question "Is the patient taking a Class I or Class III antiarrhythmic drug" in the CRF at phone contact (CRF p.27), 3 months (CRF p.28), 6 months (CRF p.34) and at 12 months (CRF p.40).

6) Proportion of patients with AF/AFL/AT during the first 90 days post ablation.

   This endpoint will be derived in a fashion similar to the first component of the primary endpoint, but with the requirement that the episode occurred between ablation and Day 90 (inclusive).

7) Proportion of patients with an emergency visit or hospitalization in a health-care facility.

   This information will be collected in the "Resource Utilization Form" of the CRF at Month 3, Month 6 and Month 12 (CRF p.30, 36 and 42). A patient will be considered has having gone to the emergency room at a given time point if the answer to the corresponding question is YES at that time point. The same will be done to identify patients who were hospitalized or having a composite endpoint of emergency visit and/or hospitalization.

8) Proportion of patients with a major complications including death, stroke, TIA, Myocardial Infarction or systemic thromboembolism, PV stenosis, phrenic nerve palsy, pericarditis, pericardial effusion, cardiac perforation or tamponade, hematoma, AV fistula, pseudoaneurysm, esophageal injury and atrio-esophageal fistulae (both individually and as a composite endpoint). Standard definitions as per the 2012 HRS/EHRA/ECAS recommendations will be employed. Acute peri-procedural complications will be defined as occurring within 30 days of ablation, with delayed complications occurring 31-365 days after ablation.

   These endpoints will be adjudicated by an independent event committee.

9) Overall and disease specific quality of life (QoL) including the CCS-SAF, EQ-5D and AFEQT questionnaires. The CCS-SAF questionnaire will be completed at visit baseline only, whereas the EQ-5D and AFEQT questionnaires will be completed at visits baseline, 6 months and 12 months.

The CCS-SAF score is obtained using three steps: documentation of possible AF-related symptoms (palpitations, dyspnea, dizziness/pre syncope/syncope, chest pain, weakness/fatigue); determination of symptom-rhythm correlation; and assessment of the effect of these symptoms on patient daily function and QoL. CCS-SAF score ranges from 0 (asymptomatic) to 4 (severe impact of symptoms on QoL and activities of daily living). Patients are also categorized by type of AF (paroxysmal versus persistent/permanent)[1]. The CCS-SAF score will be recorded in the CRF at p.7.

The EuroQoL-5D-3L (EQ-5D-3L) includes the following 5 dimensions: mobility, self-care, usual activities, pain/discomfort and anxiety/depression. Each dimension has the 3 following levels: no problems, some problems and extreme problems. A 5 digit code representing the state of health will be obtained for each respondent (1 digit by dimension). The respondent will also rate his/her health state by tracing a line on the vertical visual analogue scale (VAS) where the endpoints are labelled 'Best imaginable state of health' and 'Worst imaginable state of health'. The score corresponding to the states of health will be based on the value sets of the U.K. population using the VAS valuation technique[2].

The atrial fibrillation effect on quality of life (AFEQT) questionnaire is an atrial fibrillation-specific health-related quality of life (HRQoL) questionnaire which includes 20 questions. The questions provided information about how the AF affects symptoms (questions 1 to 4), daily activities (questions 5 to 12), treatment concern (questions 13 to 18) and treatment satisfaction (questions 19 and 20). The first eighteen questions include 7 levels (1 (most severe limitation/symptoms) to 7 (no limitation/symptoms)) and the last 2 questions include also 7 levels (1 (extremely satisfied) to 7 (extremely dissatisfied)).

Only the overall AFEQT score will be computed. This score ranges from 0 to 100. A score of 0 corresponds to a complete disability, while a score of 100 corresponds to no disability. The overall AFEQT score will be calculated from questions 1 to 18 according to the following definition[3].

Definition of the AFEQT scores

Score	Formula
Overall AFEQT	$= 100 - \left[\frac{(\text{sum of questions 1 to 18 answered } - \text{ number of questions answered}) \times 100}{\text{number of questions answered } \times 6}\right]$

Note: The overall AFEQT score will be calculated from questions 1 to 18

10) Single and multiple procedure success defined as freedom from <u>symptomatic or asymptomatic</u> electrocardiographically documented AF/AFL/AT after the first and last ablation procedure respectively.

Definition of secondary efficacy endpoint (any AF/AFL/AT after the 1st ablation)

Event (failure)	=	Any AF/AFL/AT between index ablation and Day SC (inclusive)

Time to event	=	Date of first AF/AFL/AT – Date of index ablation + 1	If any AF/AFL/AT between index ablation and Day SC (inclusive)
	=	Day SC – Date of index ablation + 1	If no event

Note: Day SC = study completion date (CRF p.50).

Definition of secondary efficacy endpoint (any AF/AFL/AT after the last ablation)

Event (failure)	=	Any AF/AFL/AT between last ablation[a] and Day SC (inclusive)

Time to event	=	Date of first AF/AFL/AT – Date of last ablation + 1	If any AF/AFL/AT between last ablation and Day SC (inclusive)
	=	Day SC – Date of last ablation + 1	If no event

[a] If >1 ablation then date of last ablation = last ablation date (CRF p.52), else if only 1 ablation then date of last ablation = index ablation date (CRF p.18).
Note: Day SC = study completion date (CRF p.50).

11) Single and multiple procedure success defined as freedom from symptomatic electrocardiographically documented AF/AFL/AT after the first and last ablation procedure respectively.

Definition of secondary efficacy endpoint (symptomatic AF/AFL/AT after the 1st ablation)

Event (failure)	=	Symptomatic AF/AFL/AT between index ablation and Day SC (inclusive)

Time to event	=	Date of first symptomatic AF/AFL/AT – Date of index ablation + 1	If symptomatic AF/AFL/AT between index ablation and Day SC (inclusive)
	=	Day SC – Date of index ablation + 1	If no event

Note: Day SC = study completion date (CRF p.50).

Definition of secondary efficacy endpoint (symptomatic AF/AFL/AT after the last ablation)

Event (failure)	=	Symptomatic AF/AFL/AT between last ablation[a] and Day SC (inclusive)

Time to event	=	Date of first symptomatic AF/AFL/AT – Date of last ablation + 1	If symptomatic AF/AFL/AT between last ablation and Day SC (inclusive)
	=	Day SC – Date of last ablation + 1	If no event

[a] If >1 ablation then date of last ablation = last ablation date (CRF p.52), else if only 1 ablation then date of last ablation = index ablation date (CRF p.18).
Note: Day SC = study completion date (CRF p.50).

5 OTHER ENDPOINTS AND PROCEDURE PARAMETERS

5.1 Atrial Arrhythmia (as Per Patient's Assessment)

Atrial arrhythmia parameters, as per patient assessment, will be collected in the CRF at visit 3 months (CRF p. 28), 6 months (CRF p. 34) and 12 months (CRF p. 40) and will include occurrence of symptomatic episode(s), number of symptomatic episode(s), symptoms associated with the episode(s) and severity of the most extreme symptoms.

5.2 Implantable Loop Recorder (ILR)

Information regarding the implantation of ILR will be collected at ILR implantation visit (CRF p.14), whereas the information regarding the functionality of the ILR will be assessed at visit ablation (CRF p. 16), 3 months (CRF p. 30), 6 months (CRF p. 36) and 12 months (CRF p. 42).

5.3 Ablation Procedure Parameters

Various parameters will be collected in the CRF during the index ablation procedure (CRF p. 18-25) as well as during additional ablation procedures (CRF p. 52-60) when applicable. These parameters will include presenting rhythm, procedure summary, anticoagulation therapy, antiarrhythmic drug, esophageal temperature monitoring, radiofrequency ablation, maximum RF energy used, cryoballoon ablation, phrenic monitoring and PV reconnection. Summary at the end of the ablation, including duration of the procedure (time of exit from EP lab – time of entry to EP lab) and fluoroscopy time (fluoroscopy time at time of the end of the procedure – fluoroscopy time at time of left atrial access) will also be assessed (see CRF p.25 for index ablation procedure and CRF p.60 for additional ablation procedures).

6 SAFETY ENDPOINTS

6.1 Concomitant Medications

Concomitant medications will be collected throughout the study, starting at inclusion (study entry).

6.2 Adverse Events and Serious Adverse Events

An adverse event (AE) is defined as an untoward medical occurrence in a patient. Adverse events occurring during the study will be documented in dedicated “Adverse Event” forms. Those events will be classified by the investigators according to their seriousness, and whether they are study/procedure related.

Serious adverse events (SAE) will be defined as death, life-threatening events, events causing functional disability, an event requiring or prolonging hospitalization, or any other event considered serious by an investigator. All SAEs will be adjudicated by an independent event committee.

6.3 Vital Signs, 12-Lead ECG and Physical Exam

Vital signs (heart rate and blood pressure) and a 12-lead ECG will be assessed at baseline, discharge, Month 3, Month 6 and Month 12. Height and weight will be collected at baseline only. A physical examination and a NYHA functional class will be evaluated at baseline, Month 3, Month 6 and Month 12.

6.4 Other Exams

Several exams will be performed during the study.

A 24-hour Holter monitor exam will be performed at baseline, Month 3, Month 6 and Month 12. A transthoracic echocardiogram (TTE) / nuclear exam will be performed at baseline and a CT/MRI exam will be done at baseline, Month 3, Month 6 and Month 12. PV pattern and PV dimensions will be obtained at baseline.

6.5 Laboratory Parameters

Some laboratory parameters will be collected before the ablation.

6.6 Childbearing Potential

A pregnancy test will be done before the ablation.

7 Statistical Methodology

7.1 Statistical Considerations

Statistical analyses will be performed using SAS Version 9.4 or higher. Unless otherwise specified, all statistical tests will be two-sided and performed at a significance level of 0.05.

Prior to all parametric analyses, basic assumptions will be checked and if they are violated, non-parametric analyses or data transformation will be performed to confirm results from the parametric analyses.

Descriptive statistics will be presented for most study parameters. For continuous variables, N, mean, median, standard deviation, Q1, Q3, minimum and maximum will be presented. Number of patients and proportion will be presented for categorical variables.

Unless otherwise stated, no missing value will be imputed.

7.2 Study Patients

7.2.1 Patient Disposition

Number of randomized patients, number of randomized patients completing the study and reasons for discontinuation will be summarized overall and by treatment strategy group. This information will also be presented by study site. A listing of patient disposition will be provided.

7.2.2 Datasets Analyzed

The number of patients in each datasets will be summarized overall and by treatment strategy group. A listing providing the population membership and the reasons for being excluded from each population will be provided as well.

7.2.3 Demographic and Baseline Characteristics

Demographic data (age at informed consent, gender and ethnicity) as well as baseline characteristics (medical history and atrial fibrillation history) will be summarized using descriptive statistics, overall and by treatment strategy group, for the ITT population.

Demographic and baseline characteristics will be presented overall and by group (randomized implanted patients vs not randomized implanted patients), for the implanted population.

7.2.4 Compliance

In this study, compliance refers to the patient's compliance in recording and transmitting weekly ECG rhythm strips using the implantable loop recorder monitor. For a given patient, compliance will be computed as:

ILR compliance in %:

$$\left[\frac{\text{total \# of weeks with at least one transmission for a given period of time}}{\text{total \# of weeks in the same given period of time}}\right] \times 100$$

ILR compliance: Yes if ILR compliance in % ≥ 75%
No if ILR compliance in % < 75%

ILR compliance will be calculated and summarized from ablation to study completion, for the period between ablation and Day 90 as well as for the period between Day 91 and study completion. This will be done for the ITT and evaluable populations.

7.3 Efficacy Analysis

The efficacy analyses will be primarily based on the ITT population but, for illustrative purposes, the primary and the first secondary analyses will be repeated on the evaluable population.

7.3.1 Primary Analysis

The primary analysis will be an unadjusted comparison of the time to first recurrence of symptomatic or asymptomatic documented AF/AFL/AT between Day 91 and study completion, or repeat ablation

procedure between index ablation and study completion. Survival curves will be estimated by the Kaplan-Meier method and the differences between the groups will be assessed using log rank tests. The two main comparisons will be CRYO 4 vs RFCA and CRYO 2 vs RFCA and will be conducted at the 0.025 significance level. These will be considered as the primary analysis of the study. The other comparison, CRYO 4 vs CRYO 2 will be considered as secondary.

For illustrative purposes, unadjusted hazard ratios (HR) from a Cox proportional hazards model will be provided with 97.5% confidence intervals for the HR comparing CRYO 2 and CRYO 4 vs RFCA and with 95% confidence interval for the HR comparing CRYO 4 vs CRYO 2.

7.3.2 Secondary Analysis

Unless otherwise specified, secondary analysis will be performed on the ITT population and all confidence intervals for the HR will be presented at 95% level of significance.

The following analyses will be performed for the secondary endpoints:

1) Time to first recurrence of <u>symptomatic</u> documented AF/AFL/AT between Day 91 and study completion or a repeat ablation procedure between index ablation and study completion will be analyzed as the primary endpoint using Kaplan-Meier survival curves and log-rank tests.

2) Total arrhythmia burden expressed as daily AF burden (hours/day) and as overall AF burden (% time in AF) between Day 91 and study completion will be analyzed using an analysis of variance (ANOVA). Pairwise comparisons will be done using contrasts under the ANOVA models. If the data are not normally distributed, then the non-parametric Wilcoxon Signed Rank test will be used. Descriptive statistics will be presented for the following periods: between Day 91 and Day 120, Day 121 and Day 150, Day 151 and Day 180, Day 181 and study completion and between Day 91 and study completion.

3) Proportion of patients experiencing an acute or adenosine provoking PV reconnection during the index ablation procedure will be analyzed using Chi-Square tests to assess group differences. Additional information per vein will be provided as well.

4) Proportion of patients who needed repeat ablation procedure because of documented recurrence of symptomatic AF/AFL/AT between index ablation and study completion will be analyzed using Chi-Square tests to assess group differences.

5) Proportion of patients who needed antiarrhythmic drug because of documented recurrence of symptomatic AF/AFL/AT will be displayed at phone contact and for the period between phone contact and Day 91. This proportion will be further displayed and analyzed using Chi-Square tests to assess group differences for the following periods: between Day 91 and Day 180, between Day 181 and study completion and between Day 91 and study completion.

6) Proportion of patients with any AF/AFL/AT during the first 90 days post ablation will be analyzed using Chi-Square tests to assess group differences. In addition, the proportion will also be

summarized and compared with chi-square tests across the three groups for the following periods: Day 91 to Day 180 (inclusive) and Day 181 to study completion (inclusive). Number of episodes of any AF/AFL/AT during these three time periods will also be provided and compared, if appropriate, between groups using Poisson regression models. The same analyses will be repeated for proportion of patients with symptomatic AF/AFL/AT.

7) Proportion of patients with emergency visit or hospitalization in a health-care facility will be analyzed using Chi-Square tests to assess group differences (both individually and as a composite endpoint) for the following periods: between discharge and Day 90, between Day 91 and Day 180, between Day 181 and study completion and between Day 91 and study completion. In addition, proportion of patients requiring a cardioversion will be similarly presented.

8) Proportion of patients with major complications including death, stroke, TIA, Myocardial Infarction or systemic thromboembolism, PV stenosis, phrenic nerve palsy, pericarditis, pericardial effusion, cardiac perforation or tamponade, hematoma, AV fistula, pseudoaneurysm, esophageal injury and atrio-esophageal fistulae (both individually and as a composite endpoint) and acute peri-procedural complications will be analyzed using Chi-Square tests to assess group differences. This will be done for the following periods: prior to discharge (inclusive) and between discharge and study completion. The proportion of patients with major complications before discharge and between discharge and study completion will also be presented for the implanted population. Acute peri-procedural (from index ablation to 30 days post ablation) and delayed (from 31 days post ablation and study completion) complications will also be presented and compared using Chi-Square tests.

9) Health-related QoL scores (EQ-5D-3L score and overall AFEQT score) assessed at baseline, 6 months and 12 months will be analyzed using a repeated measures analysis of covariance, adjusting for baseline values to reduce the error mean squares. In the event of missing data, a multiple imputation approach using SAS procedures PROC MI and PROC MIANALYZE will be considered, if necessary. Only descriptive statistics will be provided for the CCS-SAF score assessed at baseline.

10) Single and multiple procedure success defined as freedom from <u>symptomatic or asymptomatic</u> electrocardiographically documented AF/AFL/AT after the first and last ablation procedure respectively will be analyzed as the primary endpoint using Kaplan-Meier survival curves and log-rank tests.

11) Single and multiple procedure success defined as freedom from <u>symptomatic</u> electrocardiographically documented AF/AFL/AT after the first and last ablation procedure respectively will be analyzed as the primary endpoint using Kaplan-Meier survival curves and log-rank tests.

In addition to the analyses of the secondary endpoints above, the primary endpoint and the first secondary endpoint will be further explored as described below. The proportion of patients experiencing each component of the primary and first secondary endpoint:

- Any AF (symptomatic or asymptomatic) between Day 91 and study completion;
- Any AFL/AT (symptomatic or asymptomatic) between Day 91 and study completion;
- Any AF/AFL/AT (symptomatic or asymptomatic) between Day 91 and study completion;
- Symptomatic AF between Day 91 and study completion;
- Symptomatic AFL/AT between Day 91 and study completion;
- Symptomatic AF/AFL/AT between Day 91 and study completion;
- Repeat procedure including during the first 90 days (i.e. between index ablation and study completion)

will be displayed as well and compared between the three groups (RFCA, CRYO 4 and CRYO 2) using chi-square tests on the ITT population. Number of episodes of any AF/AFL/AT (symptomatic or asymptomatic) and number of episodes of symptomatic AF/AFL/AT between Day 91 and study completion will also be provided and compared across groups using a Poisson regression model. Modeling will be done with the SAS GENMOD procedure and the logarithm of the time of follow-up (study completion date – Day 91 + 1) will be used as the offset. It should be noted that the distribution of the number of episodes will be reviewed prior to hardlock and other statistical models may be used if deemed more appropriate.

7.3.3 Other Pre Specified Analyses

A Cox proportional hazards model will be used to test the consistency of the group effect for the primary endpoint while accounting for clinically important baseline characteristics. The adjusted hazard ratios will be presented for the group effect with confidence intervals. Important baseline characteristics will include:

- Site,
- Age (age at inform consent, CRF p.2);
- Gender (CRF p.2);
- Race (CRF p.2);
- Weight (CRF p.9);
- LA size (left atrial AP diameter, CRF p.12);
- Structural heart disease (LVEF <40%, CRF p.12);
- AF duration (year of informed consent (CRF p.2) - year of medical diagnosis (CRF p.7));
- Number of different AADs used in the past (CRF p.8).

The proportional hazard assumption will be assessed by visual inspection of the log-negative-log plot and through a formal test of the interaction term “group x time” at α=0.05. Should this assumption fail, a stratified Cox model will be fitted in order to correct for non-proportional hazards if possible or, if ineffective, time-dependent variables will be introduced. Should these corrective techniques fail; logistic regression will be used instead.

Subgroup analyses to investigate heterogeneity in group effect for the primary endpoint will be performed using Cox proportional hazards models including terms for the factor defining the subgroups, the group and the factor-by-group interaction. The factor-by-group interaction will be tested and used

to determine the consistency of the group effect across subgroups. Subgroups based on the following variables will be considered:

- Ablation experience (high volume vs. low volume centers as defined by procedure volume above/below the median), (high volume: sites 001, 002, 003, and 005; low volume: sites 004, 006, 007, 008);
- LA size (above/below median), (CRF p.12);
- AF duration (above/below median), (AF duration computed as year of informed consent (CRF p.2) - year of medical diagnosis (CRF p.7));
- AF subtype (paroxysmal vs. early persistent), (CRF p.3);
- PV anatomy (common ostia (L1a, L1b, R3a, R3b, R3c, R4a, R4b, R5) vs. standard PV pattern (if no L1a, L1b, R3a, R3b, R3c, R4a, R4b, R5)), CRF p. 13).

7.4 Other Endpoints and Procedure Parameters Analysis

Unless otherwise stated, analysis will be presented for the ITT population.

7.4.1 Atrial Arrhythmia Assessment

Occurrence of any documented episode(s) of atrial arrhythmia (Yes/No) between index ablation and phone contact, occurrence of symptomatic episode(s) of atrial arrhythmia (Yes/No) between phone contact and 3 months (inclusive), between 3 and 6 months (inclusive) as well as between 6 months and study completion will be presented using counts and proportions, overall and by treatment strategy group. Related information (number of episode(s), symptoms and severity of the worst symptoms) will be summarized using descriptive statistics, overall and by treatment strategy group. Listings may also be presented as appropriate.

7.4.2 Implantable Loop Recorder (ILR)

Parameters related to the ILR will be summarized using descriptive statistics overall and by treatment strategy group. Information will be available at visit implantation, ablation, 3 months, 6 months and 12 months. Summary of the ILR at implantation will also be displayed for the implanted population.

7.4.3 Ablation Procedure Parameters

The parameters collected during the index ablation procedure and during additional ablation procedures will be summarized using descriptive statistics overall and by treatment strategy group. Listings may also be presented as appropriate.

The procedure used and presenting rhythm for each additional ablation procedure will be presented for all repeat ablation procedure. All other parameters will be presented only for the first additional ablation procedure.

Information captured during the radiofrequency ablation, cryoballoon ablation and phrenic monitoring will be summarized for each pulmonary vein using counts and proportions, overall and by treatment strategy group.

Similarly, PV reconnection information will be presented for each pulmonary vein using counts and proportions, overall and by treatment strategy group. Finally, sites where pulmonary vein reconnection will be observed post PVI (SA, S, SP, Posterior, IP, I, IA, Anterior) will be summarized for each pulmonary vein using counts, overall and by treatment strategy group.

Several tables in this section will present information broken down by pulmonary vein. To get a more global picture, information related to the right veins (RSPV, RIPV, RCPV and RMPV), the left veins (LSPV, LIPV, LCPV and LMVP) and all the veins, will be combined and summarized as defined above. However, in the corresponding tables, counts will refer to number of veins instead of number of patients.

Finally, duration of the procedure and fluoroscopy time, captured during the index ablation procedure, will be presented overall and by treatment strategy group. Although not considered as secondary endpoints per se in the protocol, these parameters will be compared between groups using ANOVA models. Pairwise comparisons will be done using contrasts under the ANOVA models.

7.5 Safety Parameters Analysis

Unless otherwise stated, analysis will be presented for the ITT population.

7.5.1 Concomitant Medications

Concomitant medications will be collected throughout the study, starting at inclusion (study entry) and will be coded with respect to indication and generic name using the WHO drug dictionary (version March 2014). Frequency of use of concomitant medications will be presented by both indication and generic name, overall and by treatment strategy group.

A listing of concomitant medications will also be provided.

7.5.2 Adverse Events and Serious Adverse Events

AEs recorded in the CRF will be coded by system organ class and body system according to the MedDRA dictionary (version 17). AEs and SAEs recorded in the CRF will be listed. A listing and an Excel file will also be generated for SAEs adjudicated by the adjudication committee.

7.5.3 Vital Signs, 12-Lead ECG and Physical Exam Analysis

Parameters related to the various exams will be summarized using descriptive statistics and presented overall and by treatment strategy group at each available visit. Listings may also be presented as appropriate.

7.5.4 Other Exam Analysis

Parameters related to the various exams will be summarized using descriptive statistics and presented overall and by treatment strategy group at each available visit. Listings may also be presented as appropriate.

7.5.5 Laboratory Parameters

Laboratory parameters (potassium, hemoglobin, creatinine and eGFR) measured before the ablation will be summarized using descriptive statistics overall and by treatment strategy group. Creatinine will be reported in μmol/L or mg/dL in the eCRF but will be summarized in μmol/L. The following conversion formula will be used:

$$Y\ \mu mol/L = 88.4 \cdot X\ mg/dL$$

7.5.6 Childbearing Potential

Childbearing potential will be summarized using descriptive statistics overall and by treatment strategy group.

7 SUMMARY TABLES, FIGURES AND LISTINGS

1 Study subject	
1.1 Subject disposition	
Table 1.1.1	*Subject disposition, ITT population*
Table 1.1.2	*Subject disposition by study site, ITT population*
Table 1.1.3	*Data sets analyzed – ITT and Evaluable populations*
Table 1.1.4	*Data sets analyzed – Implanted population*
1.2 Demographic and baseline characteristics	
Table 1.2.1	*Summary of demographics, ITT population*
Table 1.2.2	*Summary of demographics, Implanted population*
Table 1.2.3	*Summary of medical history, ITT population*
Table 1.2.4	*Summary of medical history, Implanted population*
Table 1.2.5	*Summary of atrial fibrillation history, ITT population*
Table 1.2.6	*Summary of atrial fibrillation history, Implanted population*
1.3 ILR compliance	
Table 1.3.1	*Summary of ILR compliance – Between ablation and study completion, ITT population*
Table 1.3.2	*Summary of ILR compliance – Between ablation and study completion, Evaluable population*
Table 1.3.3	*Summary of ILR compliance – Between ablation and Day 90, ITT population*
Table 1.3.4	*Summary of ILR compliance – Between ablation and Day 90, Evaluable population*
Table 1.3.5	*Summary of ILR compliance – Between Day 91 and study completion, ITT population*
Table 1.3.6	*Summary of ILR compliance – Between Day 91 and study completion, Evaluable population*

2 Efficacy analysis	
2.1 Primary analysis	
Analysis of the primary endpoint	
Table 2.1.1	*Summary and statistical analysis of first recurrence of any AF/AFL/AT (symptomatic or asymptomatic) between Day 91 and study completion OR any repeat ablation procedure including during the first 90 days, ITT population*
Table 2.1.2	*Summary and statistical analysis of first recurrence of any AF/AFL/AT (symptomatic or asymptomatic) between Day 91 and study completion OR any repeat ablation procedure including during the first 90 days, Evaluable population*
2.2 Secondary analysis	
Analysis of the first secondary endpoint	
Table 2.2.1	*Summary and statistical analysis of first recurrence of symptomatic AF/AFL/AT between Day 91 and study completion OR any repeat ablation procedure including during the first 90 days, ITT population*
Table 2.2.2	*Summary and statistical analysis of first recurrence of symptomatic AF/AFL/AT between Day 91 and study completion OR any repeat ablation procedure including during the first 90 days, Evaluable population*
Analysis of the components of the primary and first secondary endpoint	
Table 2.2.3	*Summary and statistical analysis of proportion of patients with any AF (symptomatic or asymptomatic) between Day 91 and study completion, ITT population*
Table 2.2.4	*Summary and statistical analysis of proportion of patients with any AFL/AT (symptomatic or asymptomatic) between Day 91 and study completion, ITT population*
Table 2.2.5	*Summary and statistical analysis of proportion of patients with any AF/AFL/AT (symptomatic or asymptomatic) between Day 91 and study completion, ITT population*
Table 2.2.6	*Summary and statistical analysis of number of episodes of any AF/AFL/AT (symptomatic or asymptomatic) between Day 91 and study completion, ITT population*
Table 2.2.7	*Summary and statistical analysis of proportion of patients with symptomatic AF between Day 91 and study completion, ITT population*
Table 2.2.8	*Summary and statistical analysis of proportion of patients with symptomatic AFL/AT between Day 91 and study completion, ITT population*
Table 2.2.9	*Summary and statistical analysis of proportion of patients with symptomatic AF/AFL/AT between Day 91 and study completion, ITT population*
Table 2.2.10	*Summary and statistical analysis of number of episodes of symptomatic AF/AFL/AT between Day 91 and study completion, ITT population*
Table 2.2.11	*Summary and statistical analysis of proportion of patients with any repeat ablation procedure including during the first 90 days, ITT population*

Analysis of AF/AFL/AT and antiarrhythmic drug use by time period	
Table 2.2.12	*Summary and statistical analysis of proportion of patients with any AF/AFL/AT (symptomatic or asymptomatic) between ablation and Day 90, ITT population*
Table 2.2.13	*Summary and statistical analysis of proportion of patients with symptomatic AF/AFL/AT between ablation and Day 90, ITT population*
Table 2.2.14	*Summary and statistical analysis of number of episodes of any AF/AFL/AT (symptomatic or asymptomatic) between ablation and Day 90, ITT population*
Table 2.2.15	*Summary and statistical analysis of number of episodes of symptomatic AF/AFL/AT between ablation and Day 90, ITT population*
Table 2.2.16	*Summary and statistical analysis of proportion of patients with any AF/AFL/AT (symptomatic or asymptomatic) between Day 91 and Day 180, ITT population*
Table 2.2.17	*Summary and statistical analysis of proportion of patients with symptomatic AF/AFL/AT between Day 91 and Day 180, ITT population*
Table 2.2.18	*Summary and statistical analysis of number of episodes of any AF/AFL/AT (symptomatic or asymptomatic) between Day 91 and Day 180, ITT population*
Table 2.2.19	*Summary and statistical analysis of number of episodes of symptomatic AF/AFL/AT between Day 91 and Day 180, ITT population*
Table 2.2.20	*Summary and statistical analysis of proportion of patients with any AF/AFL/AT (symptomatic or asymptomatic) between Day 181 and study completion, ITT population*
Table 2.2.21	*Summary and statistical analysis of proportion of patients with symptomatic AF/AFL/AT between Day 181 and study completion, ITT population*
Table 2.2.22	*Summary and statistical analysis of number of episodes of any AF/AFL/AT (symptomatic or asymptomatic) between Day 181 and study completion, ITT population*
Table 2.2.23	*Summary and statistical analysis of number of episodes of symptomatic AF/AFL/AT between Day 181 and study completion, ITT population*
Table 2.2.24	*Summary of proportion of patients with antiarrhythmic drug use before Day 91, ITT population*
Table 2.2.25	*Summary and statistical analysis of proportion of patients with antiarrhythmic drug use between Day 91 and Day 180, ITT population*
Table 2.2.26	*Summary and statistical analysis of proportion of patients with antiarrhythmic drug use between Day 181 and study completion, ITT population*
Table 2.2.27	*Summary and statistical analysis of proportion of patients with antiarrhythmic drug use between Day 91 and study completion, ITT population*

Analysis of AF/AFL/AT after the first and last ablation	
Table 2.2.28	*Summary and statistical analysis of first recurrence of any AF/AFL/AT (symptomatic or asymptomatic) between index ablation and study completion, ITT population*
Table 2.2.29	*Summary and statistical analysis of first recurrence of any AF/AFL/AT (symptomatic or asymptomatic) between last ablation and study completion, ITT population*
Table 2.2.30	*Summary and statistical analysis of first recurrence of symptomatic AF/AFL/AT between index ablation and study completion, ITT population*
Table 2.2.31	*Summary and statistical analysis of first recurrence of symptomatic AF/AFL/AT between last ablation and study completion, ITT population*

Analysis of arrhythmia burden	
Table 2.2.32	*Summary and statistical analysis of daily AF burden (hours/day), ITT population*
Table 2.2.33	*Summary and statistical analysis of overall AF burden (% time in AF), ITT population*

Analysis of the other secondary endpoints	
Table 2.2.34	*Summary and statistical analysis of proportion of patients with repeat ablation procedures because of documented symptomatic AF/AFL/AT between ablation and study completion, ITT population*
Table 2.2.35	*Summary and statistical analysis of proportion of patients with emergency visits between Discharge and Day 90, ITT population*
Table 2.2.36	*Summary and statistical analysis of proportion of patients with emergency visits between Day 91 and Day 180, ITT population*
Table 2.2.37	*Summary and statistical analysis of proportion of patients with emergency visits between Day 181 and study completion, ITT population*
Table 2.2.38	*Summary and statistical analysis of proportion of patients with emergency visits between Day 91 and study completion, ITT population*
Table 2.2.39	*Summary and statistical analysis of proportion of patients with hospitalizations between Discharge and Day 90, ITT population*
Table 2.2.40	*Summary and statistical analysis of proportion of patients with hospitalizations between Day 91 and Day 180, ITT population*
Table 2.2.41	*Summary and statistical analysis of proportion of patients with hospitalizations between Day 181 and study completion, ITT population*
Table 2.2.42	*Summary and statistical analysis of proportion of patients with hospitalizations between Day 91 and study completion, ITT population*
Table 2.2.43	*Summary and statistical analysis of proportion of patients with emergency visits and/or hospitalizations between Discharge and Day 90, ITT population*
Table 2.2.44	*Summary and statistical analysis of proportion of patients with emergency visits and/or hospitalizations between Day 91 and Day 180, ITT population*
Table 2.2.45	*Summary and statistical analysis of proportion of patients with emergency visits and/or hospitalizations between Day 181 and study completion, ITT population*
Table 2.2.46	*Summary and statistical analysis of proportion of patients with emergency visits and/or hospitalizations between Day 91 and study completion, ITT population*
Table 2.2.47	*Summary of cardioversions between Discharge and Day 90, ITT population*
Table 2.2.48	*Summary of cardioversions between Day 91 and Day 180, ITT population*
Table 2.2.49	*Summary of cardioversions between Day 181 and study completion, ITT population*
Table 2.2.50	*Summary of cardioversions between Day 91 and study completion, ITT population*
Table 2.2.51	*Summary and statistical analysis of major complications as a composite endpoint, <= Discharge, ITT population*
Table 2.2.52	*Summary of the components of the major complications, <= Discharge, ITT population*
Table 2.2.53	*Summary and statistical analysis of major complications as a composite endpoint between Discharge and study completion, ITT population*
Table 2.2.54	*Summary of the components of the major complications between Discharge and study completion, ITT population*
Table 2.2.55	*Summary and statistical analysis of major complications as a composite endpoint, <= Discharge, Implanted population*
Table 2.2.56	*Summary of the components of the major complications, <= Discharge, Implanted population*
Table 2.2.57	*Summary and statistical analysis of major complications as a composite endpoint between Discharge and study completion, Implanted population*

Table 2.2.58	*Summary of the components of the major complications between Discharge and study completion, Implanted population*
Table 2.2.59	*Summary and statistical analysis of acute peri-procedural complications as a composite endpoint between ablation and 30 days post ablation (inclusive), ITT population*
Table 2.2.60	*Summary of the components of the acute peri-procedural complications between ablation and 30 days post ablation (inclusive), ITT population*
Table 2.2.61	*Summary and statistical analysis of delayed complications as a composite endpoint between 31 days post ablation (inclusive) and study completion, ITT population*
Table 2.2.62	*Summary of the components of delayed complications between 31 days post ablation (inclusive) and study completion, ITT population*
Table 2.2.63	*Summary and statistical analysis of patients experiencing an acute or adenosine provoking PV reconnection during the index ablation procedure, ITT population*
Table 2.2.64	*Summary of PV reconnection during the index ablation procedure, VEIN=RSPV, ITT population*
Table 2.2.65	*Summary of PV reconnection during the index ablation procedure, VEIN=RIPV, ITT population*
Table 2.2.66	*Summary of PV reconnection during the index ablation procedure, VEIN=RCPV, ITT population*
Table 2.2.67	*Summary of PV reconnection during the index ablation procedure, VEIN=RMPV, ITT population*
Table 2.2.68	*Summary of PV reconnection during the index ablation procedure, VEIN=LSPV, ITT population*
Table 2.2.69	*Summary of PV reconnection during the index ablation procedure, VEIN=LIPV, ITT population*
Table 2.2.70	*Summary of PV reconnection during the index ablation procedure, VEIN=LCPV, ITT population*
Table 2.2.71	*Summary of PV reconnection during the index ablation procedure, VEIN=LMPV, ITT population*
Table 2.2.72	*Summary of PV reconnection during the index ablation procedure, LEFT VEINS, ITT population*
Table 2.2.73	*Summary of PV reconnection during the index ablation procedure, RIGHT VEINS, ITT population*
Table 2.2.74	*Summary of PV reconnection during the index ablation procedure, ALL VEINS, ITT population*
Table 2.2.75	*Summary of PV reconnection sites during the index ablation procedure, VEIN=RSPV, ITT population*
Table 2.2.76	*Summary of PV reconnection sites during the index ablation procedure, VEIN=RIPV, ITT population*
Table 2.2.77	*Summary of PV reconnection sites during the index ablation procedure, VEIN=RCPV, ITT population*
Table 2.2.78	*Summary of PV reconnection sites during the index ablation procedure, VEIN=RMPV, ITT population*
Table 2.2.79	*Summary of PV reconnection sites during the index ablation procedure, VEIN=LSPV, ITT population*
Table 2.2.80	*Summary of PV reconnection sites during the index ablation procedure, VEIN=LIPV, ITT population*
Table 2.2.81	*Summary of PV reconnection sites during the index ablation procedure, VEIN=LCPV, ITT population*
Table 2.2.82	*Summary of PV reconnection sites during the index ablation procedure, VEIN=LMPV, ITT population*
Table 2.2.83	*Summary of PV reconnection sites during the index ablation procedure, LEFT VEINS, ITT population*
Table 2.2.84	*Summary of PV reconnection sites during the index ablation procedure, RIGHT VEINS, ITT population*
Table 2.2.85	*Summary of PV reconnection sites during the index ablation procedure, ALL VEINS, ITT population*
Table 2.2.86	*Summary of Health-related QoL scores (CCS-SAF score), ITT population*
Table 2.2.87	*Summary and statistical analysis of Health-related QoL scores (EQ-5D-3L score), ITT population*
Table 2.2.88	*Summary and statistical analysis of Health-related QoL scores (overall AFEQT score), ITT*

	population

2.3 Other pre specified analyses	
Analysis of the primary endpoint	
Table 2.3.1	*Summary and multiple Cox regression model of first recurrence of any AF/AFL/AT (symptomatic or asymptomatic) between Day 91 and study completion OR any repeat ablation procedure including during the first 90 days, ITT population*
Table 2.3.2	*First recurrence of any AF/AFL/AT (symptomatic or asymptomatic) between Day 91 and study completion OR any repeat ablation procedure including during the first 90 days broken down by subgroup variable (Ablation experience), ITT population*
Table 2.3.3	*First recurrence of any AF/AFL/AT (symptomatic or asymptomatic) between Day 91 and study completion OR any repeat ablation procedure including during the first 90 days broken down by subgroup variable (LA size), ITT population*
Table 2.3.4	*First recurrence of any AF/AFL/AT (symptomatic or asymptomatic) between Day 91 and study completion OR any repeat ablation procedure including during the first 90 days broken down by subgroup variable (AF duration), ITT population*
Table 2.3.5	*First recurrence of any AF/AFL/AT (symptomatic or asymptomatic) between Day 91 and study completion OR any repeat ablation procedure including during the first 90 days broken down by subgroup variable (AF subtype), ITT population*
Table 2.3.6	*First recurrence of any AF/AFL/AT (symptomatic or asymptomatic) between Day 91 and study completion OR any repeat ablation procedure including during the first 90 days broken down by subgroup variable (PV anatomy), ITT population*

3 Other endpoints and procedure parameters	
3.1 Atrial arrhythmia assessment	
Table 3.1.1	*Summary of proportion of patients reporting at least one episode of atrial arrhythmia since last visit at either visit 3 months, visit 6 months or visit 12 months, ITT population*
Table 3.1.2	*Summary of proportion of patients reporting any documented episode(s) of atrial arrhythmia since last visit at phone contact, ITT population*
Table 3.1.3	*Summary of proportion of patients reporting any symptomatic episode(s) of atrial arrhythmia since last visit at visit 3 months, ITT population*
Table 3.1.4	*Summary of related atrial arrhythmia information: number of episodes, symptoms and severity of the worst symptom at visit 3 months, ITT population*
Table 3.1.5	*Summary of proportion of patients reporting any symptomatic episode(s) of atrial arrhythmia since last visit at visit 6 months, ITT population*
Table 3.1.6	*Summary of related atrial arrhythmia information: number of episodes, symptoms and severity of the worst symptom at visit 6 months, ITT population*
Table 3.1.7	*Summary of proportion of patients reporting any symptomatic episode(s) of atrial arrhythmia since last visit at visit 12 months, ITT population*
Table 3.1.8	*Summary of related atrial arrhythmia information: number of episodes, symptoms and severity of the worst symptom at visit 12 months, ITT population*

3.2 Implantable loop recorder	
Table 3.2.1	*Summary of ILR implantation, ITT population*
Table 3.2.2	*Summary of ILR implantation, Implanted population*
Table 3.2.3	*Summary of ILR at time of ablation, ITT population*
Table 3.2.4	*Summary of ILR at visit 3 months, ITT population*
Table 3.2.5	*Summary of ILR at visit 6 months, ITT population*
Table 3.2.6	*Summary of ILR at visit 12 months, ITT population*

3.3 Index ablation procedure parameters	
Table 3.3.1	*Summary of rhythm before start of index procedure ablation, ITT population*
Table 3.3.2	*Summary of index procedure summary, ITT population*
Table 3.3.3	*Summary of anticoagulation therapy – index procedure, ITT population*
Table 3.3.4	*Summary of antiarrhythmic drug – index procedure, ITT population*
Table 3.3.5	*Summary of esophageal temperature – index procedure, ITT population*
Table 3.3.6	*Summary of radiofrequency ablation (RFCA) – index procedure – VEIN=RSPV, ITT population*
Table 3.3.7	*Summary of radiofrequency ablation (RFCA) – index procedure – VEIN=RIPV, ITT population*
Table 3.3.8	*Summary of radiofrequency ablation (RFCA) – index procedure – VEIN=RCPV, ITT population*
Table 3.3.9	*Summary of radiofrequency ablation (RFCA) – index procedure – VEIN=RMPV, ITT population*
Table 3.3.10	*Summary of radiofrequency ablation (RFCA) – index procedure – VEIN=LSPV, ITT population*
Table 3.3.11	*Summary of radiofrequency ablation (RFCA) – index procedure – VEIN=LIPV, ITT population*
Table 3.3.12	*Summary of radiofrequency ablation (RFCA) – index procedure – VEIN=LCPV, ITT population*
Table 3.3.13	*Summary of radiofrequency ablation (RFCA) – index procedure – VEIN=LMPV, ITT population*
Table 3.3.14	*Summary of radiofrequency ablation (RFCA) – index procedure – LEFT VEINS, ITT population*
Table 3.3.15	*Summary of radiofrequency ablation (RFCA) – index procedure – RIGHT VEINS, ITT population*
Table 3.3.16	*Summary of radiofrequency ablation (RFCA) – index procedure – ALL VEINS, ITT population*
Table 3.3.17	*Summary of maximum radiofrequency energy used (RFCA) – index procedure, ITT population*
Table 3.3.18	*Summary of cryoballoon ablation (CRYO) – index procedure – VEIN=RSPV, ITT population*
Table 3.3.19	*Summary of cryoballoon ablation (CRYO) – index procedure – VEIN=RIPV, ITT population*

Table 3.3.20	*Summary of cryoballoon ablation (CRYO) – index procedure – VEIN=RCPV, ITT population*
Table 3.3.21	*Summary of cryoballoon ablation (CRYO) – index procedure – VEIN=RMPV, ITT population*
Table 3.3.22	*Summary of cryoballoon ablation (CRYO) – index procedure – VEIN=LSPV, ITT population*
Table 3.3.23	*Summary of cryoballoon ablation (CRYO) – index procedure – VEIN=LIPV, ITT population*
Table 3.3.24	*Summary of cryoballoon ablation (CRYO) – index procedure – VEIN=LCPV, ITT population*
Table 3.3.25	*Summary of cryoballoon ablation (CRYO) – index procedure – VEIN=LMPV, ITT population*
Table 3.3.26	*Summary of cryoballoon ablation (CRYO) – index procedure – LEFT VEINS, ITT population*
Table 3.3.27	*Summary of cryoballoon ablation (CRYO) – index procedure – RIGHT VEINS, ITT population*
Table 3.3.28	*Summary of cryoballoon ablation (CRYO) – index procedure – ALL VEINS, ITT population*
Table 3.3.29	*Summary of phrenic monitoring (CRYO) – index procedure, ITT population*
Table 3.3.30	*Summary of phrenic monitoring (CRYO) – index procedure – VEIN=RSPV, ITT population*
Table 3.3.31	*Summary of phrenic monitoring (CRYO) – index procedure – VEIN=RIPV, ITT population*
Table 3.3.32	*Summary of phrenic monitoring (CRYO) – index procedure – VEIN=RCPV, ITT population*
Table 3.3.33	*Summary of phrenic monitoring (CRYO) – index procedure – VEIN=RMPV, ITT population*
Table 3.3.34	*Summary of phrenic monitoring (CRYO) – index procedure – VEIN=LSPV, ITT population*
Table 3.3.35	*Summary of phrenic monitoring (CRYO) – index procedure – VEIN=LIPV, ITT population*
Table 3.3.36	*Summary of phrenic monitoring (CRYO) – index procedure – VEIN=LCPV, ITT population*
Table 3.3.37	*Summary of phrenic monitoring (CRYO) – index procedure – VEIN=LMPV, ITT population*
Table 3.3.38	*Summary of phrenic monitoring (CRYO) – index procedure – LEFT VEINS, ITT population*
Table 3.3.39	*Summary of phrenic monitoring (CRYO) – index procedure – RIGHT VEINS, ITT population*
Table 3.3.40	*Summary of phrenic monitoring (CRYO) – index procedure – ALL VEINS, ITT population*
Table 3.3.41	*Summary and statistical analysis of procedure duration for PVI of index ablation, ITT population*
Table 3.3.42	*Summary and statistical analysis of fluoroscopy duration for PVI of index ablation, ITT population*

3.4 Repeat ablation procedure parameters	
Table 3.4.1	*Summary of all repeat ablation procedure, ITT population*
Table 3.4.2	*Summary of first repeat ablation procedure, ITT population*
Table 3.4.3	*Summary of repeat ablation procedure summary, ITT population*
Table 3.4.4	*Summary of anticoagulation therapy – repeat ablation, ITT population*
Table 3.4.5	*Summary of antiarrhythmic drug – repeat ablation, ITT population*
Table 3.4.6	*Summary of esophageal temperature – repeat ablation, ITT population*
Table 3.4.7	*Summary of radiofrequency ablation (RFCA) – repeat ablation – VEIN=RSPV, ITT population*
Table 3.4.8	*Summary of radiofrequency ablation (RFCA) – repeat ablation – VEIN=RIPV, ITT population*
Table 3.4.9	*Summary of radiofrequency ablation (RFCA) – repeat ablation – VEIN=RCPV, ITT population*
Table 3.4.10	*Summary of radiofrequency ablation (RFCA) – repeat ablation – VEIN=RMPV, ITT population*
Table 3.4.11	*Summary of radiofrequency ablation (RFCA) – repeat ablation – VEIN=LSPV, ITT population*
Table 3.4.12	*Summary of radiofrequency ablation (RFCA) – repeat ablation – VEIN=LIPV, ITT population*
Table 3.4.13	*Summary of radiofrequency ablation (RFCA) – repeat ablation – VEIN=LCPV, ITT population*
Table 3.4.14	*Summary of radiofrequency ablation (RFCA) – repeat ablation – VEIN=LMPV, ITT population*
Table 3.4.15	*Summary of radiofrequency ablation (RFCA) – repeat ablation – LEFT VEINS, ITT population*
Table 3.4.16	*Summary of radiofrequency ablation (RFCA) – repeat ablation – RIGHT VEINS, ITT population*
Table 3.4.17	*Summary of radiofrequency ablation (RFCA) – repeat ablation – ALL VEINS, ITT population*
Table 3.4.18	*Summary of maximum radiofrequency energy used (RFCA) – repeat ablation, ITT population*
Table 3.4.19	*Summary of cryoballoon ablation (CRYO) – repeat ablation – VEIN=RSPV, ITT population*
Table 3.4.20	*Summary of cryoballoon ablation (CRYO) – repeat ablation – VEIN=RIPV, ITT population*
Table 3.4.21	*Summary of cryoballoon ablation (CRYO) – repeat ablation – VEIN=RCPV, ITT population*

Table 3.4.22	*Summary of cryoballoon ablation (CRYO) – repeat ablation – VEIN=RMPV, ITT population*
Table 3.4.23	*Summary of cryoballoon ablation (CRYO) – repeat ablation – VEIN=LSPV, ITT population*
Table 3.4.24	*Summary of cryoballoon ablation (CRYO) – repeat ablation – VEIN=LIPV, ITT population*
Table 3.4.25	*Summary of cryoballoon ablation (CRYO) – repeat ablation – VEIN=LCPV, ITT population*
Table 3.4.26	*Summary of cryoballoon ablation (CRYO) – repeat ablation – VEIN=LMPV, ITT population*
Table 3.4.27	*Summary of cryoballoon ablation (CRYO) – repeat ablation – LEFT VEINS, ITT population*
Table 3.4.28	*Summary of cryoballoon ablation (CRYO) – repeat ablation – RIGHT VEINS, ITT population*
Table 3.4.29	*Summary of cryoballoon ablation (CRYO) – repeat ablation – ALL VEINS, ITT population*
Table 3.4.30	*Summary of phrenic monitoring (CRYO) – repeat ablation, ITT population*
Table 3.4.31	*Summary of phrenic monitoring (CRYO) – repeat ablation – VEIN=RSPV, ITT population*
Table 3.4.32	*Summary of phrenic monitoring (CRYO) – repeat ablation – VEIN=RIPV, ITT population*
Table 3.4.33	*Summary of phrenic monitoring (CRYO) – repeat ablation – VEIN=RCPV, ITT population*
Table 3.4.34	*Summary of phrenic monitoring (CRYO) – repeat ablation – VEIN=RMPV, ITT population*
Table 3.4.35	*Summary of phrenic monitoring (CRYO) – repeat ablation – VEIN=LSPV, ITT population*
Table 3.4.36	*Summary of phrenic monitoring (CRYO) – repeat ablation – VEIN=LIPV, ITT population*
Table 3.4.37	*Summary of phrenic monitoring (CRYO) – repeat ablation – VEIN=LCPV, ITT population*
Table 3.4.38	*Summary of phrenic monitoring (CRYO) – repeat ablation – VEIN=LMPV, ITT population*
Table 3.4.39	*Summary of phrenic monitoring (CRYO) – repeat ablation – LEFT VEINS, ITT population*
Table 3.4.40	*Summary of phrenic monitoring (CRYO) – repeat ablation – RIGHT VEINS, ITT population*
Table 3.4.41	*Summary of phrenic monitoring (CRYO) – repeat ablation – ALL VEINS, ITT population*
Table 3.4.42	*Summary of patients experiencing an acute or adenosine provoking PV reconnection during the repeat ablation procedure, ITT population*
Table 3.4.43	*Summary of PV reconnection during the repeat ablation procedure, VEIN=RSPV, ITT population*
Table 3.4.44	*Summary of PV reconnection during the repeat ablation procedure, VEIN=RIPV, ITT population*
Table 3.4.45	*Summary of PV reconnection during the repeat ablation procedure, VEIN=RCPV, ITT population*
Table 3.4.46	*Summary of PV reconnection during the repeat ablation procedure, VEIN=RMPV, ITT population*
Table 3.4.47	*Summary of PV reconnection during the repeat ablation procedure, VEIN=LSPV, ITT population*
Table 3.4.48	*Summary of PV reconnection during the repeat ablation procedure, VEIN=LIPV, ITT population*
Table 3.4.49	*Summary of PV reconnection during the repeat ablation procedure, VEIN=LCPV, ITT population*
Table 3.4.50	*Summary of PV reconnection during the repeat ablation procedure, VEIN=LMPV, ITT population*
Table 3.4.51	*Summary of PV reconnection during the repeat ablation procedure, LEFT VEINS, ITT population*
Table 3.4.52	*Summary of PV reconnection during the repeat ablation procedure, RIGHT VEINS, ITT population*
Table 3.4.53	*Summary of PV reconnection during the repeat ablation procedure, ALL VEINS, ITT population*
Table 3.4.54	*Summary of PV reconnection sites during the repeat ablation procedure, VEIN=RSPV, ITT population*
Table 3.4.55	*Summary of PV reconnection sites during the repeat ablation procedure, VEIN=RIPV, ITT population*
Table 3.4.56	*Summary of PV reconnection sites during the repeat ablation procedure, VEIN=RCPV, ITT population*
Table 3.4.57	*Summary of PV reconnection sites during the repeat ablation procedure, VEIN=RMPV, ITT population*
Table 3.4.58	*Summary of PV reconnection sites during the repeat ablation procedure, VEIN=LSPV, ITT population*
Table 3.4.59	*Summary of PV reconnection sites during the repeat ablation procedure, VEIN=LIPV, ITT population*
Table 3.4.60	*Summary of PV reconnection sites during the repeat ablation procedure, VEIN=LCPV, ITT population*

Table 3.4.61	*Summary of PV reconnection sites during the repeat ablation procedure, VEIN=LMPV, ITT population*
Table 3.4.62	*Summary of PV reconnection sites during the repeat ablation procedure, LEFT VEINS, ITT population*
Table 3.4.63	*Summary of PV reconnection sites during the repeat ablation procedure, RIGHT VEINS, ITT population*
Table 3.4.64	*Summary of PV reconnection sites during the repeat ablation procedure, ALL VEINS, ITT population*
Table 3.4.65	*Summary of repeat ablation procedure and fluoroscopy durations for PVI, ITT population*

4 Safety analyses	
4.1 Concomitant medication	
Table 4.1.1	*Summary of concomitant medication, ITT population*

4.2 Vital signs, 12-lead ECG and physical exam parameters	
Table 4.2.1	*Summary of clinical assessment - vital signs at baseline, ITT population*
Table 4.2.2	*Summary of clinical assessment - vital signs at discharge, ITT population*
Table 4.2.3	*Summary of clinical assessment - vital signs at 3 months, ITT population*
Table 4.2.4	*Summary of clinical assessment - vital signs at 6 months, ITT population*
Table 4.2.5	*Summary of clinical assessment - vital signs at 12 months, ITT population*
Table 4.2.6	*Summary of 12-lead ECG at baseline, ITT population*
Table 4.2.7	*Summary of 12-lead ECG at discharge, ITT population*
Table 4.2.8	*Summary of 12-lead ECG at 3 months, ITT population*
Table 4.2.9	*Summary of 12-lead ECG at 6 months, ITT population*
Table 4.2.10	*Summary of 12-lead ECG at 12 months, ITT population*
Table 4.2.11	*Summary of physical examination at baseline, ITT population*
Table 4.2.12	*Summary of physical examination at 3 months, ITT population*
Table 4.2.13	*Summary of physical examination at 6 months, ITT population*
Table 4.2.14	*Summary of physical examination at 12 months, ITT population*
Table 4.2.15	*Summary of NYHA functional class, ITT population*

4.3 Other exam parameters	
Table 4.3.1	*Summary of 24 hours Holter monitor exam at baseline, ITT population*
Table 4.3.2	*Summary of 24 hours Holter monitor exam at 3 months, ITT population*
Table 4.3.3	*Summary of 24 hours Holter monitor exam at 6 months, ITT population*
Table 4.3.4	*Summary of 24 hours Holter monitor exam at 12 months, ITT population*
Table 4.3.5	*Summary of transthoracic echocardiogram exam at baseline, ITT population*
Table 4.3.6	*Summary of CT / MRI exam at baseline, ITT population*
Table 4.3.7	*Summary of CT / MRI exam at 3 months, ITT population*
Table 4.3.8	*Summary of CT / MRI exam at 6 months, ITT population*
Table 4.3.9	*Summary of CT / MRI exam at 12 months, ITT population*
Table 4.3.10	*Summary of PV branching pattern at baseline, ITT population*
Table 4.3.11	*Summary of PV dimensions at baseline – VEIN=RSPV, ITT population*
Table 4.3.12	*Summary of PV dimensions at baseline – VEIN=RIPV, ITT population*
Table 4.3.13	*Summary of PV dimensions at baseline – VEIN=RCPV, ITT population*
Table 4.3.14	*Summary of PV dimensions at baseline – VEIN=RMPV, ITT population*
Table 4.3.15	*Summary of PV dimensions at baseline – VEIN=LSPV, ITT population*

Table 4.3.16	*Summary of PV dimensions at baseline – VEIN=LIPV, ITT population*
Table 4.3.17	*Summary of PV dimensions at baseline – VEIN=LCPV, ITT population*
Table 4.3.18	*Summary of PV dimensions at baseline – VEIN=LMPV, ITT population*
Table 4.3.19	*Summary of PV dimensions at baseline – LEFT VEINS, ITT population*
Table 4.3.20	*Summary of PV dimensions at baseline – RIGHT VEINS, ITT population*
Table 4.3.21	*Summary of PV dimensions at baseline – ALL VEINS, ITT population*

4.4 Laboratory parameters	
Table 4.4.1	*Summary of laboratory results at pre ablation procedure, ITT population*

4.5 Childbearing potential test	
Table 4.5.1	*Summary of pregnancy test before the ablation, ITT population*

Figures	
Figure 1	*Subject Disposition (flow chart)*
Figure 2	*Kaplan-Meier survival curves for time to first recurrence of any AF/AFL/AT (symptomatic or asymptomatic) between Day 91 and study completion OR any repeat ablation procedure including during the first 90 days, ITT population*
Figure 3	*Kaplan-Meier survival curves for time to first recurrence of any AF/AFL/AT (symptomatic or asymptomatic) between Day 91 and study completion OR any repeat ablation procedure including during the first 90 days, Evaluable population*
Figure 4	*Kaplan-Meier survival curves for time to first recurrence of symptomatic AF/AFL/AT between Day 91 and study completion OR any repeat ablation procedure including during the first 90 days, ITT population*
Figure 5	*Kaplan-Meier survival curves for time to first recurrence of symptomatic AF/AFL/AT between Day 91 and study completion OR any repeat ablation procedure including during the first 90 days, Evaluable population*
Figure 6	*Kaplan-Meier survival curves for time to first recurrence of any AF/AFL/AT (symptomatic or asymptomatic) between index ablation and study completion, ITT population*
Figure 7	*Kaplan-Meier survival curves for time to first recurrence of any AF/AFL/AT (symptomatic or asymptomatic) between last ablation and study completion, ITT population*
Figure 8	*Kaplan-Meier survival curves for time to first recurrence of symptomatic AF/AFL/AT between index ablation and study completion, ITT population*
Figure 9	*Kaplan-Meier survival curves for time to first recurrence of symptomatic AF/AFL/AT between last ablation and study completion, ITT population*

Listings	
Listing 1	*Subject disposition*
Listing 2	*Datasets Analyzed*
Listing 3	*Concomitant medications*
Listing 4	*Adverse events according to CRF*
Listing 5	*Serious adverse events according to CRF*
Listing 6	*Serious adverse events according to adjudication*
Listing 7	*Serious adverse events log – (Excel file)*
Listing 8	*Deaths*

8 References

1. Dorian P, Cvitkovic SS, Kerr CR, et al. A novel, simple scale for assessing the symptom severity of atrial fibrillation at the bedside: The CCS-SAF Scale. *The Canadian Journal of Cardiology*. 2006;22(5): 383-386.

2. EQ-5D value sets: Inventory, comparative review and user guide. Eds. Szende A, Oppe M, Devlin N. EuroQol Group Monographs Volume 2. Springer, 2006.

3. AFEQT Core Team-John Spertus, MD, Mid America Heart Institute, Kansa s City, MO; Paul Dorian, MD, St. Michaels Hospital, Toronto, ON; Rosemary Bubien, RN, University of Alabama, Birmingham, AL; Caroline Burk, Pharm D. M.S; Steven Lewis, PhD; Donna Godejohn, BSN, St. Jude Medical, St. Paul, MN. Atrial Fibrillation Effect on QualiTy-of-life (AFEQT) Questionnaire Instruction and Scoring Manual. Version 1.0. 2010 May 19:1-4.
