# Supplementary material for: Association of Atrial Fibrillation Episode Duration With Arrhythmia Recurrence Following Ablation: A Secondary Analysis of a Randomized Clinical Trial
Source: JAMA Netw Open. 2020 Jul 2;3(7):e208748. doi: 10.1001/jamanetworkopen.2020.8748 (PMC7333024; doi:10.1001/jamanetworkopen.2020.8748)
Supplement: Supplement 2. — eFigure. Study Flow Diagram [file jamanetwopen-3-e208748-s002.pdf]

Supplementary Online Content

Andrade JG, Deyell MW, Verma A, et al. Association of atrial fibrillation episode duration with arrhythmia recurrence following ablation: a secondary analysis of a randomized clinical trial. *JAMA Netw Open*. 2020;3(7):e208748. doi:10.1001/jamanetworkopen.2020.8748

eFigure. Study Flow Diagram

This supplementary material has been provided by the authors to give readers additional information about their work.

© 2020 Andrade JG et al. *JAMA Network Open.*

eFigure. Study Flow Diagram

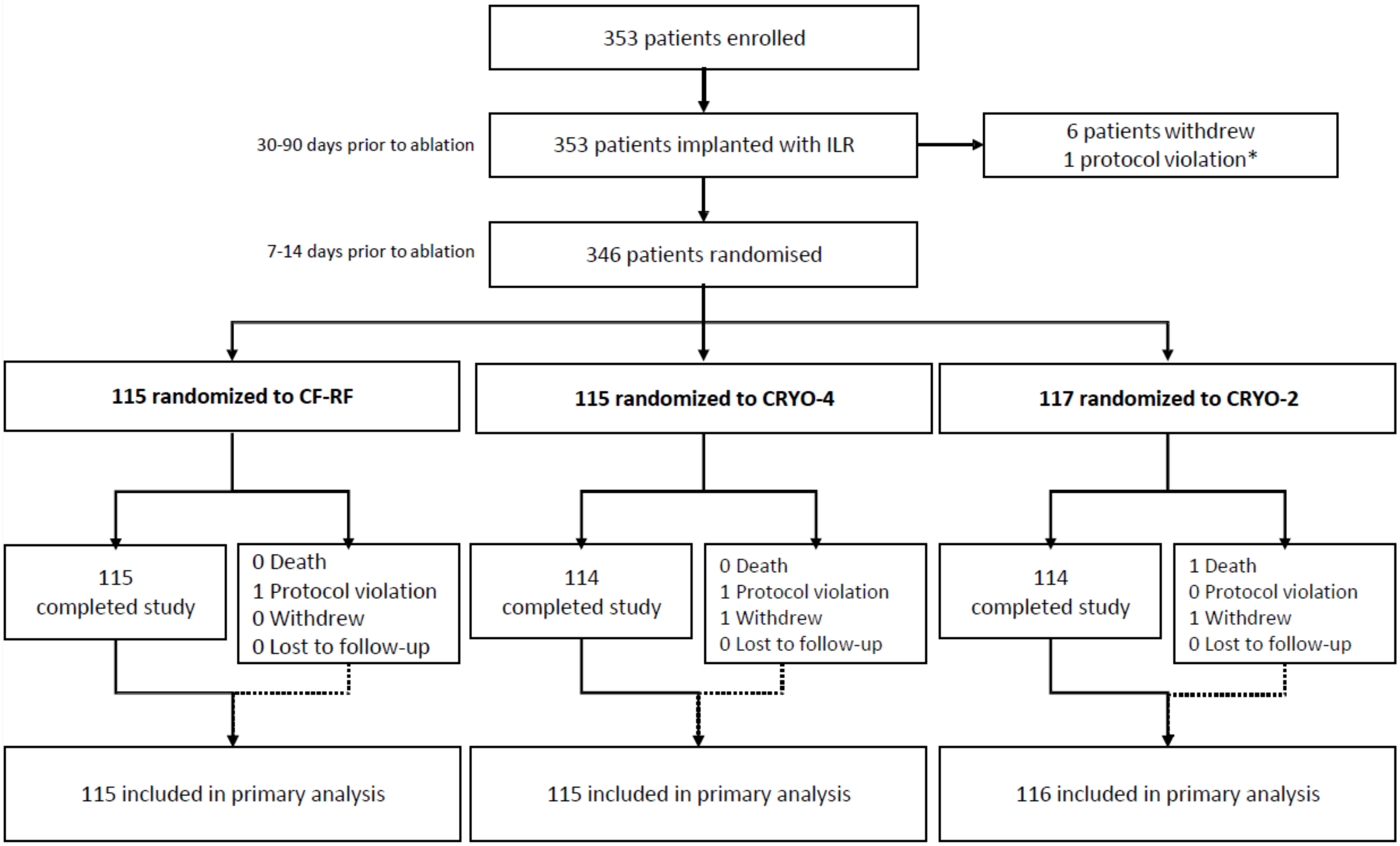

© 2020 Andrade JG et al. *JAMA Network Open.*
