# Supplementary material for: Association of Atrial Fibrillation Episode Duration With Arrhythmia Recurrence Following Ablation: A Secondary Analysis of a Randomized Clinical Trial
Source: JAMA Netw Open. 2020 Jul 2;3(7):e208748. doi: 10.1001/jamanetworkopen.2020.8748 (PMC7333024; doi:10.1001/jamanetworkopen.2020.8748)
Supplement: Supplement 3. — Data Sharing Statement [file jamanetwopen-3-e208748-s003.pdf]

# Data Sharing Statement

Andrade. Association of Atrial Fibrillation Episode Duration With Arrhythmia Recurrence Following Ablation: A Secondary Analysis of a Randomized Clinical Trial. *JAMA Netw Open*. Published July 2, 2020.
10.1001/jamanetworkopen.2020.8748

**Data**
**Data available:** Yes
**Data types:** Deidentified participant data
**How to access data:** jason.andrade@vch.ca
**When available:** December 31, 2020

**Supporting Documents**
**Document types:** None

**Additional Information**
**Who can access the data:** Reasonable data access requests will be considered by the steering committee
**Types of analyses:** Reasonable data access requests will be considered by the steering committee
**Mechanisms of data availability:** Reasonable data access requests will be considered by the steering committee
